# Asymmetric α‐Chlorination of β‐Keto Esters Using Hypervalent Iodine‐Based Cl‐Transfer Reagents in Combination with Cinchona Alkaloid Catalysts

**DOI:** 10.1002/ejoc.202001217

**Published:** 2020-09-25

**Authors:** Lotte Stockhammer, Johannes Schörgenhumer, Christopher Mairhofer, Mario Waser

**Affiliations:** ^1^ Institute of Organic Chemistry Johannes Kepler University Linz Altenbergerstr. 69 4040 Linz Austria

**Keywords:** Chlorination, Cinchona alkaloids, Hypervalent iodine reagents, Nucleophilic catalysis, Organocatalysis

## Abstract

We herein report an unprecedented strategy for the asymmetric α‐chlorination of β‐keto esters with hypervalent iodine‐based Cl‐transfer reagents using simple Cinchona alkaloid catalysts. Our investigations support an α‐chlorination mechanism where the Cinchona species serves as a nucleophilic catalyst by reacting with the chlorinating agent to generate a chiral electrophilic Cl‐transfer reagent in situ. Using at least 20 mol‐% of the alkaloid catalyst allows for good yields and enantioselectivities for a variety of different β‐keto esters under operationally simple conditions.

## Graphical abstract

Simple Cinchona alkaloids serve as nucleophilic organocatalysts to facilitate the enantioselective α‐chlorination of β‐keto esters by using hypervalent iodine‐based Cl‐transfer reagents

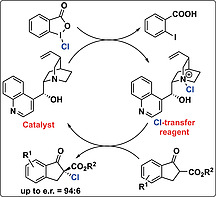

## Introduction

**Scheme 1:**
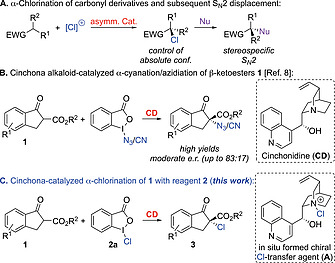
Asymmetric α‐chlorination of carbonyl derivatives to access valuable chiral building blocks (A), our recently developed asymmetric α‐azidation/cyanation of β‐keto esters **1** with hypervalent iodine reagents (B), and the herein investigated chlorination of compounds **1** with reagents **2** catalyzed by Cinchona alkaloids (C).

Catalytic enantioselective syntheses of chiral α‐chlorinated carbonyl derivatives represent important transformations, mainly because of the value of the hereby obtained enantioenriched products as building blocks or intermediates for a variety of further transformations.^[^ ^1^, ^2^ ^]^ In particular, these compounds can undergo highly stereospecific nucleophilic S_N_2‐type reactions allowing for the synthesis of valuable (biologically active) target molecules (Scheme 1A).^[^ ^2^, ^3^ ^]^ Accordingly, it comes as no surprise that the development of reliable enantioselective asymmetric approaches to access these targets in a catalytic fashion has been heavily investigated in the past.^[^ ^2^, ^4^, ^5^ ^]^ A common synthesis strategy relies on the addition of prochiral enolate equivalents to electrophilic Cl‐sources. Within this context, N‐chlorosuccinimide (NCS) emerged as one of the most commonly used formal Cl^+^‐sources over the last years, but other reagents turned out to be well‐suited too.^[^ ^2^ ^]^

Hypervalent iodine‐based electrophile‐transfer reagents have been established as powerful and broadly applicable tools in (asymmetric) organic synthesis.^[^ ^6^ ^]^ Our group has recently reported the use of these reagents in combination with asymmetric organocatalysis^[^ ^7^ ^]^ to access enantioenriched α‐CN‐ and α‐N_3_‐β‐keto esters in good yields and with moderate enantioselectivities (Scheme 1B).^[^ ^8^ ^]^ Simple Cinchona alkaloids^[^ ^9^ ^]^ (i.e. Cinchonidine, **CD**) turned out to be the best‐suited organocatalysts for our target reactions,^[^ ^8^ ^]^ but other research groups demonstrated impressively that alternative organocatalytic activation modes can be used efficiently to control reactions of the prochiral nucleophiles **1** with different hypervalent iodine‐based electrophile‐transfer reagents either.^[^ ^10^ ^]^

Interestingly, when we investigated the α‐azidation of β‐keto esters **1** ^[^ ^8b^ ^]^ we occasionally observed the formation of notable amounts of the α‐chlorinated β‐keto esters **3** when we quenched “slow” reactions that contained unconverted starting materials with an aqueous NaCl solution. Control experiments suggested that in these cases the well‐known Cl‐containing hypervalent iodine reagent **2a** is formed in situ,^[^ ^11^, ^12^ ^]^ which then serves as an electrophilic Cl‐transfer reagent.^[^ ^13^, ^14^ ^]^ In general, hypervalent iodine reagents have been investigated for halogenation reactions,^[^ ^15^ ^]^ but especially the use of reagents like **2a** for asymmetric α‐chlorinations of prochiral nucleophiles (e.g. compounds **1**) has so far been only sparingly investigated.^[^ ^13^, ^16^, ^17^ ^]^ Very promising, our first tests also showed encouraging levels of enantioselectivity (vide infra) when using simple Cinchona alkaloids as catalysts. Interestingly, during initial mass spectrometric analyses of the reaction mixture (containing β‐keto ester **1a** (R^1^ = H, R^2^ = *t*Bu), reagent **2a**, and Cinchonidine (**CD**)) we detected a species with *m/z* = 329.1414,^[^ ^18^ ^]^ which corresponds to an in situ formed [**CD** + Cl]^+^ intermediate like compound **A** (calculated *m/z* = 329.1415), which most presumably serves as the chiral Cl‐transfer reagent (vide infra). While analogous N‐fluoro Cinchona‐based ammonium salts have been well described as asymmetric electrophilic fluorination agents,^[^ ^19^ ^]^ the presence and utilization of such potentially useful Cl‐transfer species have so far received very little attention only.^[^ ^20^, ^21^ ^]^ Given this interesting mechanistic aspect, as well as the encouraging initial enantioselectivities observed for the chlorination of **1a**, we became interested in investigating the general methodology outlined in Scheme 1C in more detail.

## Results and Discussion

**Figure 1:**
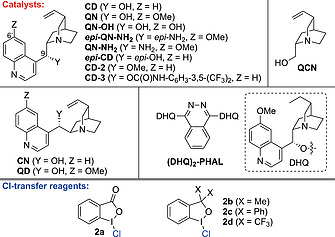
Catalysts and Cl‐transfer reagents used herein.

We started our investigations by carrying out the α‐chlorination of the *tert*‐butyl ester **1a** with the catalysts and Cl‐transfer reagents depicted in Figure 1 (Table 1 gives an illustrative overview of the most significant results obtained in this screening).

**Table 1:** Screening and optimization of reaction conditions^[^ a ^]^

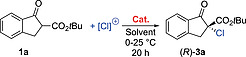					
Entry	Cat.	[Cl]^+^	Solv.	Yield [%]^[^ b ^]^	*e.r*. (*R:S*)^[^ c ^]^
1	**–**	**2a** (2x)	toluene	75	–
2	**CD** (10 %)	**2a** (2x)	toluene	81	77:23
3	**CD** (20 %)	**2a** (2x)	toluene	79	83:17
4	**CD** (40 %)	**2a** (2x)	toluene	81	85:15
5	**CD** (100 %)	**2a** (2x)	toluene	78	94:6
6	**CD** (40 %)	**2a** (2x)	CH_2_Cl_2_	79	55:45
7	**CD** (40 %)	**2a** (2x)	THF	68	56:44
8	**QN** (40 %)	**2a** (2x)	toluene	74	75:25
9	**CN** (40 %)	**2a** (2x)	toluene	85	27:73
10	**QD** (40 %)	**2a** (2x)	toluene	72	40:60
11	**CD‐2** (40 %)	**2a** (2x)	toluene	89	54:46
12	**CD‐3** (40 %)	**2a** (2x)	toluene	73	72:28
13	**QN‐OH** (40 %)	**2a** (2x)	toluene	68	53:47
14	***epi*‐CD** (40 %)	**2a** (2x)	toluene	67	64:36
15	***epi*‐QN‐NH_2_** (40 %)	**2a** (2x)	toluene	42	51:49
16	**QN‐NH_2_** (40 %)	**2a** (2x)	toluene	39	55:45
17	**QCN** (40 %)	**2a** (2x)	toluene	<10	50:50
18	**(DHQ)_2_PHAL** (40 %)	**2a** (2x)	toluene	79	52:48
19	**CD** (40 %)	**2b** (2x)	toluene	18	58:42
20	**CD** (40 %)	**2c** (2x)	toluene	38	67:33
21	**CD** (40 %)	**2d** (2x)	toluene	46	79:21
22	**CD** (40 %)	**2a** (1 equiv.)^[^ d ^]^	toluene	80	93:7
23	**CD** (20 %)	**2a** (1 equiv.)^[^ d ^]^	toluene	74	90:10

a All reactions were carried out using 0.1 mmol **1a** in the indicated solvent (0.3 m with respect to **1a**). Unless otherwise stated, reactions were started at 0 °C (ice bath) and warmed to 25 °C over 20 h.

b Isolated yields.

c Determined by HPLC using a chiral stationary phase. The absolute configuration was assigned by comparison of HPLC retention order with previous reports.^[^ ^5^ ^]^

d Added in 4 portions in 2 h intervals.

First experiments in the absence of any catalyst or base showed that the racemic α‐chlorination of **1a** with **2a** (2 equiv.) is a surprisingly efficient process (entry 1). When adding different amounts of Cinchonidine (**CD**; entries 2–5), it was found that increasing amounts of the chiral amine base are clearly beneficial to obtain reasonable levels of enantioselectivity, which can be rationalized by the fast uncatalyzed racemic background reaction. Interestingly, the use of non‐aromatic solvents leads to an almost racemic outcome (entries 6 and 7). When testing other natural Cinchona alkaloids as well as synthetic derivatives thereof (entries 8–18), it became obvious that the reaction is very sensitive to the decoration of the Cinchona skeleton. Functional groups other than a proton in the 6'‐position (e.g. entries 8, 10, or 13) are not well‐accepted, and the same comes true when alkylating the 9‐OH‐group (entry 11). Also, the truncated amine **QCN** (entry 17) or the bis‐alkaloid **(DHQ)_2_PHAL** (entry 18) were not suited.

As these results showed that the presence of an H‐bonding donor in the 9‐position is crucial, we also tested other Cinchona derivatives with alternating H‐bonding motives and retained (entries 12 and 16) or inverted C9‐configuration (entries 14 and 15), but neither of those catalyst systems allowed for comparable results as obtained with the naturally occurring **CD**. This demonstrates that the native configuration is crucial and that the 9‐OH plays a role in the stereodefining step. Unfortunately, the diastereomeric **CN** does not fully match the selectivity for (*S*)‐**3a** as compared to **CD** for (*R*)‐**3a** (entry 4 vs. 9). When using the alternative Cl‐transfer reagents **2b**‐**d** next (entries 19–21), it was found that none of them allows for a similar selectivity as **2a**. This may be rationalized by looking at the species that are formed from compounds **2a**‐**d** after the Cl‐transfer. While **2a** forms a weakly basic carboxylate species, **2b**‐**d** form more basic benzylic alcohol species, which then most likely speed up the racemic background reaction. Accordingly, the already initially used combination of **CD** with **2a** was found to be the best‐suited catalyst/Cl‐transfer reagent system among all the tested ones. Unfortunately, however, the racemic background reaction was found to be relatively fast under all conditions, and the only strategy to somehow suppress it was by using just one equivalent of **2a** and by adding it portion‐wise over a longer period of time, which finally resulted in reliable reaction conditions that delivered the α‐chlorinated product **3a** with good levels of enantioselectivity in combination with reasonably small quantities of the chiral Cinchona alkaloid catalyst **CD** (entries 22 and 23).

As initially stated (vide supra), we first observed this α‐chlorination during our work on the α‐cyanation of β‐keto esters **1**, where reagent **2a** was presumably formed in situ from other hypervalent iodine species and NaCl. We thus also carried out test experiments with **1a** and the corresponding acetate‐analog of iodine reagent **2a** (OAc instead of Cl) in the presence of **CD** and NaCl. Interestingly, product **3a** was formed in reasonable quantities, thus supporting our hypothesis, but the enantioselectivity was rather low only (*e.r*. < 65:35), thus substantiating a rather fast racemic background reaction.

**Figure 2:**
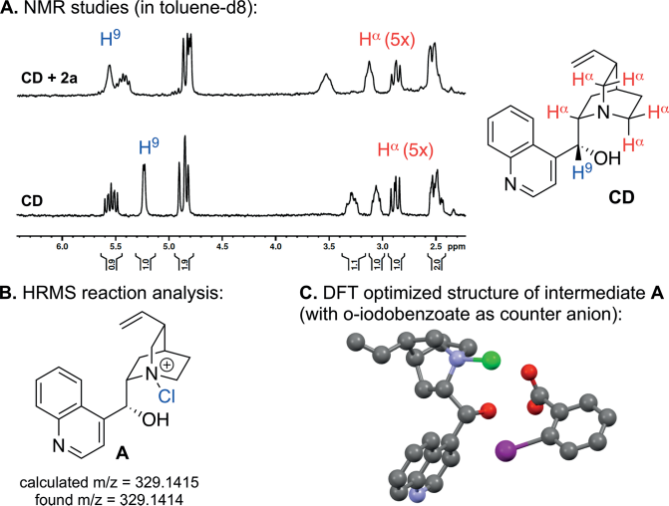
NMR (A), HRMS (B), and DFT (C) investigations to obtain further insights into the role of the catalyst **CD** in the asymmetric α‐chlorination of **1a** with reagent **2a**.

As mentioned above, we observed the formation of a [**CD** + Cl]^+^ species when analyzing the reaction mixture by HRMS (see Figure 2B for the exact values). Based on this observation, and the fact that the 9‐OH group plays a crucial role in the asymmetric α‐chlorination of **1a**, our proposal is that the quinuclidine nitrogen of **CD** will be chlorinated under the reaction conditions and that this species **A** then serves as the electrophilic Cl‐transfer reagent (as is well‐established for the analogous F‐containing Cinchona species^[^ ^19^ ^]^). However, an alternative option would also be the chlorination of the quinoline nitrogen. To get further insights, we carried out ^1^H NMR studies of **CD** mixed with **2a**, which showed significant shifting of the signals of the protons α‐ to the amine functionality (H^α^) and the proton in the benzylic position (H^9^) (Figure 2A), thus substantiating chlorination in this pocket of **CD**. These results are also in line with recent studies by Hennecke's group on the asymmetric Cinchona alkaloid‐catalyzed dichlorination of alkenes.^[^ ^21^ ^]^ In addition, DFT calculations suggest that this species **A** (Figure 2C) is more stable than the alternative with the quinoline nitrogen being chlorinated and we thus propose that **CD** actually serves as a chiral Cl‐shuttle herein. Noteworthy, the hereby formed [**CD** + Cl]^+^ species was found to be significantly less stable than the well‐established F‐reagents^[^ ^19^ ^]^ and degraded within less than 1 h during these NMR investigations, making further characterization and isolation of this interesting species not possible.

**Scheme 2:**
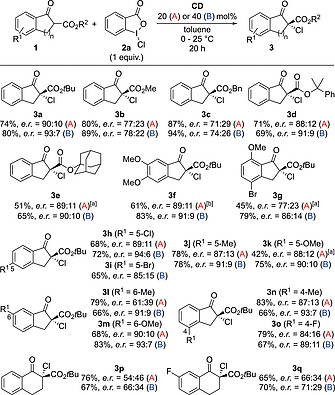
Application scope (0.1 mmol scale; unless otherwise stated more than 90 % conversion of **1** after 20 h): [a] Less than 60 % conversion of **1** after 20 h; [b] Less than 80 % conversion of **1** after 20 h.

Finally, we tested the application scope of this α‐chlorination protocol by applying the optimized conditions (using 20 and/or 40 mol‐% of **CD**) to a variety of differently substituted β‐keto esters **1** (Scheme 2).

In general, this methodology was found to be rather robust for a variety of differently substituted starting materials **1**, although in some special cases conversion was not complete within 20 h, especially when using only 20 mol‐% of the catalyst (see the details given for products **3e**, **3f**, and **3g**). As for the parent system **3a**, the use of 40 mol‐% CD usually resulted in higher enantioselectivities. Besides indanone‐derived keto esters also the tetralone‐based products **3p** and **3q** were accessible, albeit with lower selectivities and the products turned out to be relatively unstable as well (one acyclic derivative was tested as well but did not react at all).

## Conclusions

In conclusion, we have developed an operationally simple protocol for the asymmetric α‐chlorination of cyclic β‐keto esters using an easily accessible hypervalent iodine‐based Cl‐transfer reagent in combination with naturally occurring Cinchona alkaloid catalysts. Noteworthy, the Cinchona alkaloid most likely acts as a nucleophilic catalyst by forming an N‐chlorinated Cinchona species in situ (upon reaction with the Cl‐reagent), which then serves as the actual chiral Cl‐transfer species. While analogous N‐fluorinated derivatives have been described and utilized before,^[^ ^19^ ^]^ the N‐chlorinated species described herein have so far received very little attention,^[^ ^20^, ^21^ ^]^ and future studies will focus on a more general understanding and the use of this interesting catalysis concept for other target transformations.

## Experimental Section

General details, analytical details of known compounds, computational details, copies of NMR spectra, and HPLC traces can be found in the online supporting information.

**General Asymmetric α‐Chlorination Procedure:** A mixture of the respective β‐keto ester **1** (0.1 mmol) and cinchonidine **CD** (cond. A: 20 mol‐%; cond. B: 40 mol‐%) in 3 mL anhydrous toluene was cooled to 0 °C. Then, benziodoxolone **2a** (1 equiv.) was added to the stirred solution in four portions (2 h intervals). The mixture was left to reach r.t. overnight and then quenched with 3 mL deionized water (20 h total reaction time). The layers were separated, and the aqueous layer was washed with DCM (3 × ). The combined organic phases were washed with deionized water, dried with Na_2_SO_4,_ and concentrated on the rotary evaporator. The crude product was purified by column chromatography (silica, DCM/heptanes = 5:1) giving products **3** in the indicated yields and with the reported enantioselectivities.

**Analytical details of new products 3 (details of the known products can be found in the online supporting information):**

**Product 3g:** Obtained as a yellowish oil after silica gel column chromatography using DCM/heptanes (5:1). 45 % yield and *e.r*. = 77:23 (conditions A); 79 % yield and *e.r*. = 86:14 (conditions B); TLC (DCM/heptanes = 5:1): *R*_f_ = 0.31. [*α*]_D_^23^ = –23.9 (c 1, CHCl_3_, *e.r*. = 86:14); ^1^H‐NMR (300 MHz, CDCl_3_, 298.0 K): δ/ppm = 1.45 (s, 9 H), 3.39 (d, *J* = 18.3 Hz, 1 H), 3.85 (d, *J* = 18.4 Hz, 1 H), 3.96 (s, 3 H), 6.81 (d, *J* = 8.8 Hz, 1 H), 7.73 (d, *J* = 8.8 Hz, 1 H); ^13^C‐NMR (75 MHz, CDCl_3_, 298.0 K): δ/ppm = 27.7, 44.1, 56.3, 68.6, 84.6, 110.8, 112.2, 122.6, 140.2, 151.9, 158.7, 165.6, 192.4; HRMS (ESI) *m/z*: calcd. for [C_15_H_16_BrClO_4_ + NH_4_]^+^: 392.0258, found 392.0266, HPLC: Chiralcel OD‐H, *n*‐hexane/*i*PrOH = 10:1, 0.5 mL/min, 10 °C; *t*_R_ = 18.4 min [major], 24.8 min [minor].

**Product 3j:** Obtained as a yellow residue after silica gel column chromatography using DCM/heptanes (5:1). 78 % yield and *e.r*. = 87:13 (conditions A); 78 % yield and *e.r*. = 91:9 (conditions B); TLC (DCM/heptanes = 5:1): *R*_f_ = 0.50. [*α*]_D_^23^ = –33.6 (c 1, CHCl_3_, *e.r*. = 91:9); ^1^H‐NMR (300 MHz, CDCl_3_, 298.0 K): δ/ppm = 1.42 (s, 9 H), 2.47 (s, 3 H), 3.47 (d, *J* = 17.7 Hz, 1 H), 3.96 (d, *J* = 17.7 Hz, 1 H), 7.24–7.26 (m, 2 H), 7.73 (d, *J* = 8.2 Hz, 1H); ^13^C‐NMR (75 MHz, CDCl_3_, 298.0 K): δ/ppm = 22.2, 27.7, 43.3, 69.1, 84.2, 125.6, 126.5, 129.7, 130.5, 147.8, 151.1, 166.0, 195.0; HRMS (ESI) *m/z*: calcd. for [C_15_H_17_ClO_3_ + NH_4_]^+^: 298.1204, found 298.1213, HPLC: Chiralpak AD‐H, *n*‐hexane/*i*PrOH = 10:1, 0.5 mL/min, 10 °C; *t*_R_ = 13.8 min [minor], 14.7 min [major].

**Product 3o:** Obtained as white needles after silica gel column chromatography using DCM/heptanes (5:1). 79 % yield and *e.r*. = 84:16 (conditions A); 67 % yield and *e.r*. = 89:11 (conditions B); TLC (DCM/heptanes = 5:1): *R*_f_ = 0.24. [*α*]_D_^23^ = –28.2 (c 1, CHCl_3_, *e.r*. = 89:11); ^1^H‐NMR (300 MHz, CDCl_3_, 298.0 K): δ/ppm = 1.39 (s, 9 H), 3.48 (d, *J* = 18.1 Hz, 1 H), 3.96 (d, *J* = 18.1 Hz, 1 H), 7.29–7.34 (m, 1 H), 7.38–7.45 (m, 1 H), 7.62 (d, *J* = 7.5 Hz, 1 H); ^13^C‐NMR (75 MHz, CDCl_3_, 298.0 K): δ/ppm = 27.7, 43.1, 68.6, 84.7, 121.5 (d, *J* = 4.1 Hz), 122.3 (d, *J* = 19.8 Hz), 130.5 (d, *J* = 6.1 Hz), 135.4 (d, *J* = 4.4 Hz), 136.6 (d, *J* = 19.7 Hz), 159.4 (d, *J* = 251.5 Hz), 165.4, 194.5; HRMS (ESI) *m/z*: calcd. for [C_14_H_14_ClFO_3_ + NH_4_]^+^: 302.0954, found 302.0963, HPLC: Chiralcel OD‐H, *n*‐hexane/*i*PrOH = 250:1, 0.5 mL/min, 10 °C; *t*_R_ = 19.9 min [major], 21.2 min [minor].

**Product 3q:** Obtained as a slightly brown oil after silica gel column chromatography using DCM/heptanes (5:1). 65 % yield and *e.r*. = 66:34 (conditions A); 70 % yield and *e.r*. = 71:29 (conditions B); TLC (DCM/heptanes = 5:1): *R*_f_ = 0.24. [*α*]_D_^23^ = –28.2 (c 1, CHCl_3_, *e.r*. = 71:29); ^1^H‐NMR (300 MHz, CDCl_3_, 298.0 K): δ/ppm = 1.46 (s, 9 H), 2.46–2.55 (m, 1 H), 2.88–3.03 (m, 2 H), 3.15–3.25 (m, 1 H), 7.23–7.26 (m, 2 H), 7.72–7.76 (m, 1 H); ^13^C‐NMR (75 MHz, CDCl_3_, 298.0 K): δ/ppm = 25.3, 27.7, 35.3, 71.1, 84.4, 114.5 (d, *J* = 22.3 Hz), 121.7 (d, *J* = 22.2 Hz), 130.6 (7.2 Hz, 1 C), 131.7 (d, *J* = 7.7 Hz), 138.1 (d, *J* = 3.0 Hz), 161.7 (d, *J* = 247.9 Hz), 165.9, 187.1; HRMS (ESI) *m/z*: calcd. for [C_15_H_16_ClFO_3_ + H]^+^: 299.0845, found 299.0850, HPLC: Chiralpak AD‐H, *n*‐hexane/*i*PrOH = 10:1, 0.5 mL/min, 10 °C; *t*_R_ = 10.0 min [major], 10.5 min [minor].

## Supporting information

Supporting Information
