# Supplementary material for: Asymmetric α‐Chlorination of β‐Keto Esters Using Hypervalent Iodine‐Based Cl‐Transfer Reagents in Combination with Cinchona Alkaloid Catalysts
Source: European J Org Chem. 2020 Sep 25;2021(1):82–6. doi: 10.1002/ejoc.202001217 (PMC7821243; doi:10.1002/ejoc.202001217)
Supplement: Supplementary file 1 — Supporting Information [file EJOC-2021-82-s001.pdf]

European Journal of Organic Chemistry

Supporting Information

Asymmetric α-Chlorination of β-Keto Esters Using Hypervalent Iodine-Based Cl-Transfer Reagents in Combination with Cinchona Alkaloid Catalysts

Lotte Stockhammer, Johannes Schörgenhumer, Christopher Mairhofer, Mario Waser*

1. General Information: 1
2. Asymmetric α-Chlorination Reactions: 2
3. HRMS of Complex A 9
4. Computational details for Complex A 10
5. NMR Spectra of New and Representative Known Chlorinated Products: 12
6. HPLC Traces of Chlorinated Products: 27

1. General Information:

NMR spectra were recorded on a Bruker Avance III 300 MHz spectrometer with a broad band observe probe and a sample changer for 16 samples which is property to the Austro Czech NMR Research Center "RERI uasb". All NMR spectra were referenced on the solvent residual peak ($CDCl_3$: δ 7.26 ppm for ^{1}H NMR and δ 77.16 ppm for ^{13}C NMR). NMR data are reported as follows: chemical shift (δ ppm), multiplicity (s = singlet, d = doublet, t = triplet, q = quartet, m = multiplet, br = broad), coupling constants (Hz) and integrals. High resolution mass spectra were obtained using an Thermo Fisher Scientific LTQ Orbitrap XL hybrid FT mass spectrometer with an ESI source and an Agilent G1607A coaxial sprayer. Low resolution mass spectra were obtained using an Agilent LC/MSD Trap SL. Optical rotations were measured on a Schmidt+Haensch Unipol L 100 polarimeter ($[\alpha]_D$ values are listed in $deg \cdot cm^3 \cdot g^{-1} \cdot dm^{-1}$; concentration *c* is given in g/100 mL). Preparative column chromatography was carried out using Davisil LC 60A 70–200 MICRON silica gel. Thin layer chromatography was performed on Macherey-Nagel pre-coated TLC plates (silica gel, 60 F_{254}, 0.20 mm, ALUGRAM® Xtra SIL). TLC plates were visualized under 254 nm UV lamp. Enantiomeric ratios (*e.r.*) were determined by HPLC analysis using a Dionex Summit HPLC system with a CHIRALCEL OD-H (4.6 mm× 250 mm, 5 µm), CHIRALCEL OJ-H (4.6 mm x 250 mm x 5 µm)and a CHIRALPAK AD-H (4.6 mm × 250 mm, 5 µm) chiral stationary phase. All chemicals were purchased from commercial suppliers and used without further purification unless otherwise stated. All reactions were carried out under an argon atmosphere. Starting β-ketoesters **1**[1] and hypervalent iodine reagents **2**[2] were prepared using established procedures.

(1) (a) T. A. Moss, D. R. Fenwick, D. J. Dixon, *J. Am. Chem. Soc.* **2008**, *130*, 10076. (b) D. Y. Kim, E. J. Park, *Org. Lett.* **2002**, *4*, 545. (c) X. Wang, Q. Lan, S. Shirakawa, K. Maruoka, *Chem. Commun.* **2010**, *46*, 321. (d) E.-M. Tanzer, W. B. Schweizer, M.-O. Ebert, R. Gilmour, *Chem. Eur. J.* **2012**, *18*, 2006. (e) M. Lian, J. Du, Q. Meng, Z. Gao, *Eur. J. Org. Chem.* **2010**, *34*, 6525.
(2) V. Matoušek, E. Pietrasiak, R. Schwenk, A. Togni, *J. Org. Chem.*, **2013**, *78, 13*, 6763–6768

2. Asymmetric α-Chlorination Reactions:

General procedure: A mixture of the respective ketoester **1** (0.1 mmol) and cinchonidine CD (cond. A: 20 mol%; cond. B: 40 mol%) in 3 mL anhydrous toluene was cooled to 0 °C. Then, benziodoxolone **2a** (1 eq.) was added to the stirred solution in four portions (2 h intervals). The mixture was left to reach r.t. overnight and then quenched with 3 mL deionized water. The layers were separated, and the aqueous layer was washed with DCM (3x). The combined organic phases were washed with deionized water, dried over Na_2SO_4 and concentrated on the rotary evaporator. The crude product was purified by column chromatography (silica, DCM/heptanes = 5/1).

Characterization Data for α-chlorinated ketoesters 3a–q

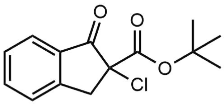

3a

3a was obtained as a white crystalline solid after silica gel column chromatography using DCM/heptanes (5:1). ***e.r.*** = 10:90 (conditions A), 7:93 (conditions B); TLC (DCM/heptanes = 5/1): $\boldsymbol{R_f}$ = 0.47 (UV). Analytical data are in accordance with those reported in literature[3]. $[\alpha]_D^{23}$ = -26.9 (*c* 1, $CHCl_3$, 10:90 *e.r.*, A), -27.6 (*c* 1, $CHCl_3$, 7:93 *e.r.*, B); **^{1}H-NMR** (300 MHz, CDC13, 298.0 K): δ / ppm = 1.43 (s, 9 H, -C**H**$_3$), 3.53 (d, J = 17.7 Hz, 1 H, -C**H**$_2$), 4.01 (d, J = 17.7 Hz, 1 H, -C**H**$_2$), 7.43-7.48 (m, 2 H, Ar-**H**), 7.68 (t, J = 7.9 Hz, 1 H, Ar-**H**), 7.85 (d, J = 7.4 Hz, Ar-**H**); **^{13}C-NMR** (75 MHz, $CDCl_3$, 298.0 K): δ / ppm = 27.7 (3 C, -**C**H$_3$), 43.5 (1 C, -**C**H$_2$), 68.7 (1 C, $\mathbf{C_q}$), 84.4 (1 C, $\mathbf{C_q}$**)**, 125.8 (1 C, $\mathbf{C_{Ar}}$), 126.2 (1 C, $\mathbf{C_{Ar}}$), 128.4 (1 C, $\mathbf{C_{Ar}}$), 132.8 (1 C, $\mathbf{C_{Ar}}$), 136.1 (1 C, $\mathbf{C_{Ar}}$), 150.6 (1 C, $\mathbf{C_{Ar}}$), 165.9 (1 C, -**C**=O), 195.5 (1 C, -**C**=O); **MS** (ESI) *m/z*: calcd for $[C_{14}H_{15}ClO_3 + NH_4]^+$: 284.10; found: 284.30, **HPLC:** Chiralcel OJ-H, *n*-hexane/*i*-PrOH 3:1, 1.0 mL/min, 10 °C; t_R = 7.3 min [minor], 9.2 min [major].

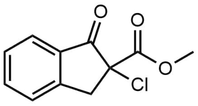

3b

3b was obtained as an oily yellow residue after silica gel column chromatography using DCM/heptanes (5:1). ***e.r.*** = 23:77 (conditions A), 22:78 (conditions B); TLC (DCM/heptanes = 5/1): $\boldsymbol{R_f}$ = 0.32 (UV). Analytical data are in accordance with those reported in literature[3]. $[\alpha]_D^{23}$ = -19.6 (*c* 1, $CHCl_3$, 23:77 *e.r.*, A), -20.0 (*c* 1, $CHCl_3$, 22:78 *e.r.*, B); **^{1}H-NMR** (300 MHz, CDC13, 298.0 K): δ / ppm = 3.53 (d, J = 17.9 Hz, 1 H, -C**H**$_2$), 3.82 (s, 1 H, -C**H**$_3$), 4.11 (d, J = 17.8 Hz, 1 H, -C**H**$_2$), 7.45-7.50 (m, 2 H, Ar-**H**), 7.70 (t, J = 8.1 Hz, 1 H, Ar-**H**), 7.86 (d, J = 7.7 Hz, 1 H, Ar-**H**); **^{13}C-NMR** (75 MHz, $CDCl_3$, 298.0 K): δ / ppm = 43.4 (1 C, -**C**H$_3$), 54.1 (1 C, -**C**H$_2$) , 67.8 (1 C, $\mathbf{C_q}$), 126.0 (1 C, $\mathbf{C_{Ar}}$), 126.3 (1 C, $\mathbf{C_{Ar}}$), 128.6 (1 C, $\mathbf{C_{Ar}}$), 132.4 (1 C, $\mathbf{C_{Ar}}$), 136.5 (1 C, $\mathbf{C_{Ar}}$), 150.5 (1 C, $\mathbf{C_{Ar}}$), 167.6 (1 C, -**C**=O), 195.0 (1 C, -**C**=O); **MS** (ESI) *m/z*: calcd for $[C_{11}H_9ClO_3 + NH_4]^+$: 242.06; found: 242.25, **HPLC:** Chiralcel OJ-H, *n*-hexane/*i*-PrOH 3:1, 1.0 mL/min, 10 °C; t_R = 21.3 min [minor], 25.3 min [major].

(3) J. Novacek, U. Monkowius, M. Himmelsbach, M. Waser, *Monatsh. Chem.*, **2016**, *147*, 533–538.

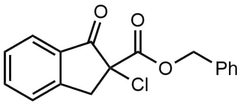

3c

3c was obtained as a slightly brown white solid after silica gel column chromatography using DCM/heptanes (5:1). ***e.r.*** = 29:71 (conditions A), 26:74 (conditions B); TLC (DCM/heptanes = 5/1): $\boldsymbol{R_f}$ = 0.43 (UV). Analytical data are in accordance with those reported in literature[3]. $[\alpha]_D^{23}$ = -20.5 (*c* 1, $CHCl_3$, 29:71 *e.r.*, A), -21.6 (*c* 1, $CHCl_3$, 26:74 *e.r.*, B); **^{1}H-NMR** (300 MHz, $CDCl_3$, 298.0 K): δ / ppm = 3.56 (d, J = 17.8 Hz, 1 H, **-CH₂**), 4.07 (d, J = 17.8 Hz, 1 H, **-CH₂**), 5.24 (d, J = 5.4 Hz, 2 H, **-CH₂**), 7.26-7.34 (m, 5 H, Ar-**H**), 7.45-7.49 (m, 2 H, Ar-**H**), 7.69 (d, J = 7.5 Hz, 1 H, Ar-**H**), 7.86 (d, J = 8.1 Hz, 1 H, Ar-**H**); **^{13}C-NMR** (75 MHz, $CDCl_3$, 298.0 K): δ / ppm = 43.3 (1 C, $\mathbf{C_q}$), 68.0 (1 C, -CH_2), 68.6 (1 C, -$\mathbf{CH_2}$), 126.0 (1 C, $\mathbf{C_{Ar}}$), 126.3 (1 C, $\mathbf{C_{Ar}}$), 127.9 (2 C, $\mathbf{C_{Ar}}$), 128.5 (1 C, $\mathbf{C_{Ar}}$), 128.6 (2 C, $\mathbf{C_{Ar}}$), 132.5 (1 C, $\mathbf{C_{Ar}}$), 134.7 (1 C, $\mathbf{C_{Ar}}$), 136.4 (1 C, $\mathbf{C_{Ar}}$), 150.5 (1 C, $\mathbf{C_{Ar}}$), 167.0 (1 C, -**C**=O), 194.8 (1 C, -**C**=O); **MS** (ESI) *m/z*: calcd for $[C_{17}H_{13}ClO_3 + NH_4]^+$: 318.10; found: 318.30, **HPLC:** Chiralcel OJ-H, *n*-hexane/*i*-PrOH 3:1, 1.0 mL/min, 10 °C; t_R = 30.0 min [minor], 47.6 min [major].

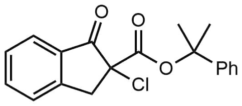

3d

3d was obtained as a yellow solid after silica gel column chromatography using DCM/heptanes (5:1). ***e.r.*** = 12:88 (conditions A), 9:91 (conditions B); TLC (DCM/heptanes = 5/1): $\boldsymbol{R_f}$ = 0.50 (UV). Analytical data are in accordance with those reported in literature[3]. **^{1}H-NMR** (300 MHz, $CDCl_3$, 298.0 K): δ / ppm = 1.75 (s, 1 H, -$\mathbf{CH_3}$), 1.76 (s, 1 H, -$\mathbf{CH_3}$), 3.52 (d, J = 17.7 Hz, 1 H, -$\mathbf{CH_2}$), 4.04 (d, J = 17.8 Hz, 2 H, -$\mathbf{CH_2}$), 7.16-7.28 (m, 5 H, Ar-**H**), 7.43-7.47 (m, 2 H, Ar-**H**), 7.65-7.70 (m, 1 H, Ar-**H**), 7.86 (d, J = 8.1 Hz, 1 H, Ar-**H**); **^{13}C-NMR** (75 MHz, $CDCl_3$, 298.0 K): δ / ppm = 28.1 (1 C, -CH_3), 28.1 (1 C, -CH_3), 43.3 (1 C, -$\mathbf{CH_2}$), 68.5 (1 C, $\mathbf{C_q}$), 85.4 (1 C, $\mathbf{C_q}$), 124.1 (1 C, $\mathbf{C_{Ar}}$), 125.0 (1 C, $\mathbf{C_{Ar}}$), 125.8 (1 C, $\mathbf{C_{Ar}}$), 126.2 (1 C, $\mathbf{C_{Ar}}$), 127.4 (1 C, $\mathbf{C_{Ar}}$), 128.3 (1 C, $\mathbf{C_{Ar}}$), 128.5 (1 C, $\mathbf{C_{Ar}}$), 132.8 (1 C, $\mathbf{C_{Ar}}$), 136.1 (1 C, $\mathbf{C_{Ar}}$), 136.2 (1 C, $\mathbf{C_{Ar}}$), 144.5 (1 C, $\mathbf{C_{Ar}}$), 150.6 (1 C, $\mathbf{C_{Ar}}$), 165.2 (1 C, -**C**=O), 195.3 (1 C, -**C**=O); **MS** (ESI) *m/z*: calcd for $[C_{19}H_{17}ClO_3 + NH_4]^+$: 346.12; found: 346.40, **HPLC:** Chiralcel OJ-H, *n*-hexane/*i*-PrOH 3:1, 1.0 mL/min, 10 °C; t_R = 19.7 min [minor], 24.11 min [major].

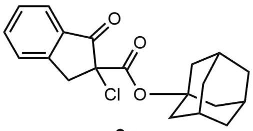

3e

3e was obtained as a yellowish white oil after silica gel column chromatography using DCM/heptanes (5:1). ***e.r.*** = 11:89 (conditions A), 10:90 (conditions B); TLC (DCM/heptanes = 5/1): $\boldsymbol{R_f}$ = 0.51 (UV). Analytical data are in accordance with those reported in literature[3]. $[\alpha]_D^{23}$ = -17.7 (*c* 1, $CHCl_3$, 11:89 *e.r.*, A), -21.2 (*c* 1, $CHCl_3$, 10:90 *e.r.*, B); **^{1}H-NMR** (300 MHz, $CDCl_3$, 298.0 K): δ / ppm = 1.61-1.63 (m, 6 H, -$\mathbf{CH_2}$), 2.04-2.05 (m, 6 H, -$\mathbf{CH_2}$), 2.14 (s, 3 H, -**CH**), 3.53 (d, J = 17.7 Hz, 1 H, -$\mathbf{CH_2}$), 4.02 (d, J = 17.7 Hz, 1 H, -$\mathbf{CH_2}$), 7.43-7.48 (m, 2 H, Ar-**H**), 7.68 (t, J = 7.0 Hz, 1 H, Ar-**H**), 7.85 (d, J = 7.4 Hz, 1 H, Ar-**H**); **^{13}C-NMR** (75 MHz, $CDCl_3$, 298.0 K): δ / ppm = 30.9 (3 C, -CH_2), 35.9 (3 C, -CH), 40.9 (3 C, -CH_2), 43.6 (1 C, -CH_2), 68.8 (1 C, $\mathbf{C_q}$), 84.4 (1 C, $\mathbf{C_q}$), 125.8 (1 C, $\mathbf{C_{Ar}}$), 126.2 (1 C, $\mathbf{C_{Ar}}$), 128.4 (1 C, $\mathbf{C_{Ar}}$), 132.9 (1 C, $\mathbf{C_{Ar}}$), 136.1 (1 C, $\mathbf{C_{Ar}}$), 150.7 (1 C, $\mathbf{C_{Ar}}$), 165.4 (1 C, -**C**=O), 195.6 (1 C, -**C**=O); **MS** (ESI)

m/*z*: calcd for $[C_{20}H_{21}ClO_3 + NH_4]^+$: 362.15; found: 362.35, **HPLC:** Chiralpak AD-H, *n*-hexane/*i*-PrOH 10:1, 0.5 mL/min, 10 °C; t_R = 16.8 min [minor], 21.2 min [major].

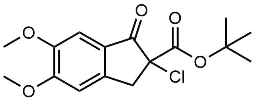

3f

3f was obtained as a colourless oil after silica gel column chromatography using DCM/heptanes (5:1). ***e.r.*** = 89:11 (conditions A), 91:9 (conditions B); TLC (DCM/heptanes = 5/1): R_f = 0.26 (UV). Analytical data are in accordance with those reported in literature[4]. $[\alpha]_D^{23}$ = -23.4 (*c* 1, $CHCl_3$, 91:9 *e.r.*, B); **^{1}H-NMR** (300 MHz, $CDCl_3$, 298.0 K): δ / ppm = 1.44 (s, 9 H, -**CH$_3$**), 3.43 (d, J = 17.5 Hz, 1 H, -**CH$_2$**), 3.92 (s, 3 H, -OC**H$_3$**), 3.93 (d, J = 16.9 Hz, 1 H, -**CH$_2$**), 3.99 (s, 3 H, -OC**H$_3$**), 6.86 (s, 1 H, Ar-**H**), 7.23 (s, 1 H, Ar-**H**); **^{13}C-NMR** (75 MHz, $CDCl_3$, 298.0 K): δ / ppm = 27.7 (3 C, -**C**H$_3$), 43.3 (1 C, -**C**H$_2$), 56.2 (1 C, -O**C**H$_3$), 56.4 (1 C, -O**C**H$_3$), 69.3 (1 C, **C$_q$**), 84.2 (1 C, **C$_q$**), 105.7 (1 C, **C$_{Ar}$**), 107.0 (1 C, **C$_{Ar}$**), 125.5 (1 C, **C$_{Ar}$**), 146.5 (1 C, **C$_{Ar}$**), 150.2 (1 C, **C$_{Ar}$**), 156.8 (1 C, **C$_{Ar}$**), 166.1 (1 C, -**C**=O), 194.1 (1 C, -**C**=O); **MS** (ESI) *m*/*z*: calcd for $[C_{16}H_{19}ClO_5 + NH_4]^+$: 344.13; found: 344.35, **HPLC:** Chiralpak AD-H, *n*-hexane/*i*-PrOH 10:1, 0.5 mL/min, 10 °C; t_R = 26.5 min [major], 30.5 min [minor].

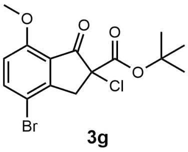

3g

3g was obtained as a yellowish oil after silica gel column chromatography using DCM/heptanes (5:1). ***e.r.*** = 77:23 (conditions A), 86:14 (conditions B); TLC (DCM/heptanes = 5/1): R_f = 0.31 (UV). $[\alpha]_D^{23}$ = -21.8 (*c* 1, $CHCl_3$, 77:23 *e.r.*, A), -23.9 (*c* 1, $CHCl_3$, 86:14 *e.r.*, B); **^{1}H-NMR** (300 MHz, $CDCl_3$, 298.0 K): δ / ppm = 1.45 (s, 9 H, -**CH$_3$**), 3.39 (d, J = 18.3 Hz, 1 H, -**CH$_2$**), 3.85 (d, J = 18.4 Hz, 1 H, -**CH$_2$**), 3.96 (s, 3 H, -OC**H$_3$**), 6.81 (d, J = 8.8 Hz, 1 H, Ar-**H**), 7.73 (d, J = 8.8 Hz, 1 H, Ar-**H**); **^{13}C-NMR** (75 MHz, $CDCl_3$, 298.0 K): δ / ppm = 27.7 (3 C, -**C**H$_3$), 44.1 (1 C, -**C**H$_2$), 56.3 (1 C, -O**C**H$_3$), 68.6 (1 C, **C$_q$**), 84.6 (1 C, **C$_q$**), 110.8 (1 C, **C$_{Ar}$**), 112.2 (1 C, **C$_{Ar}$**), 122.6 (1 C, **C$_{Ar}$**), 140.2 (1 C, **C$_{Ar}$**), 151.9 (1 C, **C$_{Ar}$**), 158.7 (1 C, **C$_{Ar}$**), 165.6 (1 C, -**C**=O), 192.4 (1 C, -**C**=O); **HRMS** (ESI) *m*/*z*: calcd for $[C_{15}H_{16}BrClO_4 + NH_4]^+$: 392.0258; found: 392.0266, **HPLC:** Chiralcel OD-H, *n*-hexane/*i*-PrOH 10:1, 0.5 mL/min, 10 °C; t_R = 18.4 min [major], 24.8 min [minor].

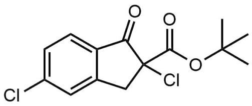

3h

3h was obtained as a yellowish white solid after silica gel column chromatography using DCM/heptanes (5:1). ***e.r.*** = 89:11 (conditions A), 94:6 (conditions B); TLC (DCM/heptanes = 5/1): R_f = 0.50 (UV). Analytical data are in accordance with those reported in literature[3]. $[\alpha]_D^{23}$ = -16.1 (*c* 1, $CHCl_3$, 89:11 *e.r.*, A), -21.9 (*c* 1, $CHCl_3$, 94:6 *e.r.*, B); **^{1}H-NMR** (300 MHz, $CDCl_3$, 298.0 K): δ / ppm = 1.43 (s, 9 H, -**CH$_3$**), 3.50 (d, J = 17.9 Hz, 1 H, -**CH$_2$**), 3.99 (d, J = 17.9 Hz, 1 H, -**CH$_2$**), 7.43-7.47 (m, 2 H, Ar-**H**), 7.78 (d, J = 8.2 Hz, 1 H, Ar-**H**); **^{13}C-NMR** (75 MHz, $CDCl_3$, 298.0 K): δ / ppm = 27.7 (3 C, -**C**H$_3$), 43.1 (1 C, -**C**H$_2$), 68.6 (1 C. **C$_q$**), 84.7 (1 C, **C$_q$**), 126.5 (1 C, **C$_{Ar}$**), 126.8 (1 C, **C$_{Ar}$**), 129.3 (1 C, **C$_{Ar}$**), 131.2 (1 C, **C$_{Ar}$**), 142.9 (1 C, **C$_{Ar}$**), 152.0 (1 C, **C$_{Ar}$**), 165.5 (1 C, -**C**=O), 194.1 (1 C, -**C**=O); **MS** (ESI) *m*/*z*:

(4) J. Luo, W. Wu, L. Xu, Y. Meng, Y. Lu, *Tetrahedron Lett.*, **2013**, *54*, 2623–2626

calcd for $[C_{14}H_{14}Cl_2O_3 + NH_4]^+$: 318.07; found: 318.25, **HPLC:** Chiralcel OD-H, *n*-hexane/*i*-PrOH 250:1, 0.5 mL/min, 10 °C; t_R = 26.2 min [major], 28.9 min [minor].

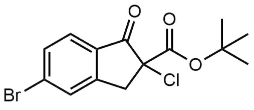

3i

3i was obtained as a yellowish white solid after silica gel column chromatography using DCM/heptanes (5:1). ***e.r.*** = 85:15 (conditions B); TLC (DCM/heptanes = 5/1): R_f = 0.49 (UV). Analytical data are in accordance with those reported in literature[3]. **$[\alpha]_D^{23}$** = -21.9 (*c* 1, $CHCl_3$, 85:15 *e.r.*, B); **^{1}H-NMR** (300 MHz, $CDCl_3$, 298.0 K): δ / ppm = 1.44 (s, 1 H, -**CH$_3$**), 3.54 (d, *J* = 18.0 Hz, 1 H, -**CH$_2$**), 4.01 (d, *J* = 18.0 Hz, 1 H, -**CH$_2$**), 7.34-7.40 (m, 1 H, Ar-**H**), 7.44-7.49 (m, 1 H, Ar-**H**), 7.67 (d, *J* = 7.5 Hz, 1 H, Ar-**H**); **^{13}C-NMR** (75 MHz, $CDCl_3$, 298.0 K): δ / ppm = 27.7 (3 C, **C**H$_3$), 43.1 (1 C, -**C**H$_2$), 68.5 (1 C, **C$_q$**), 84.7 (1 C, **C$_q$**), 126.9 (1 C, **C$_{Ar}$**), 129.6 (1 C, **C$_{Ar}$**), 131.7 (1 C, **C$_{Ar}$**), 131.8 (1 C, **C$_{Ar}$**), 132.2 (1 C, **C$_{Ar}$**), 152.1 (1 C, **C$_{Ar}$**), 165.5 (1 C, -**C**=O), 194.4 (1 C, -**C**=O); **MS** (ESI) *m*/*z*: calcd for $[C_{14}H_{14}BrClO_3 + NH_4]^+$: 362.02; found: 362.20, **HPLC:** Chiralcel OD-H, *n*-hexane/*i*-PrOH 250:1, 0.5 mL/min, 10 °C; t_R = 28.0 min [major], 31.6 min [minor].

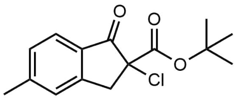

3j

3j was obtained as a yellow solid after silica gel column chromatography using DCM/heptanes (5:1). ***e.r.*** = 13:87 (conditions A), 9:91 (conditions B); TLC (DCM/heptanes = 5/1): R_f = 0.50 (UV). **$[\alpha]_D^{23}$** = -30.5 (*c* 1, $CHCl_3$, 13:87 *e.r.*, A), -33.6 (*c* 1, $CHCl_3$, 9:91 *e.r.*, B); **^{1}H-NMR** (300 MHz, $CDCl_3$, 298.0 K): δ / ppm = 1.42 (s, 9 H, -**CH$_3$**), 2.47 (s, 3 H, -**CH$_3$**), 3.47 (d, *J* = 17.7 Hz, 1 H, -**CH$_2$**), 3.96 (d, *J* = 17.7 Hz, 1 H, -**CH$_2$**), 7.24-7.26 (m, 2 H, Ar-**H**), 7.73 (d, *J* = 8.2 Hz, 1H, Ar-**H**); **^{13}C-NMR** (75 MHz, $CDCl_3$, 298.0 K): δ / ppm = 22.2 (1 C, -**C**H$_3$), 27.7 (3 C, -**C**H$_3$), 43.3 (1 C, -**C**H$_2$), 69.1 (1 C, **C$_q$**), 84.2 (1 C, **C$_q$**), 125.6 (1 C, **C$_{Ar}$**), 126.5 (1 C, **C$_{Ar}$**), 129.7 (1 C, **C$_{Ar}$**), 130.5 (1 C, **C$_{Ar}$**), 147.8 (1 C, **C$_{Ar}$**), 151.1 (1 C, **C$_{Ar}$**), 166.0 (1 C, -**C**=O), 195.0 (1 C, -**C**=O); **HRMS** (ESI) *m*/*z*: calcd for $[C_{15}H_{17}ClO_3 + NH_4]^+$: 298.1204; found: 298.1213, **HPLC:** Chiralpak AD-H, *n*-hexane/*i*-PrOH 10:1, 0.5 mL/min, 10 °C; t_R = 13.8 min [minor], 14.7 min [major].

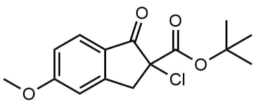

3k

3k was obtained as a white solid after silica gel column chromatography using DCM/heptanes (5:1). ***e.r.*** = 12:88 (conditions A), 10:90 (conditions B); TLC (DCM/heptanes = 5/1): R_f = 0.53 (UV). Analytical data are in accordance with those reported in literature[4]. **$[\alpha]_D^{23}$** = -23.4 (*c* 1, $CHCl_3$, 12:88 *e.r.*, A), -23.9 (*c* 1, $CHCl_3$, 10:90 *e.r.*, B); **^{1}H-NMR** (300 MHz, $CDCl_3$, 298.0 K): δ / ppm = 1.43 (s, 9 H, -**CH$_3$**), 3.47 (d, *J* = 17.7 Hz, 1 H, -**CH$_2$**), 3.91 (s, 3 H, -OC**H$_3$**), 3.97 (d, *J* = 17.8 Hz, 1 H, -**CH$_2$**), 6.88 (s, 1 H, Ar-**H**), 6.97 (dd, J_1 = 2.1 Hz, J_2 = 8.6 Hz, 1 H, Ar-**H**), 7.78 (d, *J* = 8.6 Hz, 1 H, Ar-**H**); **^{13}C-NMR** (75 MHz, $CDCl_3$, 298.0 K): δ / ppm = 27.7 (3 C,-**C**H$_3$), 43.5 (1 C, -**C**H$_2$), 55.8 (1 C, -O**C**H$_3$), 69.2 (1 C, **C$_q$**), 84.2 (1 C, **C$_q$**), 109.4 (1 C, **C$_{Ar}$**), 116.5 (1 C, **C$_{Ar}$**), 125.8 (1 C, **C$_{Ar}$**), 127.6 (1 C, **C$_{Ar}$**), 153.8 (1 C, **C$_{Ar}$**), 166.1 (1 C, **C$_{Ar}$**), 166.5 (1 C, -**C**=O), 193.6 (1 C, -**C**=O); **MS** (ESI) *m*/*z*: calcd for $[C_{15}H_{17}ClO_4 + NH_4]^+$:

314.12; found: 314.30, **HPLC:** Chiralpak AD-H, *n*-hexane/*i*-PrOH 10:1, 0.5 mL/min, 10 °C; t_R = 20.3 min [minor], 21.1 min [major].

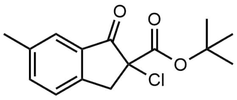

3l

3l was obtained as a colourless oil after silica gel column chromatography using DCM/heptanes (5:1). ***e.r.*** = 61:39 (conditions A), 91:9 (conditions B); TLC (DCM/heptanes = 5/1): R_f = 0.52 (UV). Analytical data are in accordance with those reported in literature[4]. $[\alpha]_D^{23}$ = -7.4 (*c* 1, $CHCl_3$, 61:39 *e.r.,* A), -18.2 (*c* 1, $CHCl_3$, 91:9 *e.r.*, B); **^{1}H-NMR** (300 MHz, $CDCl_3$, 298.0 K): δ / ppm = 1.42 (s, 9 H, -C**H**$_3$), 2.42 (s, 3 H, -C**H**$_3$), 3.47 (d, *J* = 17.9 Hz, 1 H, -C**H**$_2$), 3.95 (d, *J* = 17.9 Hz, 1 H, -C**H**$_2$), 7.35 (d, *J* = 7.8 Hz, 1 H, Ar-**H**), 7.50 (d, *J* = 7.9 Hz, 1 H, Ar-**H**), 7.64 (s, 1 H, Ar-**H**); **^{13}C-NMR** (75 MHz, $CDCl_3$, 298.0 K): δ / ppm = 21.1 (1 C, -**C**H$_3$), 27.7 (3 C, -**C**H$_3$), 43.2 (1 C, -**C**H$_2$), 69.1 (1 C, **C**$_q$), 84.3 (1 C, **C**$_q$), 125.7 (1 C, **C**$_{Ar}$), 125.9 (1 C, **C**$_{Ar}$), 133.0 (1 C, **C**$_{Ar}$), 137.5 (1 C, **C**$_{Ar}$), 138.6 (1 C, **C**$_{Ar}$), 148.1 (1 C, **C**$_{Ar}$), 166.0 (1 C, -**C**=O), 195.6 (1 C, -**C**=O); **MS** (ESI) *m*/*z*: calcd for $[C_{15}H_{17}ClO_3 + NH_4]^+$: 298.12; found: 298.30, **HPLC:** Chiralcel OD-H, *n*-hexane/*i*-PrOH 250:1, 0.5 mL/min, 10 °C; t_R = 18.9 min [major], 21.0 min [minor].

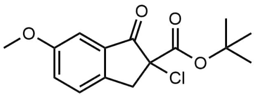

3m

3m was obtained as a yellowish white oil after silica gel column chromatography using DCM/heptanes (5:1). ***e.r.*** = 90:10 (conditions A), 93:7 (conditions B); TLC (DCM/heptanes = 5/1): R_f = 0.50 (UV). Analytical data are in accordance with those reported in literature[4]. $[\alpha]_D^{23}$ = -13.9 (*c* 1, $CHCl_3$, 90:10 *e.r.,* A), -17.8 (*c* 1, $CHCl_3$, 93:7 *e.r.*, B); **^{1}H-NMR** (300 MHz, $CDCl_3$, 298.0 K): δ / ppm = 1.43 (s, 9 H, -C**H**$_3$), 3.45 (d, *J* = 17.4 Hz, 1 H, -C**H**$_2$), 3.85 (s, 3 H, -OC**H**$_3$), 3.92 (d, *J* = 17.4 Hz, 1 H, -C**H**$_3$), 7.25-7.28 (m, 2 H, Ar-**H**), 7.35 (d, *J* = 9.3 Hz, 1 H, Ar-**H**); **^{13}C-NMR** (75 MHz, $CDCl_3$, 298.0 K): δ / ppm = 27.7 (3 C, -**C**H$_3$), 42.9 (1 C, -**C**H$_2$), 55.7 (1 C, -OCH$_3$), 69.4 (1 C, **C**$_q$), 84.3 (1 C, **C**$_q$), 106.6 (1 C, **C**$_{Ar}$), 125.7 (1 C, **C**$_{Ar}$), 126.9 (1 C, **C**$_{Ar}$), 134.0 (1 C, **C**$_{Ar}$), 143.5 (1 C, **C**$_{Ar}$), 160.1 (1 C, **C**$_{Ar}$), 165.9 (1 C, -**C**=O), 195.6 (1 C, -**C**=O); **MS** (ESI) *m*/*z*: calcd for $[C_{15}H_{17}ClO_4 + NH_4]^+$: 314.12; found: 314.30, **HPLC:** Chiralcel OD-H, *n*-hexane/*i*-PrOH 250:1, 0.5 mL/min, 10 °C; t_R = 34.7 min [major], 38.5 min [minor].

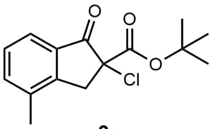

3n

3n was obtained as a slightly yellow solid after silica gel column chromatography using DCM/heptanes (5:1). ***e.r.*** = 87:13 (conditions A), 93:7 (conditions B); TLC (DCM/heptanes = 5/1): R_f = 0.50 (UV). Analytical data are in accordance with those reported in literature[3]. $[\alpha]_D^{23}$ = -24.7 (*c* 1, $CHCl_3$, 87:13 *e.r.,* A), -25.7 (*c* 1, $CHCl_3$, 93:7 *e.r.*, B); **^{1}H-NMR** (300 MHz, $CDCl_3$, 298.0 K): δ / ppm = 1.44 (s, 9 H, -C**H**$_3$), 2.35 (s, 3 H, -C**H**$_3$), 3.42 (d, *J* = 17.8 Hz, 1 H, -C**H**$_2$), 3.90 (d, *J* = 17.8 Hz, 1 H, -C**H**$_2$), 7.37 (t, *J* = 7.5 Hz, 1 H, Ar-**H**), 7.49 (d, *J* = 7.3 Hz, 1 H, Ar-**H**), 7.69 (d, *J* = 7.6 Hz, 1 H, Ar-**H**); **^{13}C-NMR** (75 MHz, $CDCl_3$, 298.0 K): δ / ppm = 17.7 (1 C, -**C**H$_3$), 27.7 (3 C, -**C**H$_3$), 42.5 (1 C, -**C**H$_2$), 68.6 (1 C, **C**$_q$), 84.4 (1 C, **C**$_q$), 123.2

(1 C, **C**$_{Ar}$), 128.6 (1 C, **C**$_{Ar}$), 132.5 (1 C, **C**$_{Ar}$), 135.5 (1 C, **C**$_{Ar}$), 136.7 (1 C, **C**$_{Ar}$), 149.5 (1 C, **C**$_{Ar}$), 166.0 (1 C, -**C**=O), 195.8 (1 C, -**C**=O); **MS** (ESI) *m*/*z*: calcd for [$C_{15}H_{17}ClO_3 + NH_4$]$^+$: 298.12; found: 298.30, **HPLC:** Chiralcel OD-H, *n*-hexane/*i*-PrOH 250:1, 0.5 mL/min, 10 °C; t_R = 23.5 min [major], 26.0 min [minor].

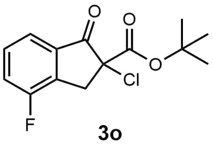

3o

3o was obtained as white needles after silica gel column chromatography using DCM/heptanes (5:1). ***e.r.*** = 84:16 (conditions A), 89:11 (conditions B); TLC (DCM/heptanes = 5/1): R_f = 0.24 (UV). $[\alpha]_D^{23}$ = -17.9 (*c* 1, $CHCl_3$, 84:16 *e.r.*, A), -28.2 (*c* 1, $CHCl_3$, 89:11 *e.r.*, B); **^{1}H-NMR** (300 MHz, $CDCl_3$, 298.0 K): δ / ppm = 1.39 (s, 9 H, -**CH$_3$**), 3.48 (d, *J* = 18.1 Hz, 1 H, -**CH$_2$**), 3.96 (d, *J* = 18.1 Hz, 1 H, -**CH$_2$**), 7.29-7.34 (m, 1 H, Ar-**H**), 7.38-7.45 (m, 1 H, Ar-**H**), 7.62 (d, *J* = 7.5 Hz, 1 H, Ar-**H**); **^{13}C-NMR** (75 MHz, $CDCl_3$, 298.0 K): δ / ppm = 27.7 (3 C, -**C**H$_3$), 43.1 (1 C, -**C**H$_2$), 68.6 (1 C, **C**$_q$), 84.7 (1 C, **C**$_q$), 121.5 (d, *J* = 4.1 Hz, 1 C, **C**$_{Ar}$), 122.3 (d, *J* = 19.8 Hz, 1 C, **C**$_{Ar}$), 130.5 (d, *J* = 6.1 Hz, 1 C, **C**$_{Ar}$), 135.4 (d, *J* = 4.4 Hz, 1 C, **C**$_{Ar}$), 136.6 (d, *J* = 19.7 Hz, 1 C, **C**$_{Ar}$), 159.4 (d, *J* = 251.5 Hz, 1 C, **C**$_{Ar}$), 165.4 (1 C, -**C**=O), 194.5 (1 C, -**C**=O); **HRMS** (ESI) *m*/*z*: calcd for [$C_{14}H_{14}ClFO_3 + NH_4$]$^+$: 302.0954; found: 302.0963, **HPLC:** Chiralcel OD-H, *n*-hexane/*i*-PrOH 250:1, 0.5 mL/min, 10 °C; t_R = 19.9 min [major], 21.2 min [minor].

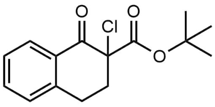

3p

3p was obtained as a slightly brown oil after silica gel column chromatography using DCM/heptanes (5:1). ***e.r.*** = 54:46 (conditions A), 66:34 (conditions B); TLC (DCM/heptanes = 5/1): R_f = 0.50 (UV). Analytical data are in accordance with those reported in literature[5]. $[\alpha]_D^{23}$ = -4.0 (*c* 1, $CHCl_3$, 54:46 *e.r.*, A), -6.4 (*c* 1, $CHCl_3$, 66:34 *e.r.*, B); **^{1}H-NMR** (300 MHz, $CDCl_3$, 298.0 K): δ / ppm = 1.46 (s, 9 H, -**CH$_3$**), 2.47-2.55 (m, 1 H, -**CH$_2$**), 2.90-3.06 (m, 2 H, -**CH$_2$**), 3.20-3.30 (m, 1 H, -**CH$_2$**), 7.25-7.28 (m, 1 H, Ar-**H**), 7.36 (t, *J* = 7.4 Hz, 1 H, Ar-**H**), 7.53 (td, J_1 = 1.4 Hz, J_2 = 7.6 Hz, 1 H, Ar-**H**), 8.09 (dd, J_1 = 1.1 Hz, J_2 = 7.9 Hz, 1 H, Ar-**H**); **^{13}C-NMR** (75 MHz, $CDCl_3$, 298.0 K): δ / ppm = 25.9 (1 C, -**C**H$_2$), 27.7 (3 C, -**C**H$_3$), 35.2 (1 C, -**C**H$_2$), 71.6 (1 C, **C**$_q$), 84.1 (1 C, **C**$_q$), 127.2 (1 C, **C**$_{Ar}$), 128.7 (1 C, **C**$_{Ar}$), 128.8 (1 C, **C**$_{Ar}$), 130.1 (1 C, **C**$_{Ar}$), 134.1 (1 C, **C**$_{Ar}$), 142.4 (1 C, **C**$_{Ar}$), 166.2 (1 C, -**C**=O), 188.0 (1 C, -**C**=O); **MS** (ESI) *m*/*z*: calcd for [$C_{15}H_{17}ClO_3 + NH_4$]$^+$: 298.12; found: 298.30, **HPLC:** Chiralcel OJ-H, *n*-hexane/*i*-PrOH 10:1, 0.5 mL/min, 10 °C; t_R = 16.4 min [major], 17.7 min [minor].

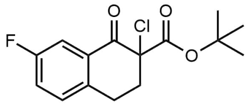

3q

3q was obtained as slightly brown oil after silica gel column chromatography using DCM/heptanes (5:1). ***e.r.*** = 66:34 (conditions A), 71:29 (conditions B); TLC (DCM/heptanes = 5/1): R_f = 0.24 (UV). $[\alpha]_D^{23}$ = -17.9 (*c* 1, $CHCl_3$, 66:34 *e.r.*, A), -28.2 (*c* 1, $CHCl_3$, 71:29 *e.r.*, B); **^{1}H-NMR** (300 MHz, $CDCl_3$, 298.0 K): δ / ppm = 1.46 (s, 9 H, -**CH$_3$**), 2.46-2.55 (m, 1 H, -**CH$_2$**), 2.88-3.03 (m, 2 H, -**CH$_2$**), 3.15-3.25 (m, 1 H, -**CH$_2$**), 7.23-7.26 (m, 2 H, Ar-**H**), 7.72-7.76 (m, 1 H, Ar-**H**); **^{13}C-NMR** (75 MHz, $CDCl_3$, 298.0

(5) Y. Cai, W. Wang, K. Shen, J. Wang, X. Hu, L. Lin, X. Liu, X. Feng, *Chem. Commun.*, **2010**, *46,* 1250–1252

K): *δ* / ppm = 25.3 (1 C, -**C**H_2), 27.7 (3 C, -**C**H_3), 35.3 (1 C, -**C**H_2), 71.1 (1 C, $\mathbf{C_q}$), 84.4 (1 C, **Cq**), 114.5 (d, *J* = 22.3 Hz, 1 C, $\mathbf{C_{Ar}}$), 121.7 (d, *J* = 22.2 Hz, 1 C, $\mathbf{C_{Ar}}$), 130.6 (7.2 Hz, 1 C, $\mathbf{C_{Ar}}$), 131.7 (d, *J* = 7.7 Hz, 1 C, $\mathbf{C_{Ar}}$), 138.1 (d, *J* = 3.0 Hz, 1 C, $\mathbf{C_{Ar}}$), 161.7 (d, *J* = 247.9 Hz, 1 C, $\mathbf{C_{Ar}}$), 165.9 (1 C, -**C**=O), 187.1 (d, *J* = 2.2 Hz, 1 C, -**C**=O); **HRMS** (ESI) *m*/*z*: calcd for $[C_{15}H_{16}ClFO_3 + H]^+$: 299.0845; found: 299.0850, **HPLC:** Chiralpak AD-H, *n*-hexane/*i*-PrOH 10:1, 0.5 mL/min, 10 °C; t_R = 10.0 min [major], 10.5 min [minor].

3. HRMS of Complex A

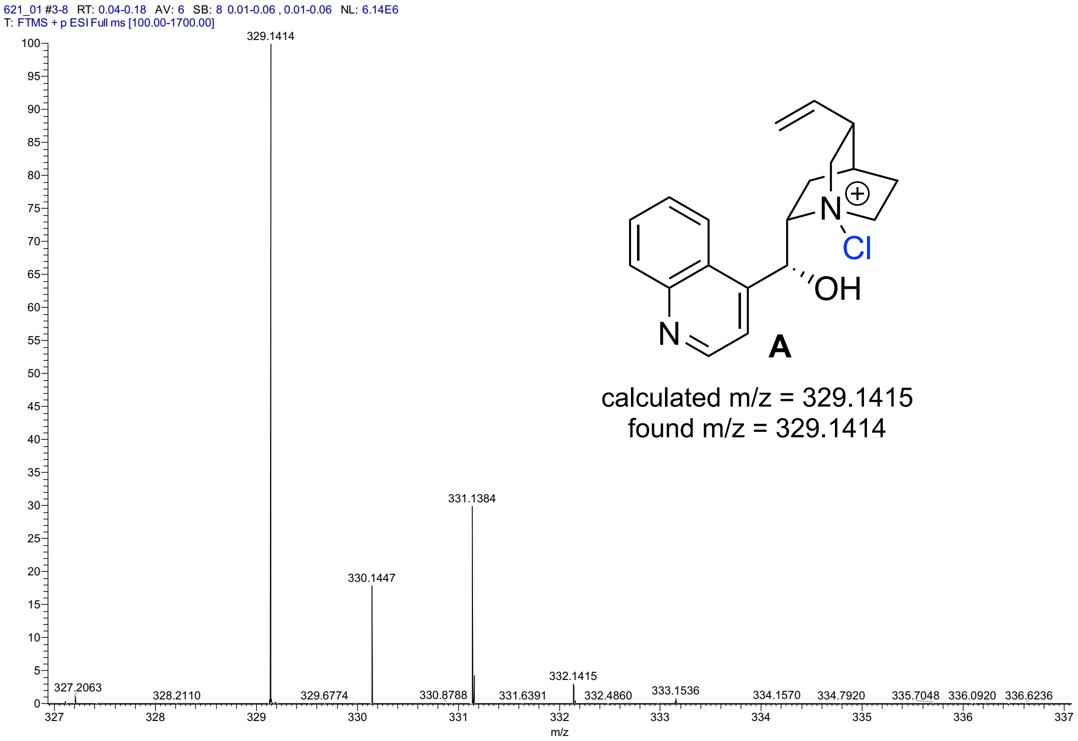

4. Computational details for Complex A

Calculations were performed using Gaussian 09[6]: Geometry optimisations were performed at the B3LYP/6-31+G(d) level of theory using LANL2DZ for iodine including an implicit description of toluene as solvent by the polarized continuum model as well as empirical dispersion as incorporated in Gaussian 09. Reoptimisation calculations were performed using B3LYP/6-311++G(d,p)/LANL2DZ, again including an implicit solvent description as well as dispersion. Gibbs free energies were obtained by vibrational frequency calculations using B3LYP/6-311++G(d,p)/LANL2DZ.

Cartesian coordinates and energies

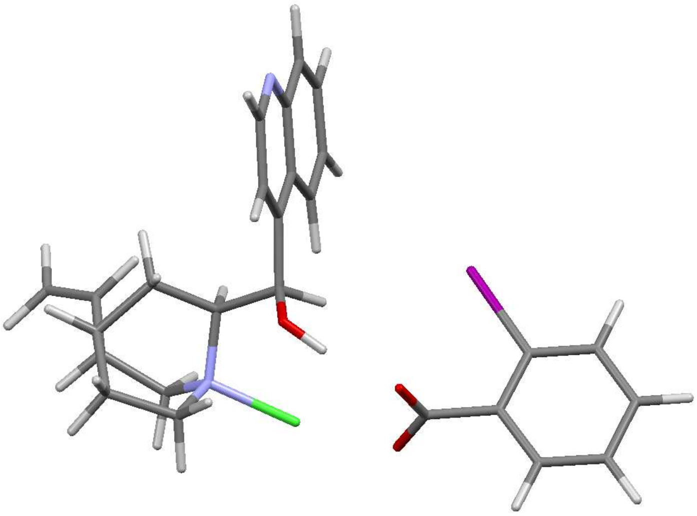

E_{el} (B3LYP/6-311++(d,p)/LANL2DZ) = -1813.596743
G_{tot} (B3LYP/6-311++(d,p)/LANL2DZ) = -1813.186983
# of imaginary frequencies: 0.

C	-2.22070	-0.71950	0.19310
C	-4.50400	-1.73100	0.46540
C	-3.59840	-0.55530	0.87150
H	-2.23210	-0.21000	-0.77000
H	-3.47810	-0.54090	1.95780
H	-4.02010	0.40490	0.57300
C	-2.47390	-3.10320	0.95930
H	-2.17940	-4.10780	0.65910
H	-1.89090	-2.79390	1.82090
C	-3.99830	-2.98220	1.19920
H	-4.51810	-3.87170	0.83320
H	-4.18860	-2.89500	2.27100
H	-5.54260	-1.52280	0.72550
C	-4.40070	-1.99690	-1.06010
H	-5.06880	-2.82770	-1.30250
C	-2.93300	-2.45800	-1.35990
H	-2.88000	-3.52870	-1.55650

(6) Gaussian 09, Revision C.01, M. J. Frisch, G. W. Trucks, H. B. Schlegel, G. E. Scuseria, M. A. Robb, J. R. Cheeseman, G. Scalmani, V. Barone, B. Mennucci, G. A. Petersson, H. Nakatsuji, M. Caricato, X. Li, H. P. Hratchian, A. F. Izmaylov, J. Bloino, G. Zheng, J. L. Sonnenberg, M. Hada, M. Ehara, K. Toyota, R. Fukuda, J. Hasegawa, M. Ishida, T. Nakajima, Y. Honda, O. Kitao, H. Nakai, T. Vreven, J. A. Montgomery, Jr., J. E. Peralta, F. Ogliaro, M. Bearpark, J. J. Heyd, E. Brothers, K. N. Kudin, V. N. Staroverov, T. Keith, R. Kobayashi, J. Normand, K. Raghavachari, A. Rendell, J. C. Burant, S. S. Iyengar, J. Tomasi, M. Cossi, N. Rega, J. M. Millam, M. Klene, J. E. Knox, J. B. Cross, V. Bakken, C. Adamo, J. Jaramillo, R. Gomperts, R. E. Stratmann, O. Yazyev, A. J. Austin, R. Cammi, C. Pomelli, J. W. Ochterski, R. L. Martin, K. Morokuma, V. G. Zakrzewski, G. A. Voth, P. Salvador, J. J. Dannenberg, S. Dapprich, A. D. Daniels, O. Farkas, J. B. Foresman, J. V. Ortiz, J. Cioslowski and D. J. Fox, Gaussian, Inc., Wallingford CT, 2010.

H	-2.50080	-1.92080	-2.20380
C	-4.79940	-0.80750	-1.88820
H	-4.18860	0.08680	-1.78220
C	-5.83770	-0.79300	-2.72020
H	-6.09350	0.09170	-3.29310
H	-6.46470	-1.67110	-2.85050
C	-1.03130	-0.11890	0.99030
H	-0.15420	-0.11670	0.34250
O	-0.74580	-0.87130	2.15050
H	0.15280	-1.24110	2.02060
C	-1.37040	1.32460	1.31270
C	-1.34900	2.32320	0.29420
C	-1.73360	1.69760	2.58370
C	-0.93720	2.08110	-1.04490
C	-1.74630	3.65040	0.65830
C	-2.10170	3.03920	2.83840
H	-1.72930	0.96920	3.38360
C	-0.93380	3.09410	-1.97620
H	-0.59440	1.09630	-1.33470
C	-1.74060	4.67070	-0.32820
H	-2.39380	3.32780	3.84540
C	-1.34540	4.39930	-1.61690
H	-0.60240	2.89390	-2.98870
H	-2.04760	5.66500	-0.02610
H	-1.33790	5.18760	-2.36100
N	-2.12610	3.98670	1.92580
N	-2.07250	-2.18990	-0.16810
Cl	-0.18190	-2.54960	-0.52860
C	5.50410	0.25040	-0.32550
C	4.17850	-0.15770	-0.18670
C	3.86150	-1.46510	0.18850
C	4.90510	-2.36790	0.41720
C	6.23330	-1.97010	0.29010
C	6.53250	-0.65970	-0.08060
H	5.73610	1.26610	-0.61970
H	4.66330	-3.38640	0.69960
H	7.03040	-2.68010	0.47800
H	7.56290	-0.33920	-0.18440
C	2.42750	-1.91690	0.37470
O	1.84450	-1.61040	1.42300
O	1.96730	-2.57590	-0.62630
I	2.63000	1.27410	-0.59480

5. NMR Spectra of New and Representative Known Chlorinated Products:

NMR spectra of **3a**:

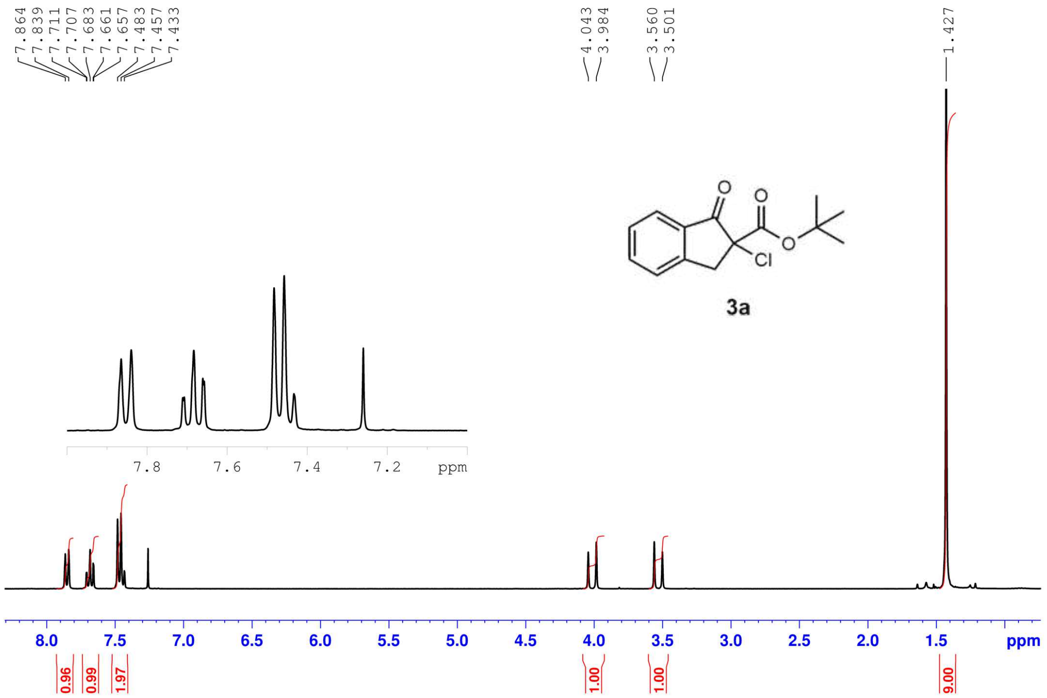

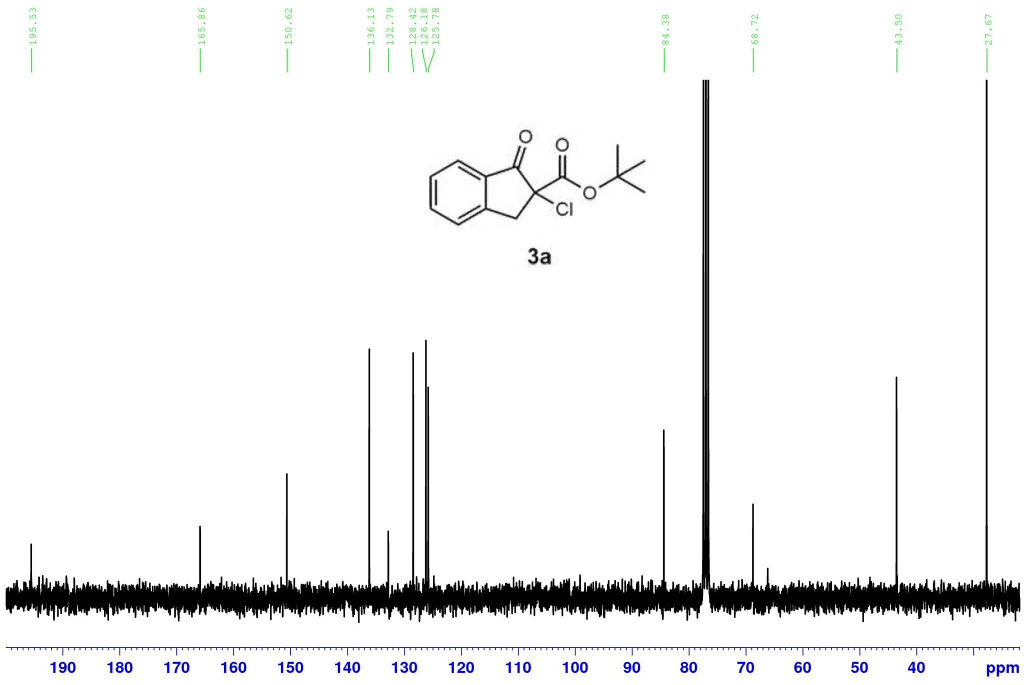

NMR spectra of **3b**:

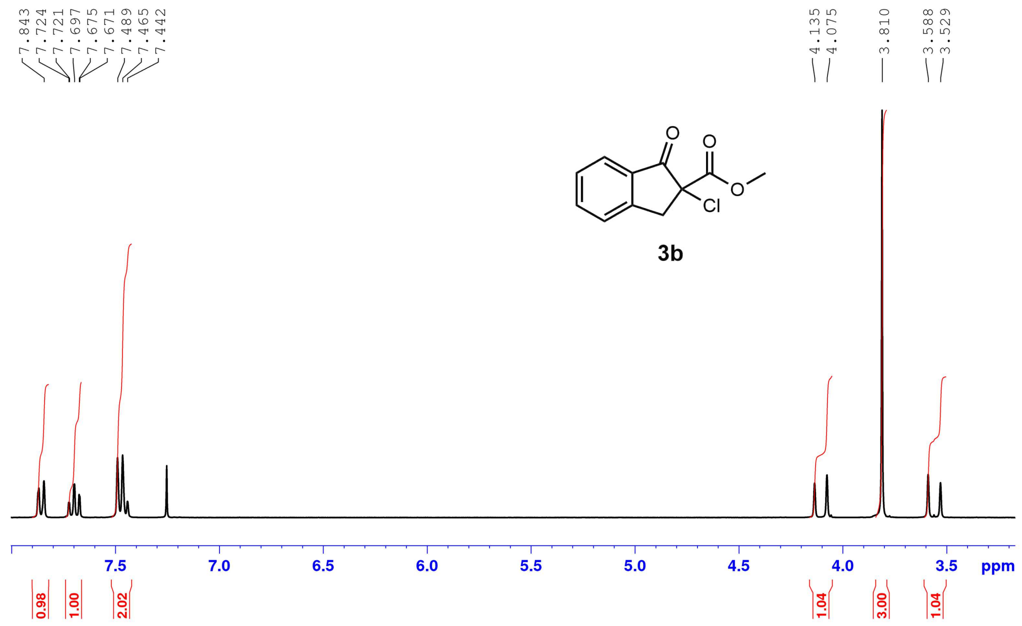

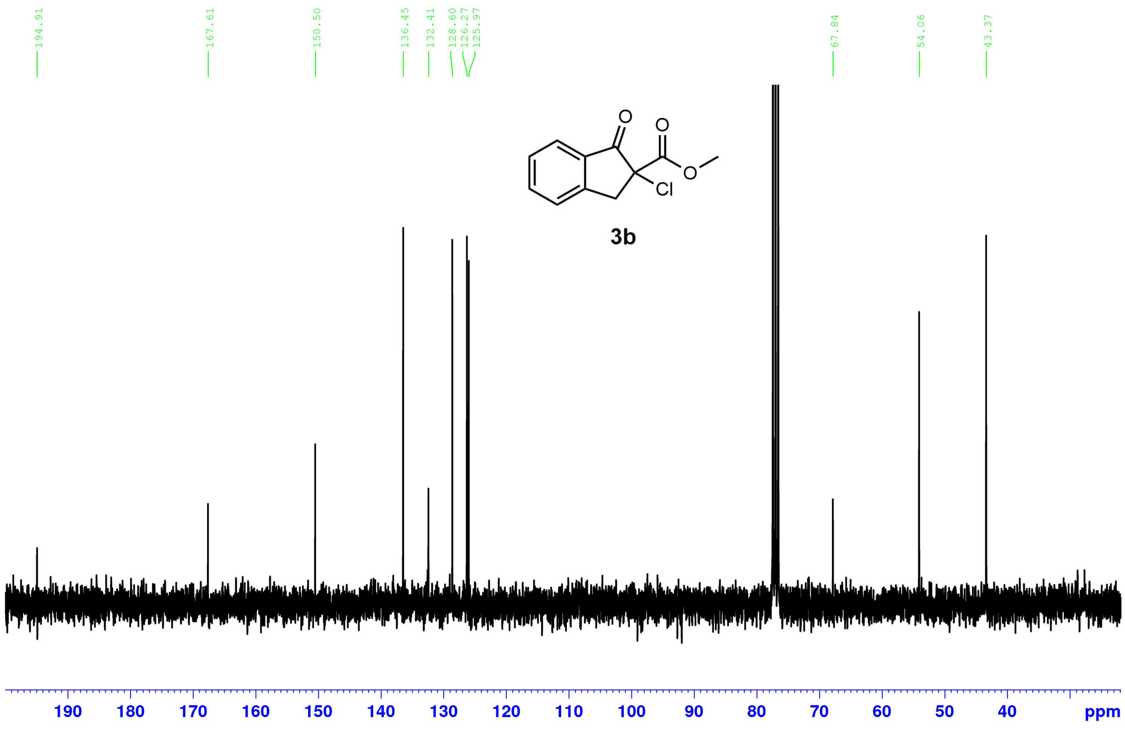

NMR spectra of **3c**:

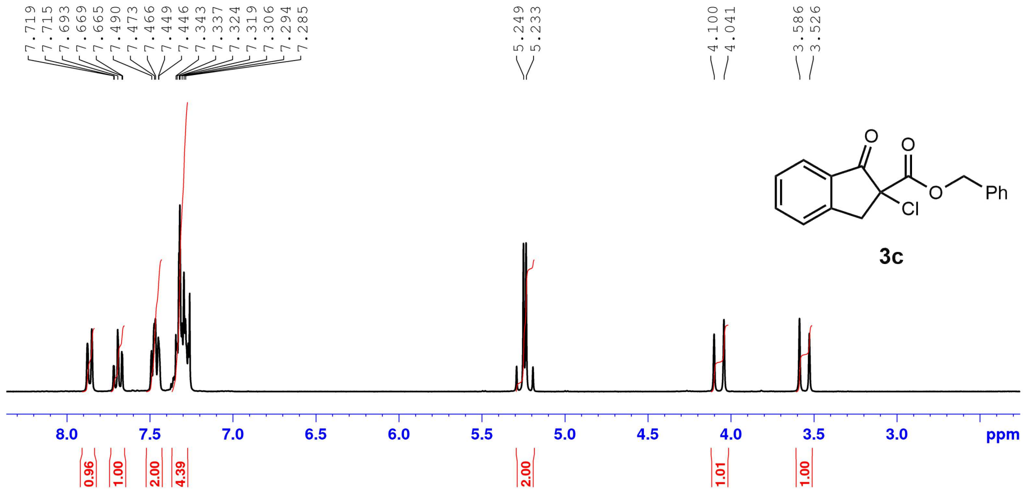

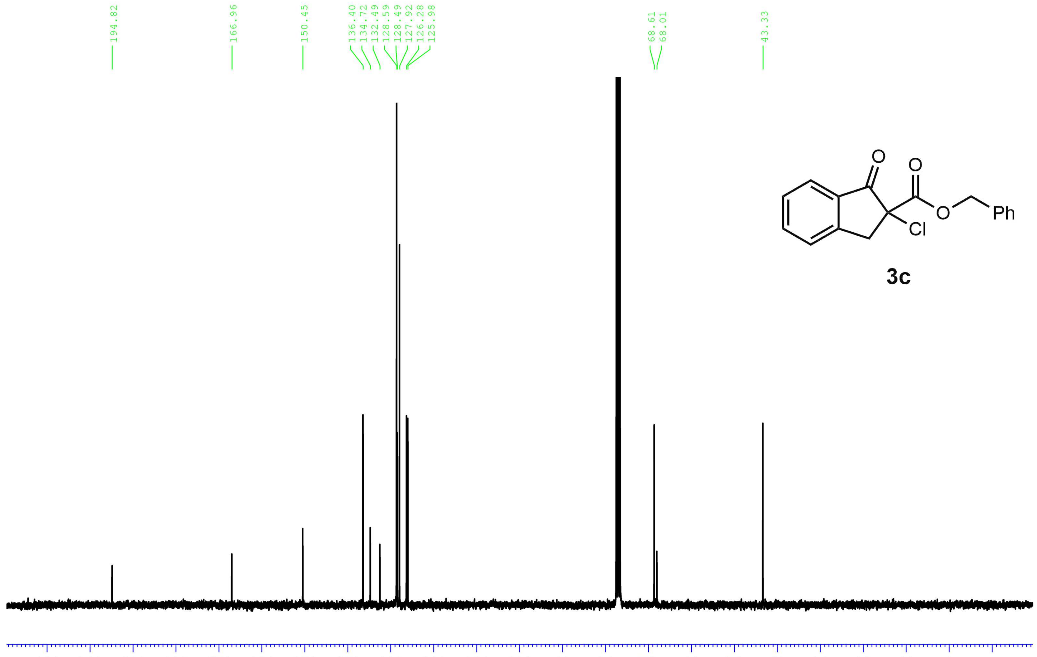

NMR spectra of **3e**:

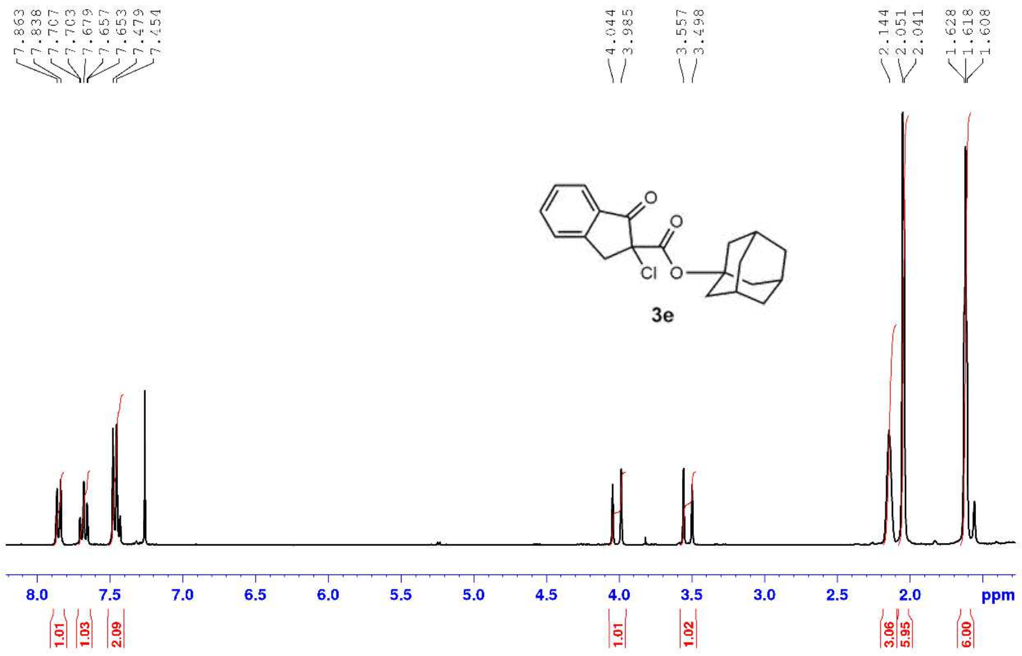

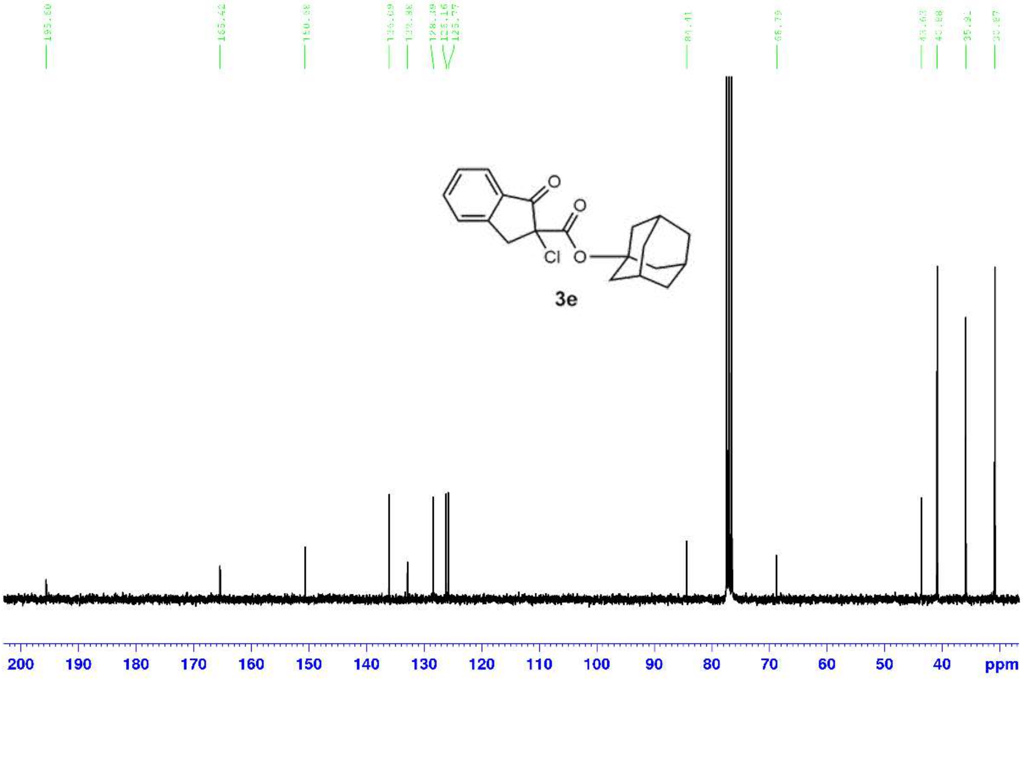

1H NMR spectrum of **3f**:

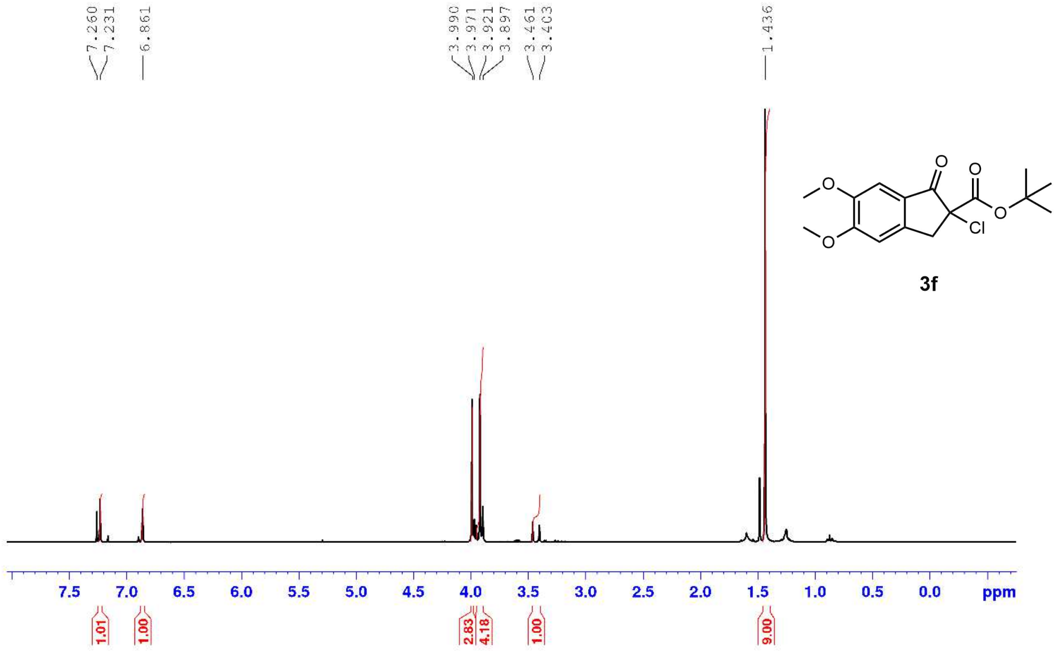

1H NMR spectrum of **3k**:

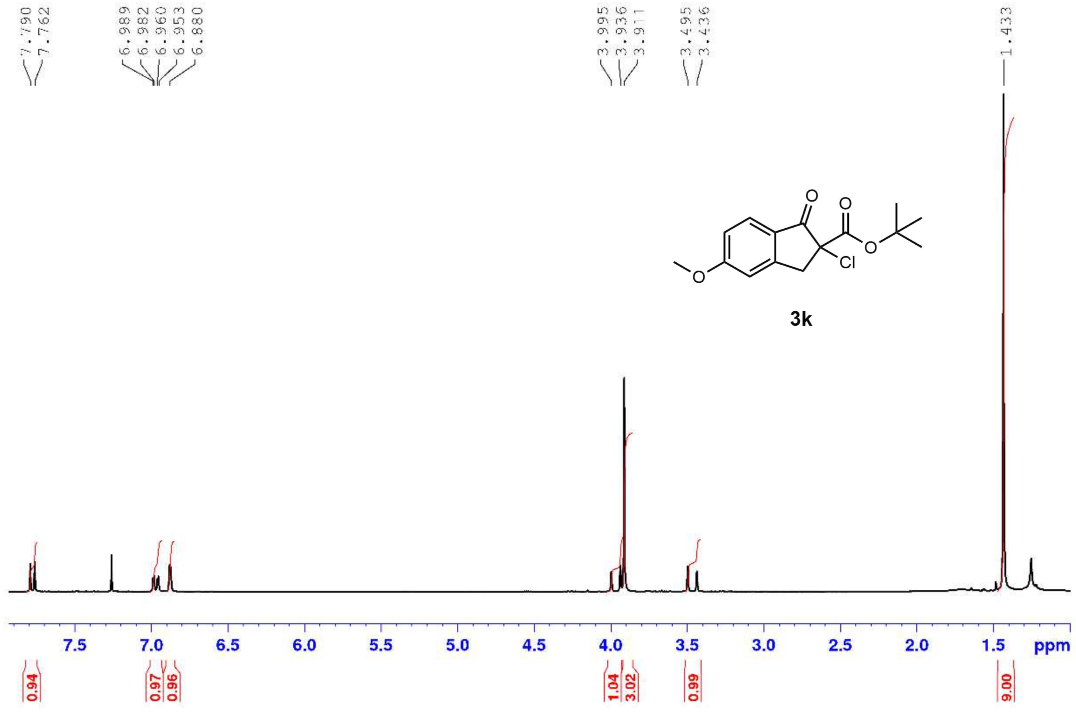

NMR spectra of **3g**:

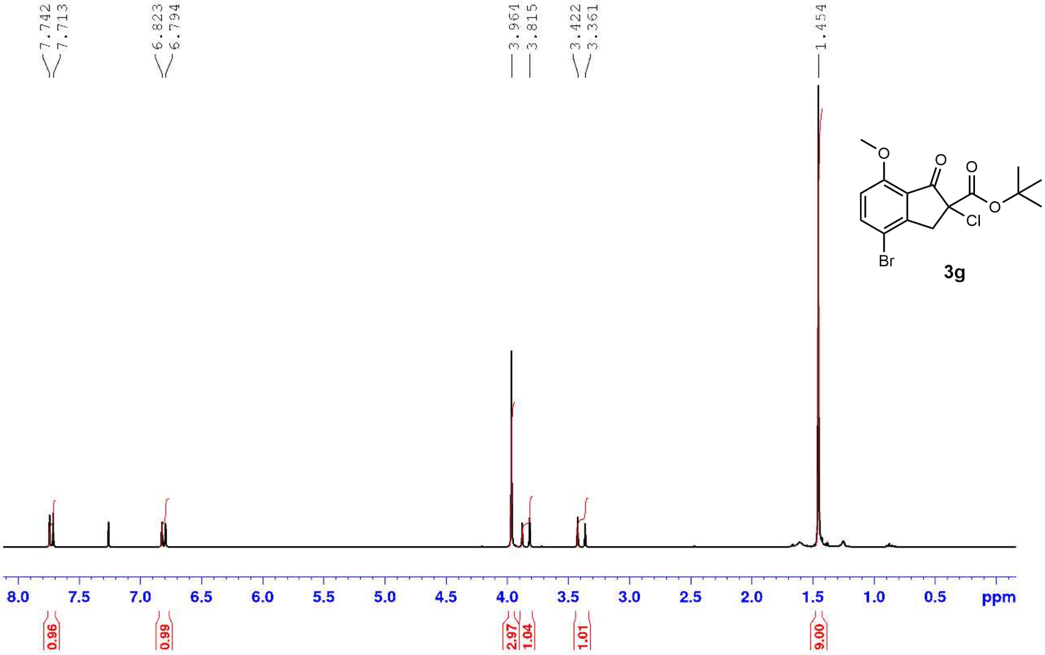

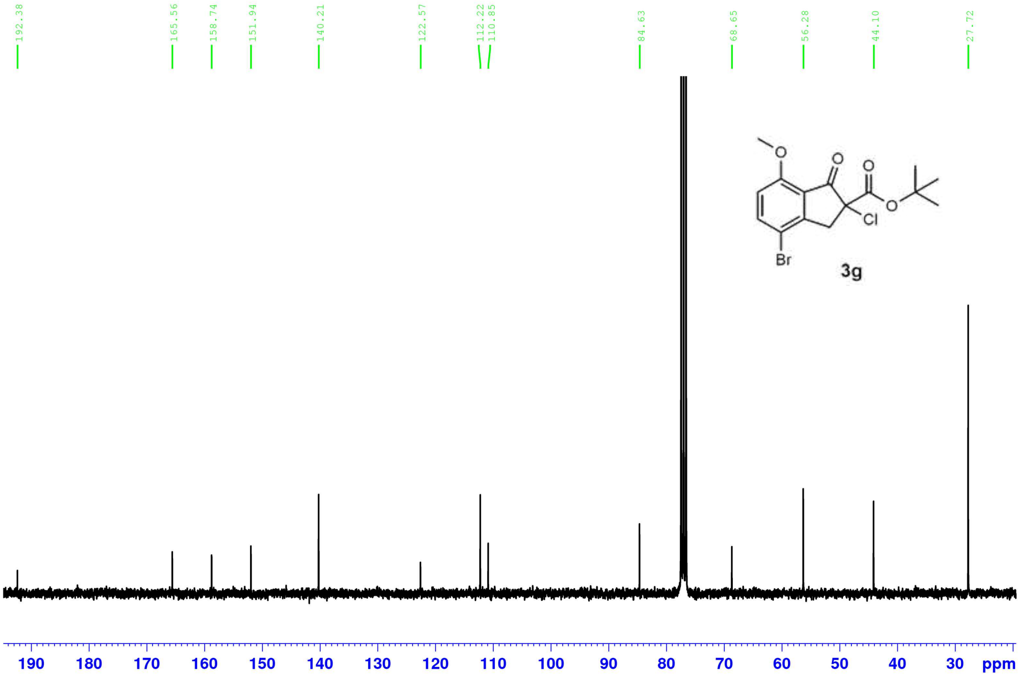

NMR spectra of **3h**:

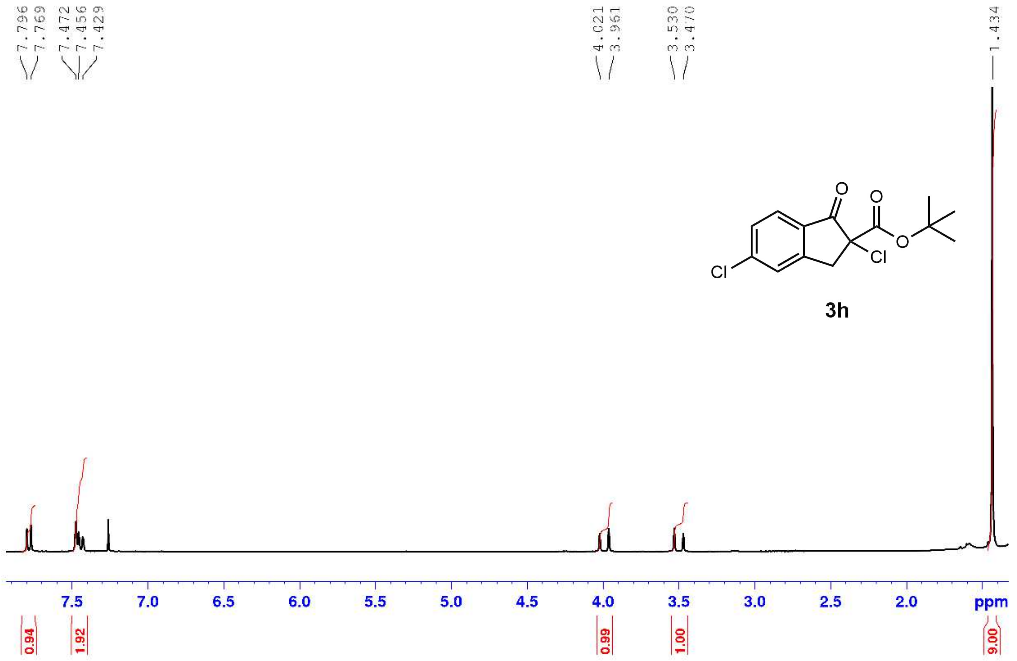

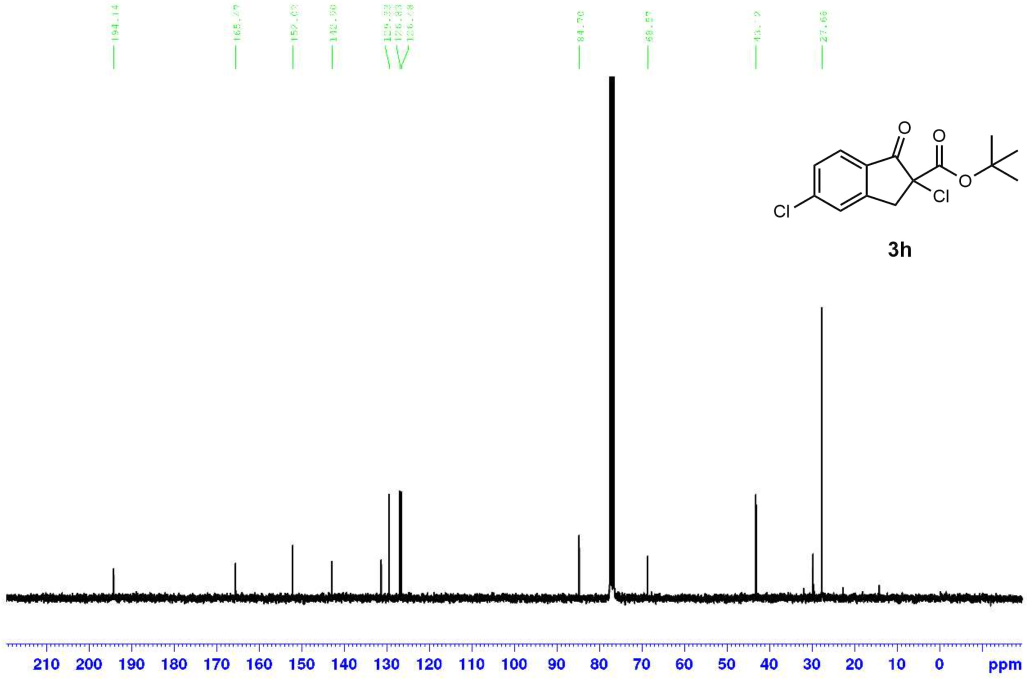

NMR spectra of **3i**:

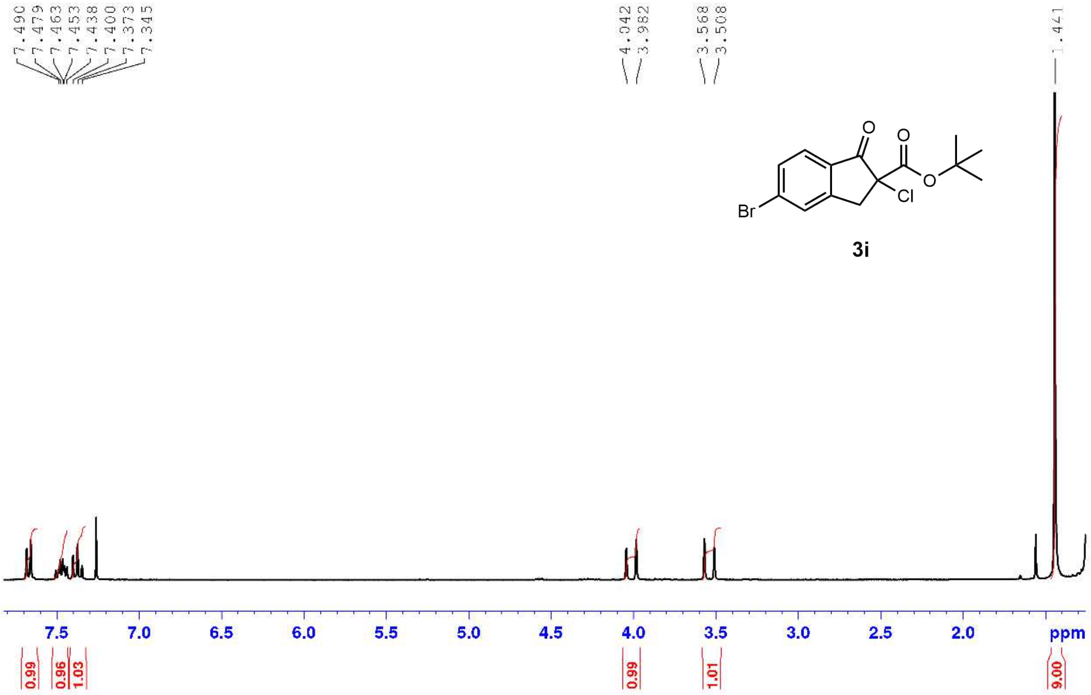

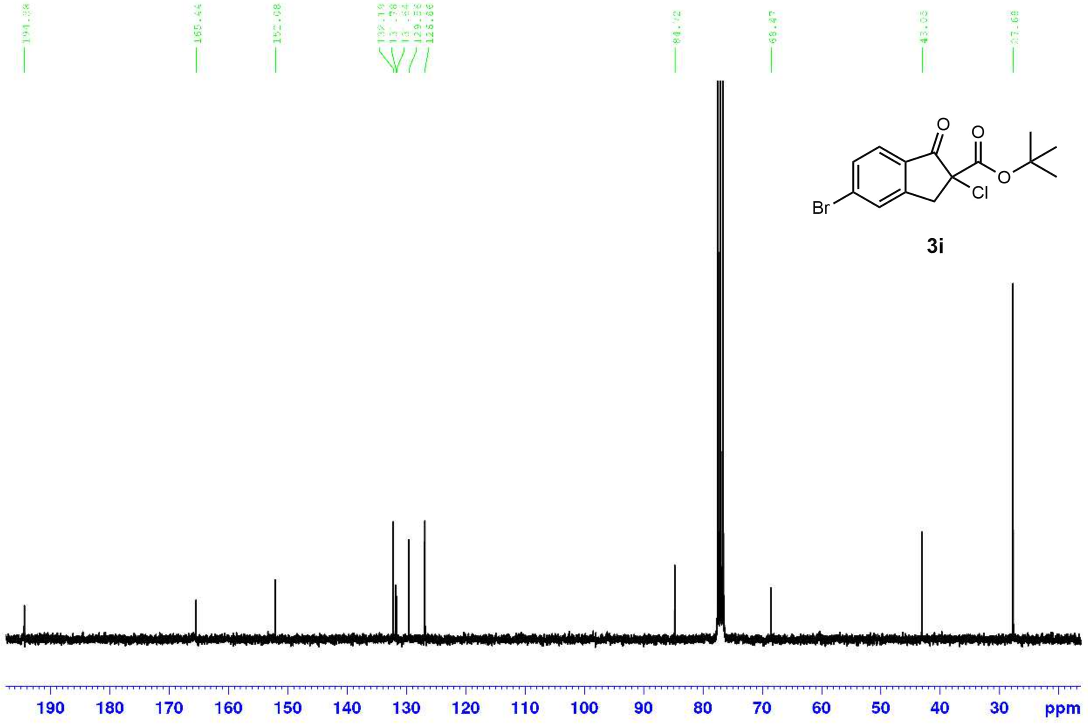

NMR spectra of **3j**:

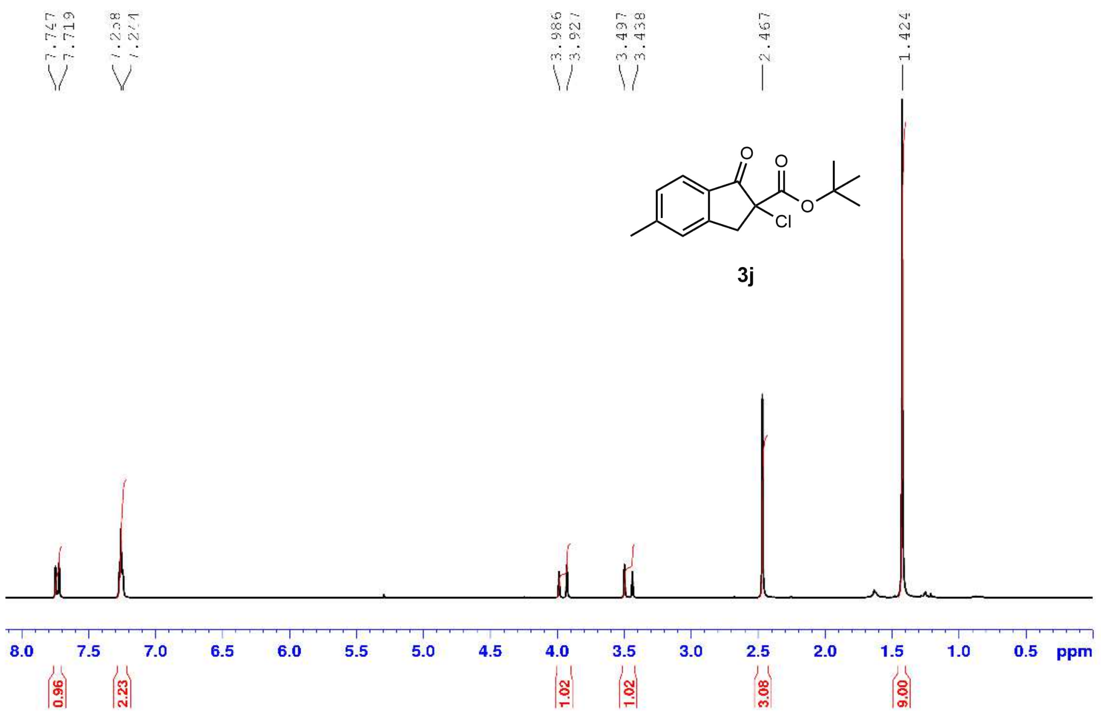

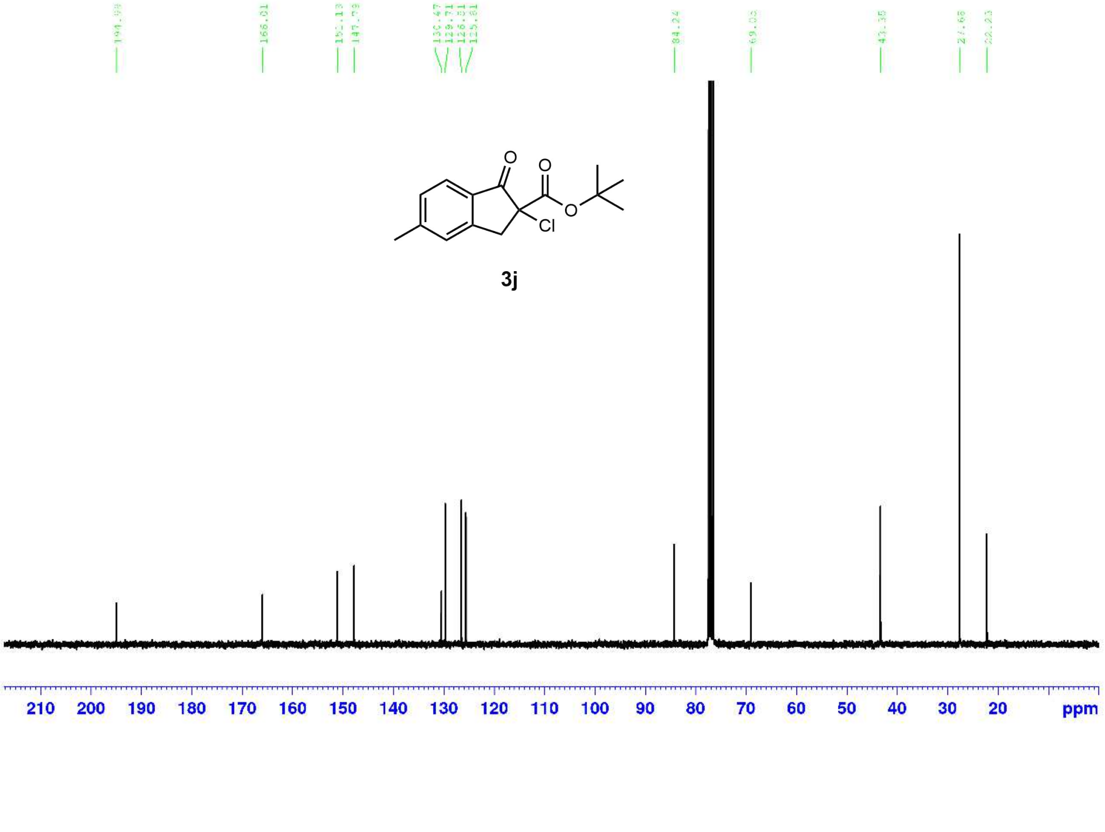

NMR spectra of **3l**:

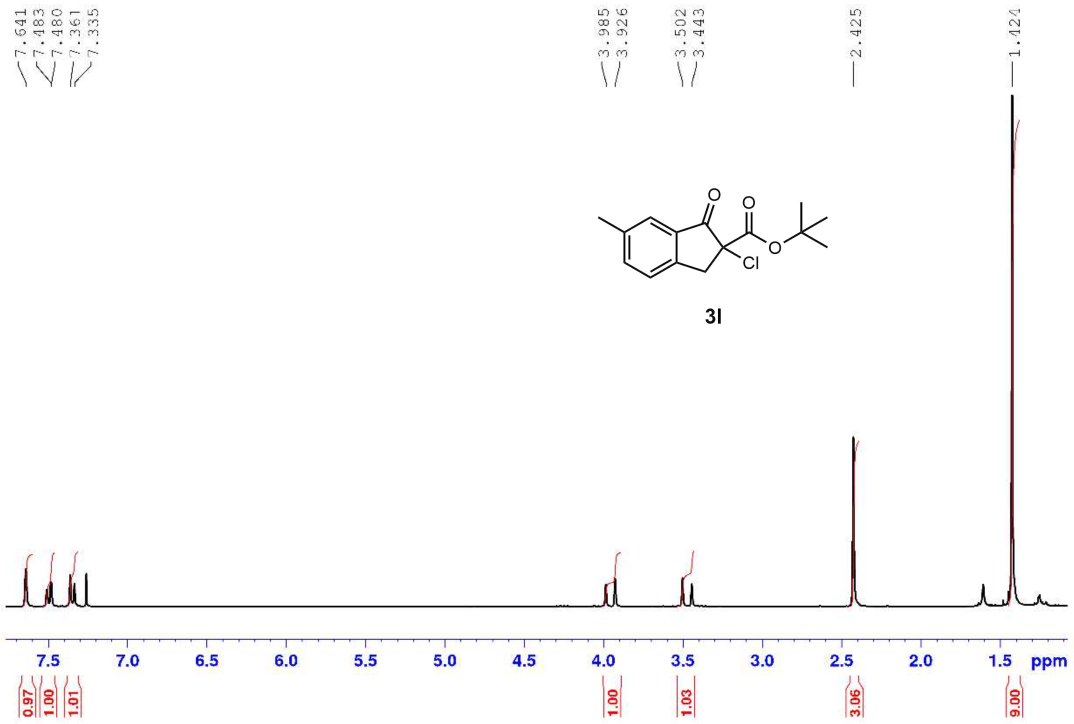

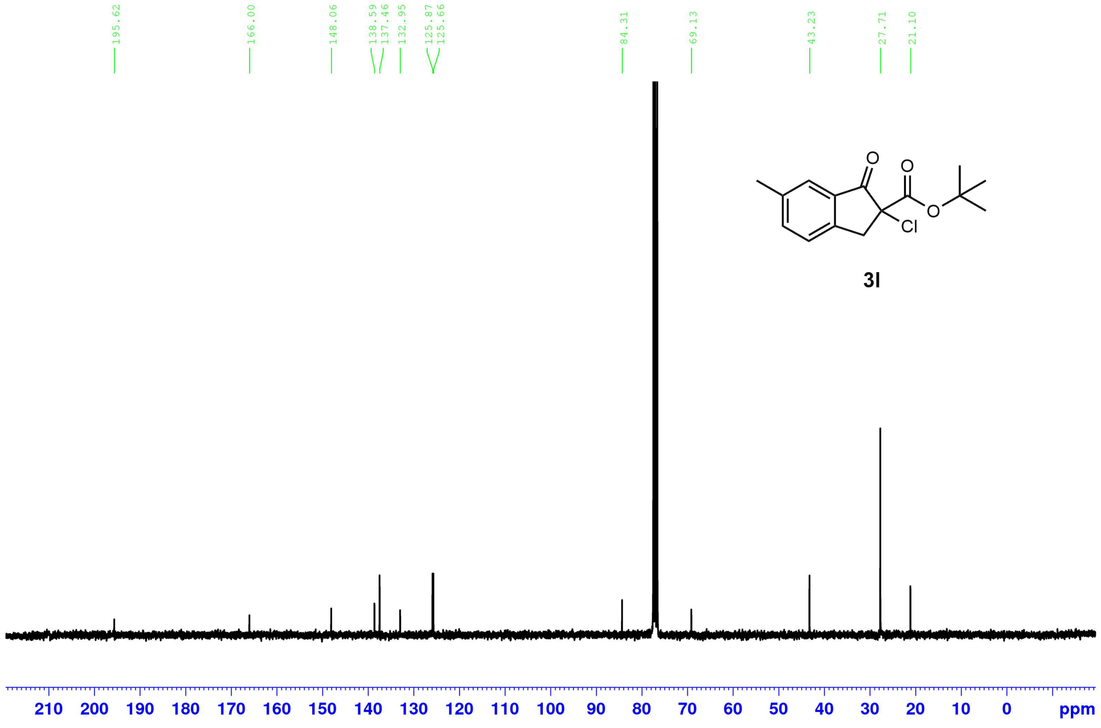

NMR spectra of **3m**:

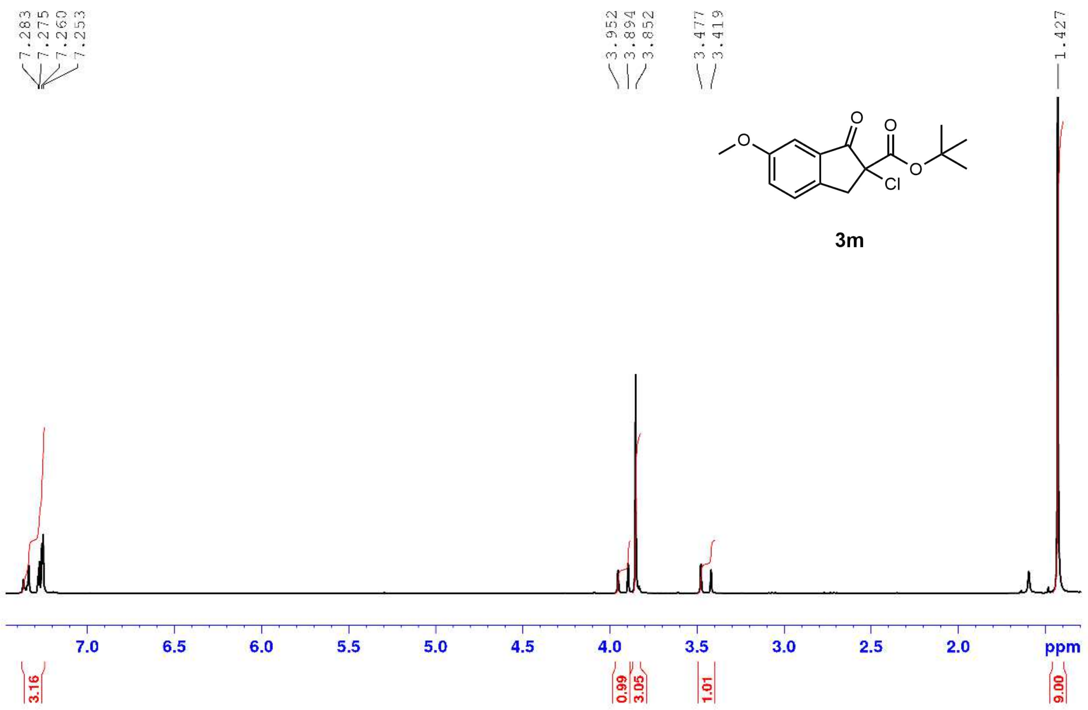

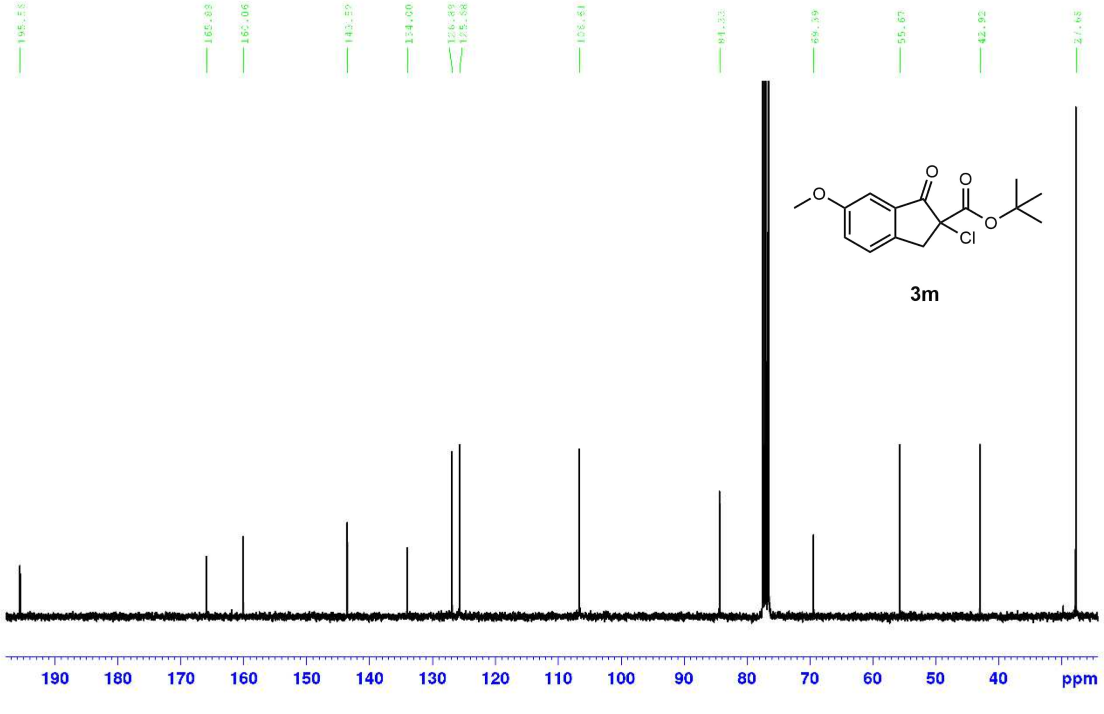

NMR spectra of **3n**:

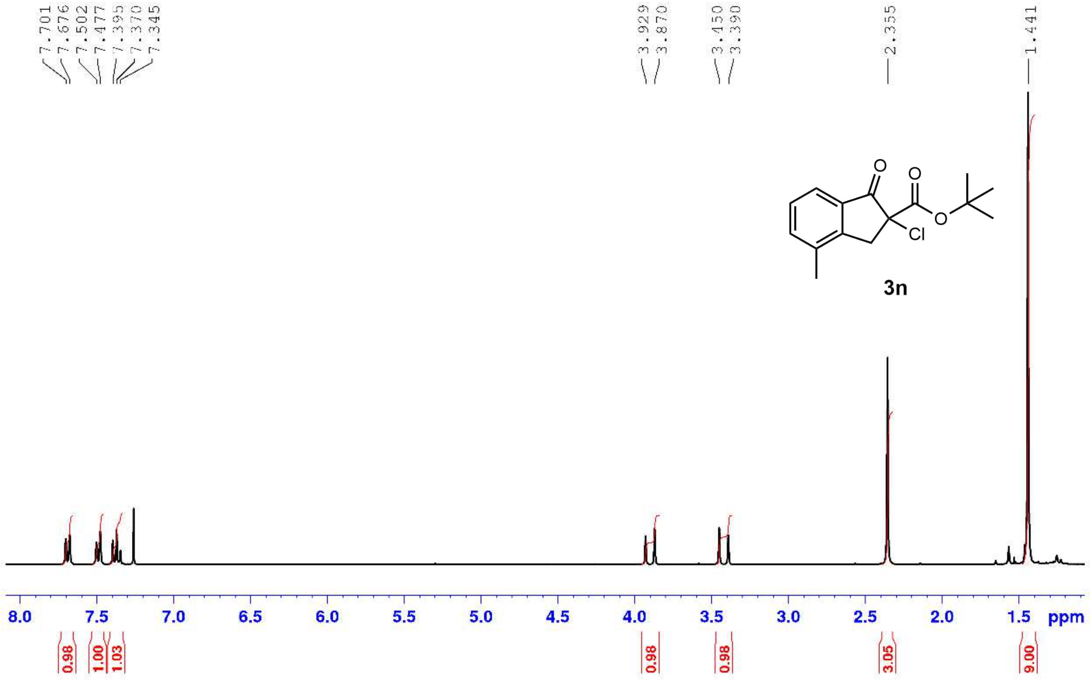

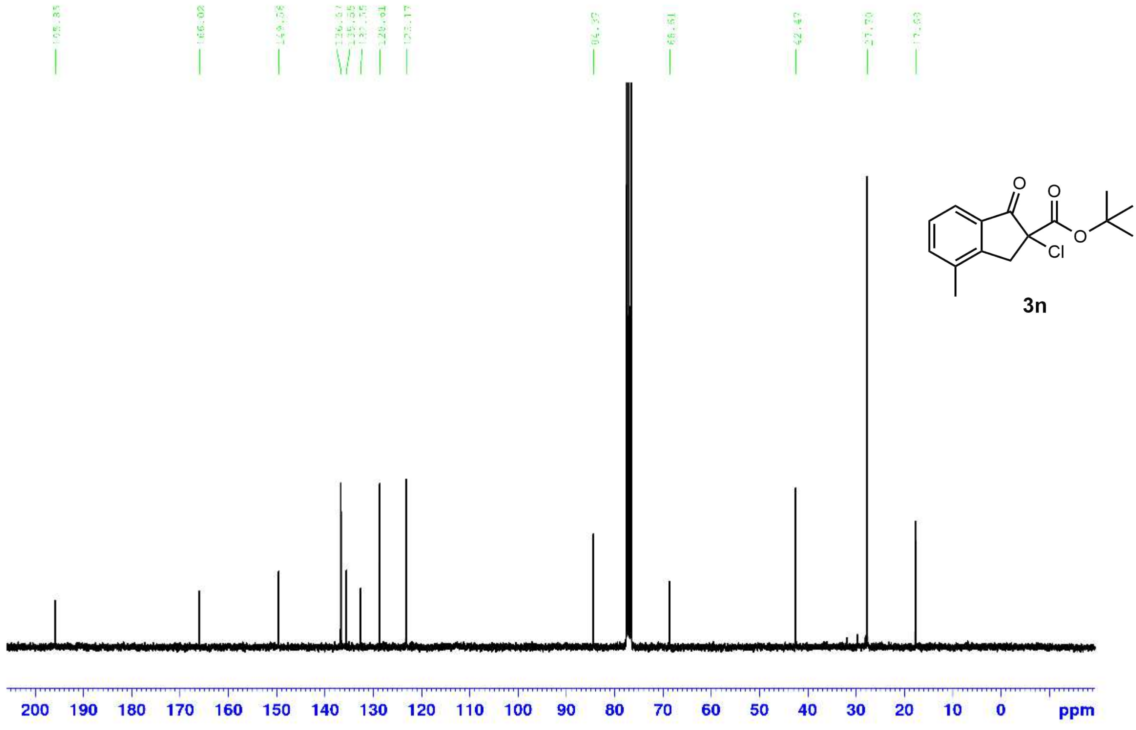

NMR spectra of **3o**:

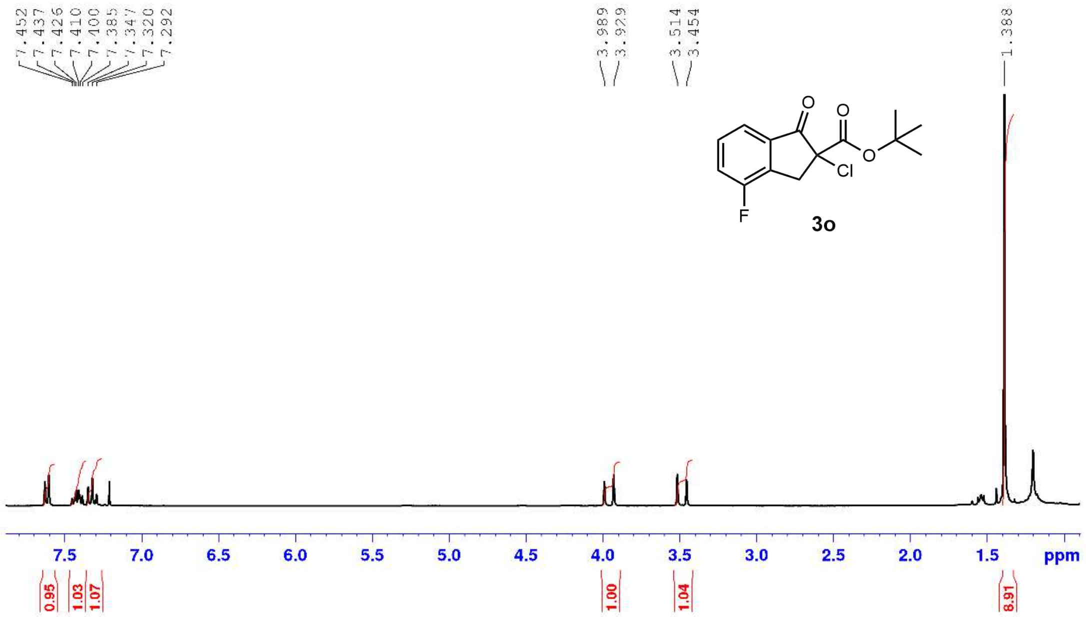

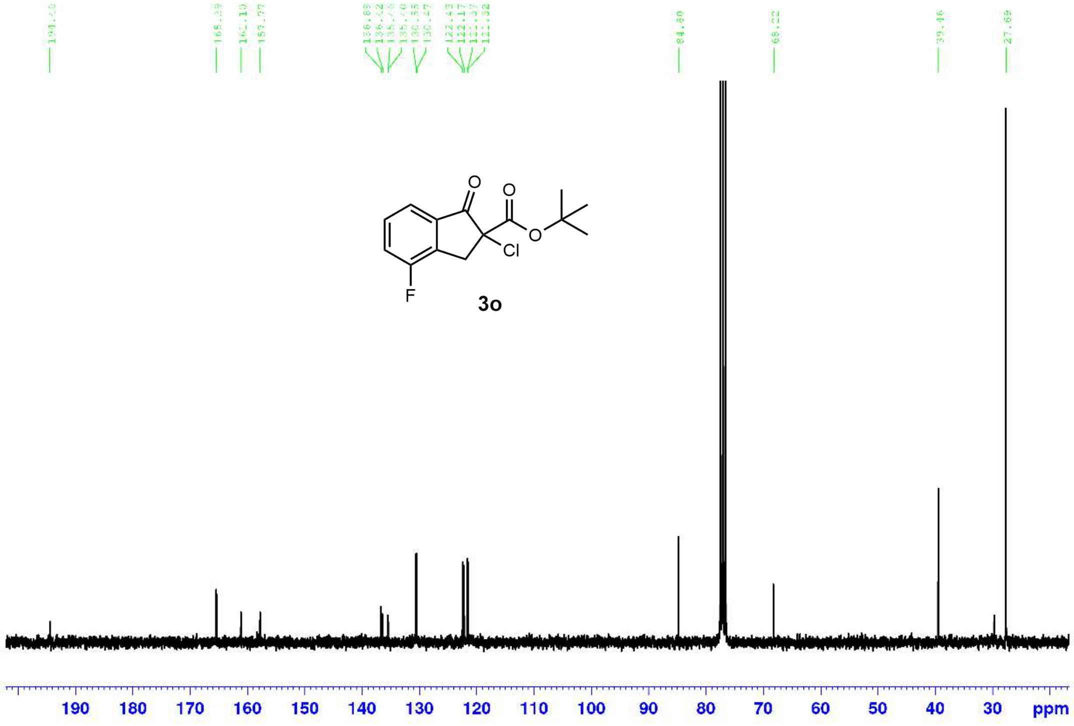

NMR spectra of **3p**:

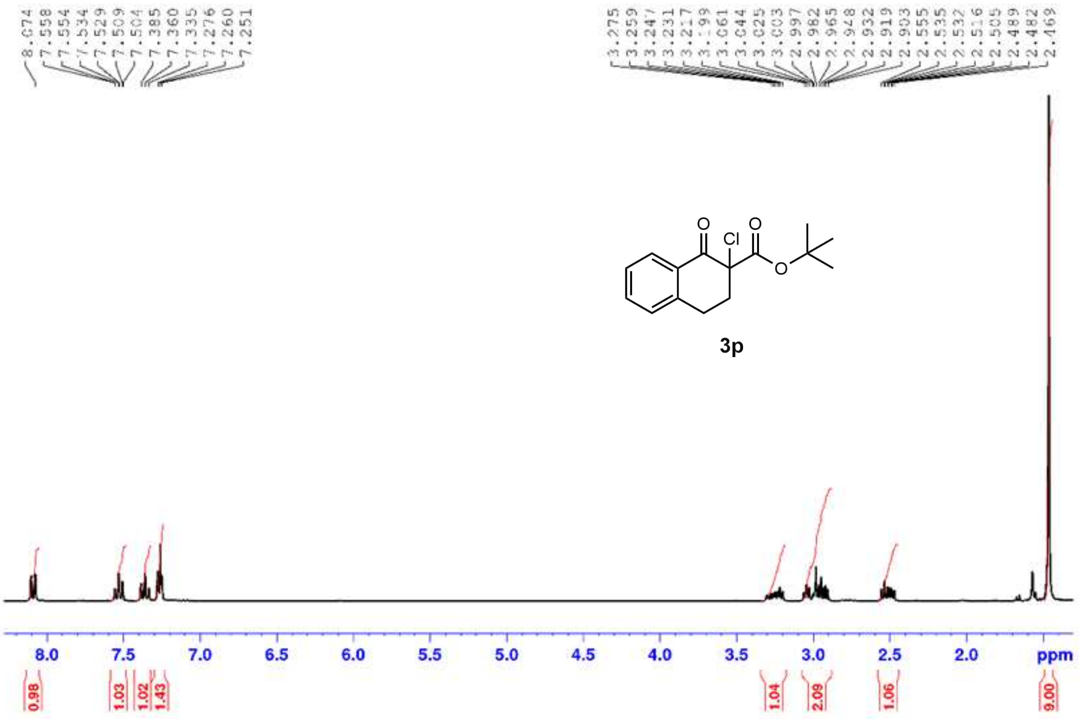

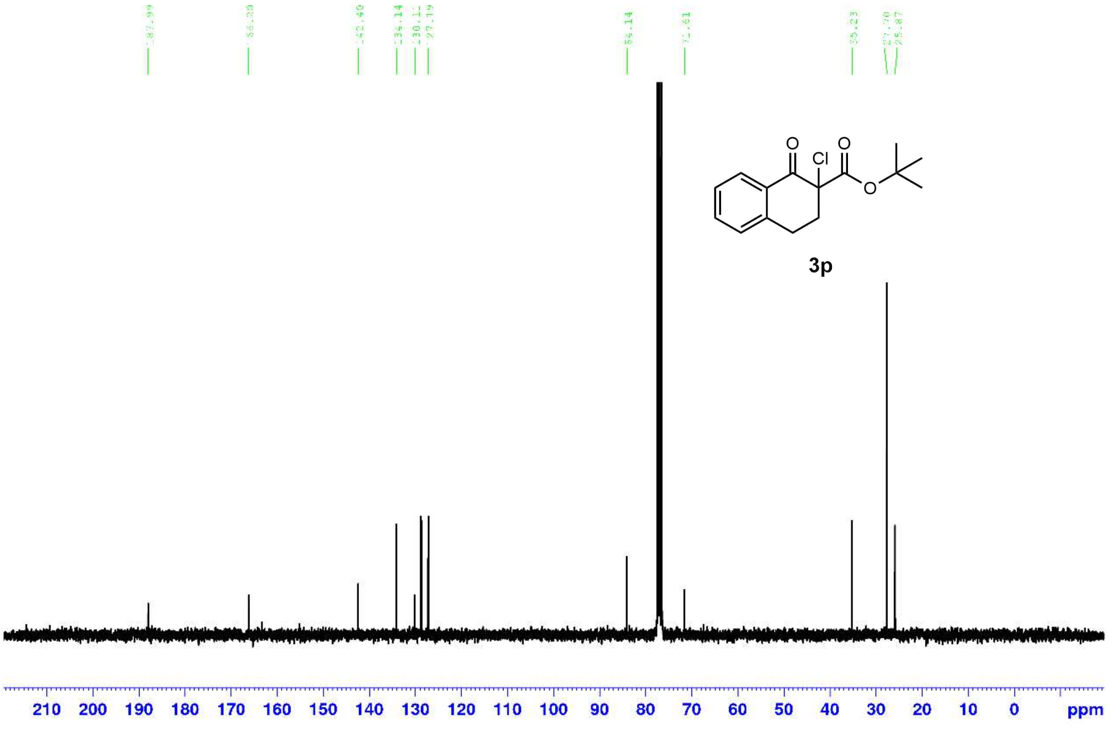

NMR spectra of **3q** (this compound decarboxylates easily during work and column chromatography and could not be isolated with higher purity):

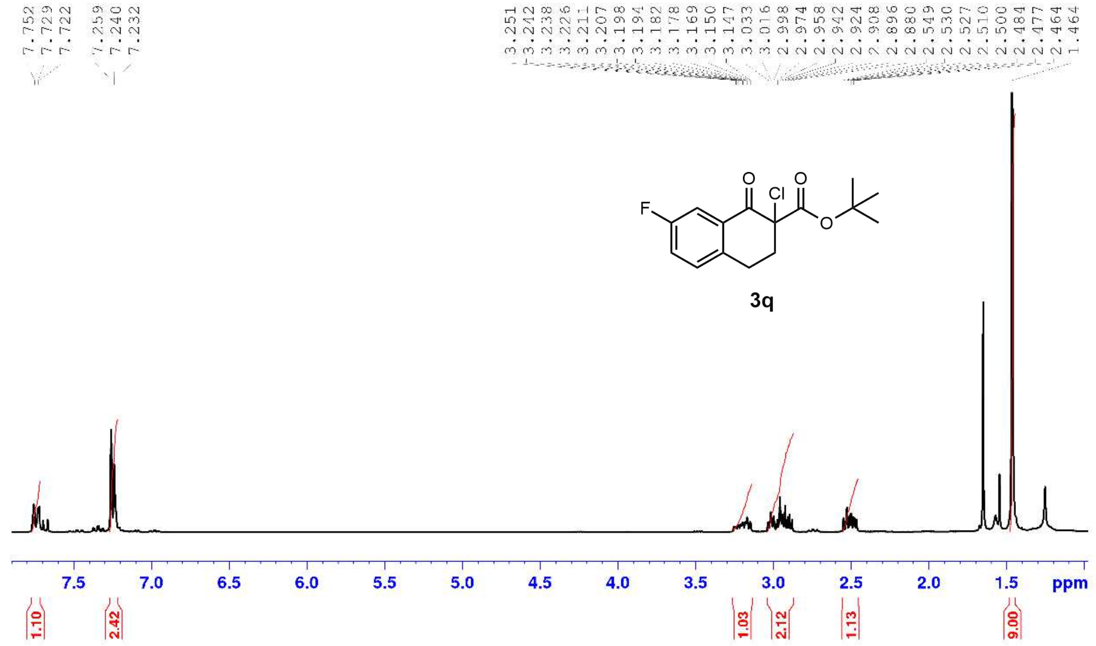

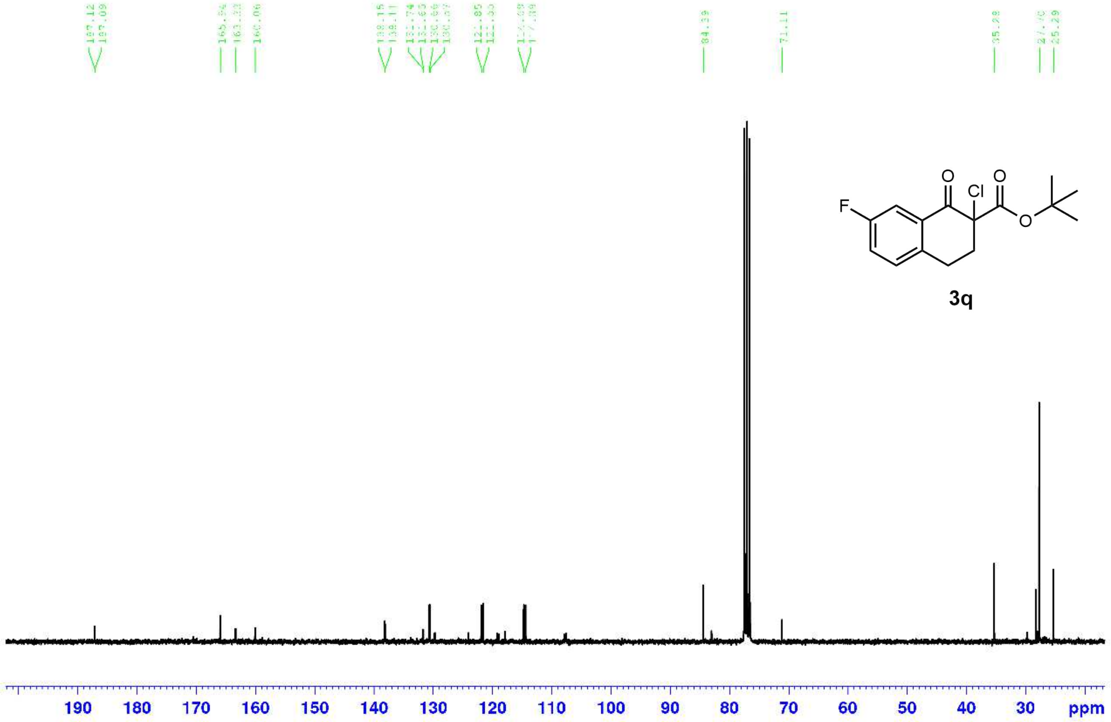

6. HPLC Traces of Chlorinated Products:

HPLC chromatogram of (*rac*)-**3a**:

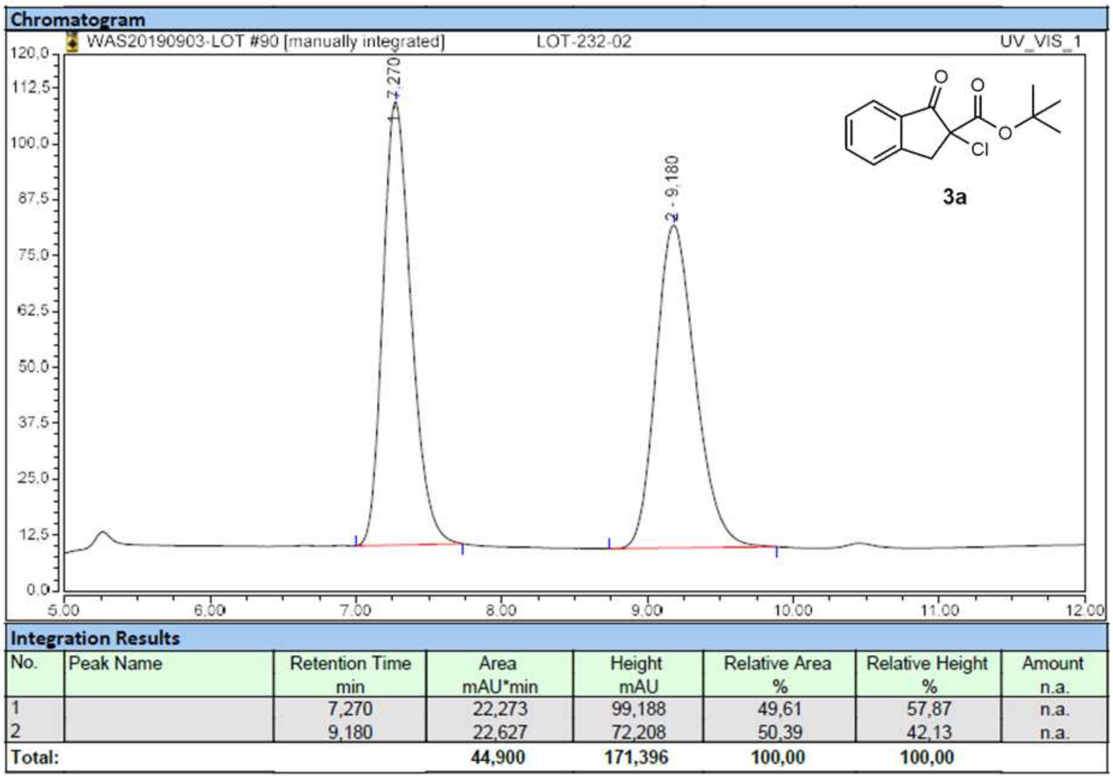

Integration Results

No.	Peak Name	Retention Time<br>min	Area<br>mAU*min	Height<br>mAU	Relative Area<br>%	Relative Height<br>%	Amount<br>n.a.
1		7,270	22,273	99,188	49,61	57,87	n.a.
2		9,180	22,627	72,208	50,39	42,13	n.a.
**Total:**			**44,900**	**171,396**	**100,00**	**100,00**	

HPLC chromatogram of enantioenriched **3a**:

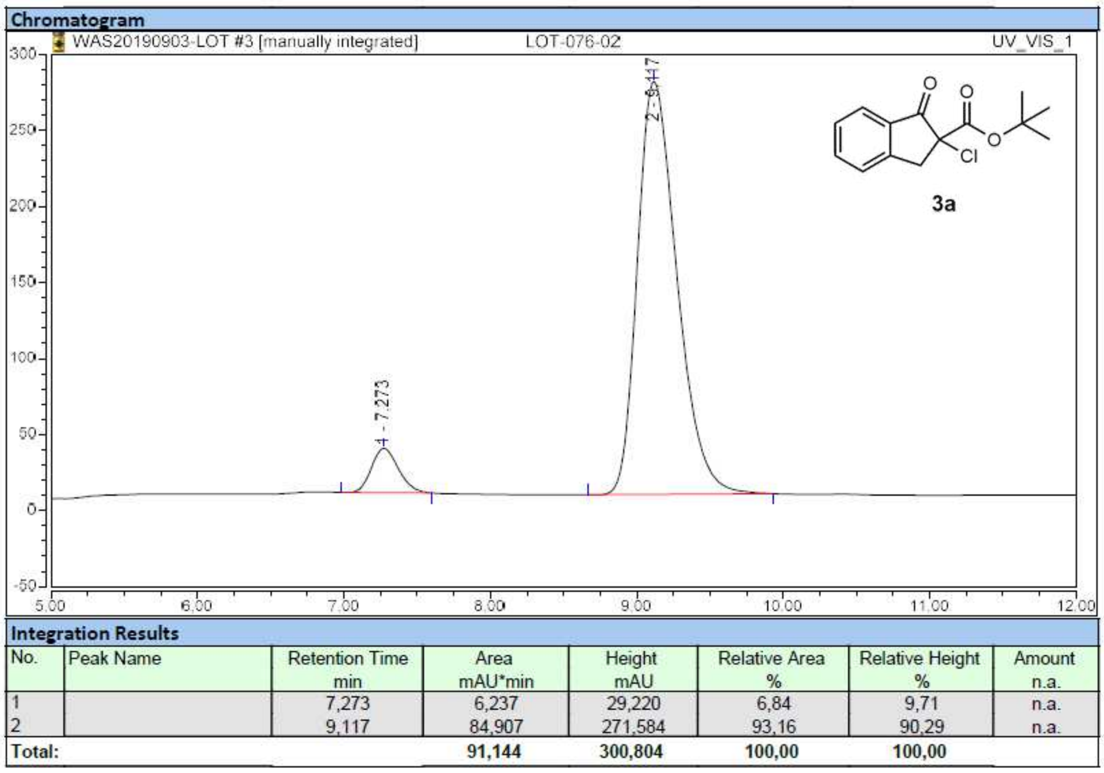

Integration Results

No.	Peak Name	Retention Time<br>min	Area<br>mAU*min	Height<br>mAU	Relative Area<br>%	Relative Height<br>%	Amount<br>n.a.
1		7,273	6,237	29,220	6,84	9,71	n.a.
2		9,117	84,907	271,584	93,16	90,29	n.a.
**Total:**			**91,144**	**300,804**	**100,00**	**100,00**	

HPLC chromatogram of (*rac*)-**3b**:

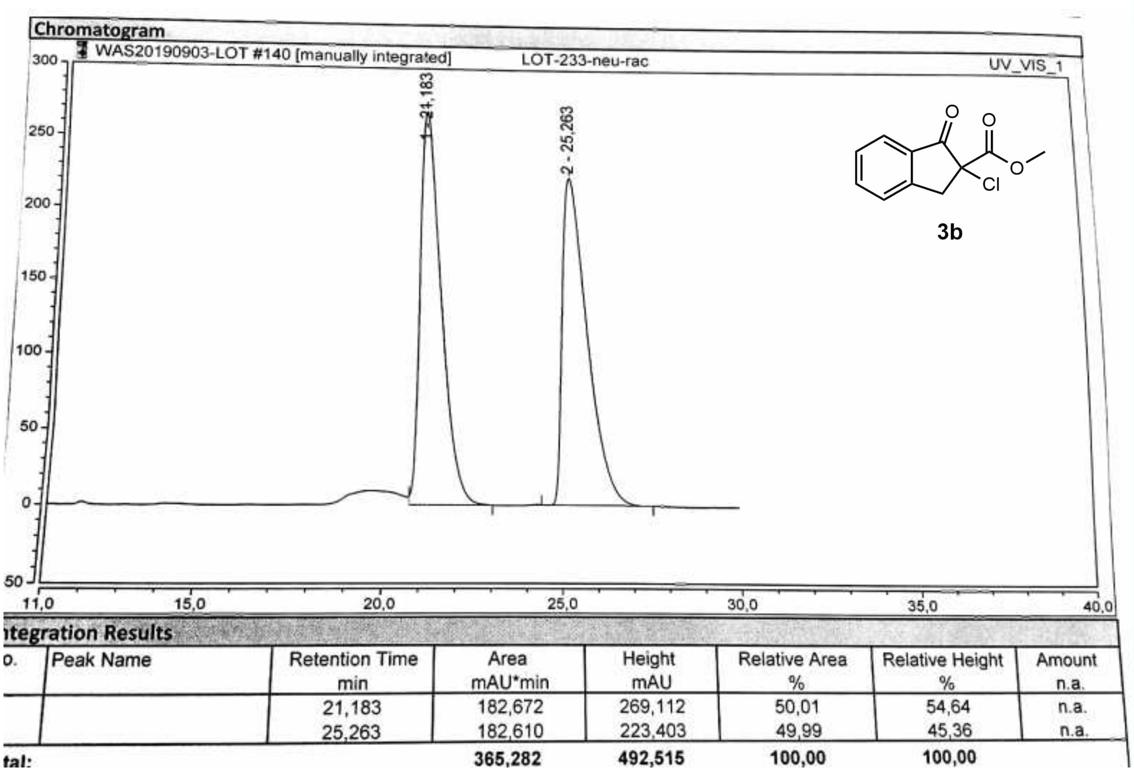

ntegration Results

o.	Peak Name	Retention Time min	Area mAU*min	Height mAU	Relative Area %	Relative Height %	Amount n.a.
		21,183	182,672	269,112	50,01	54,64	n.a.
		25,263	182,610	223,403	49,99	45,36	n.a.
tal:			365,282	492,515	100,00	100,00	

HPLC chromatogram of enantioenriched **3b**:

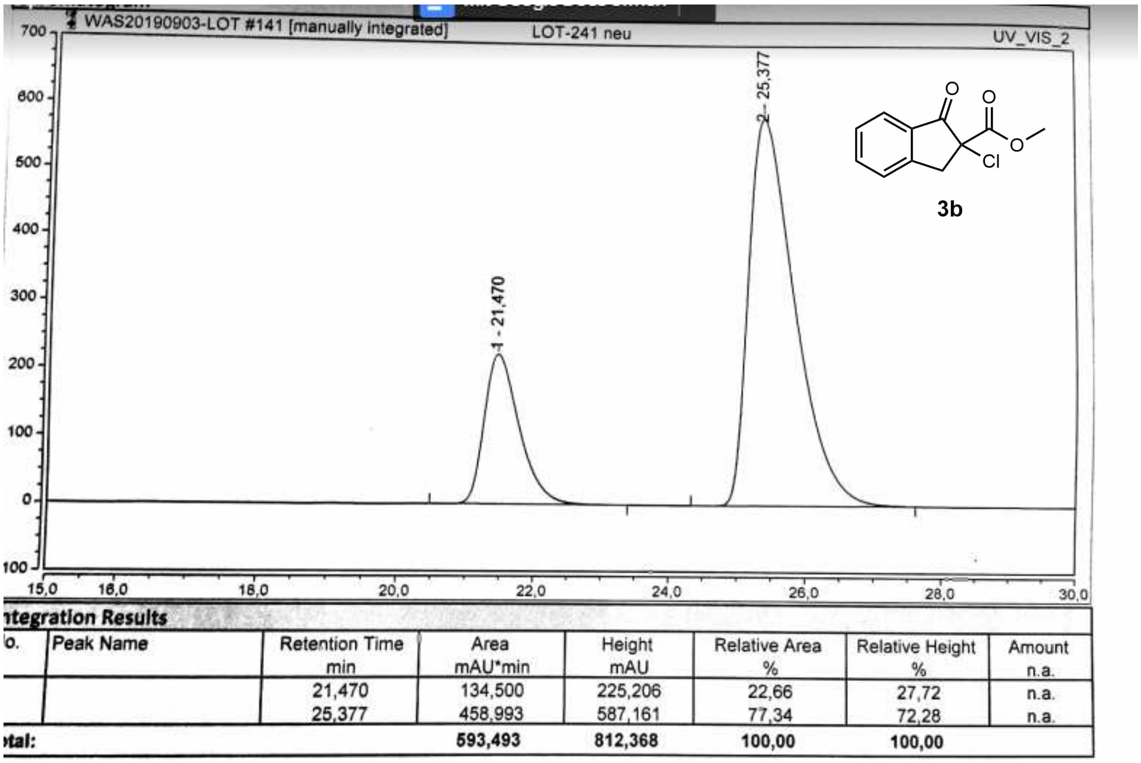

ntegration Results

o.	Peak Name	Retention Time min	Area mAU*min	Height mAU	Relative Area %	Relative Height %	Amount n.a.
		21,470	134,500	225,206	22,66	27,72	n.a.
		25,377	458,993	587,161	77,34	72,28	n.a.
tal:			593,493	812,368	100,00	100,00	

HPLC chromatogram of (*rac*)-**3c**:

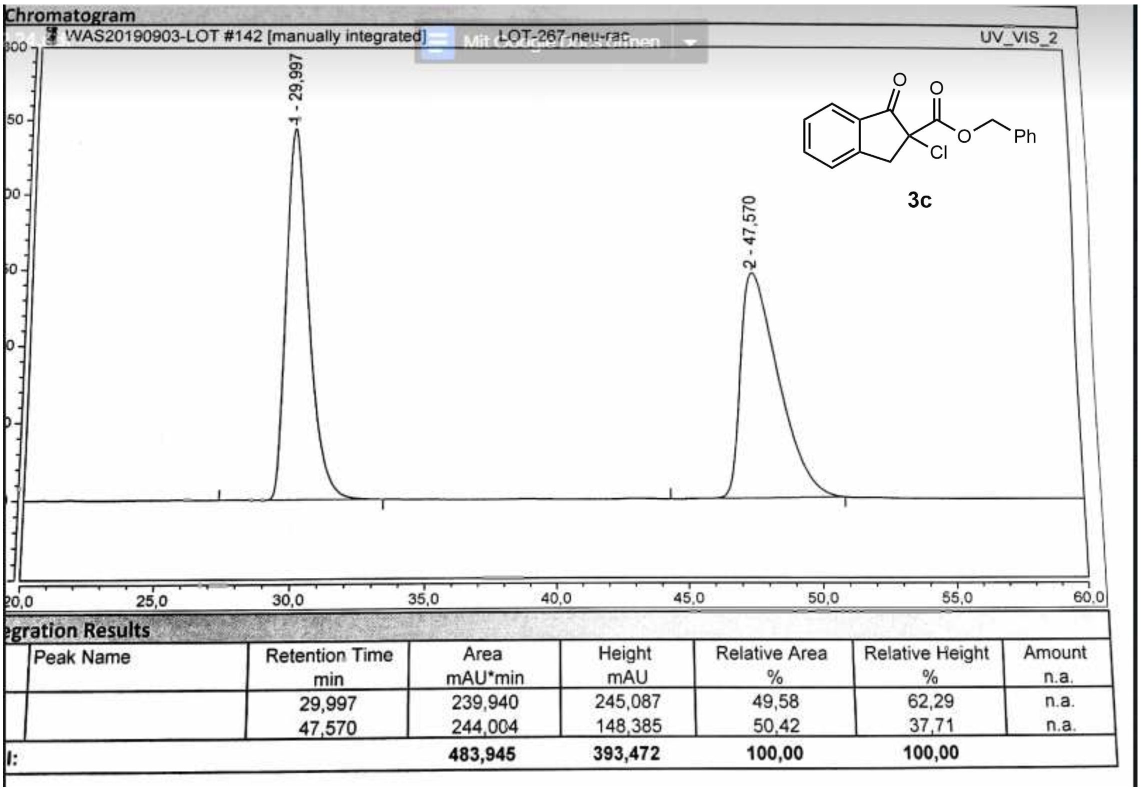

egration Results

	Peak Name	Retention Time min	Area mAU*min	Height mAU	Relative Area %	Relative Height %	Amount n.a.
		29,997	239,940	245,087	49,58	62,29	n.a.
		47,570	244,004	148,385	50,42	37,71	n.a.
l:			483,945	393,472	100,00	100,00	

HPLC chromatogram of enantioenriched **3c**:

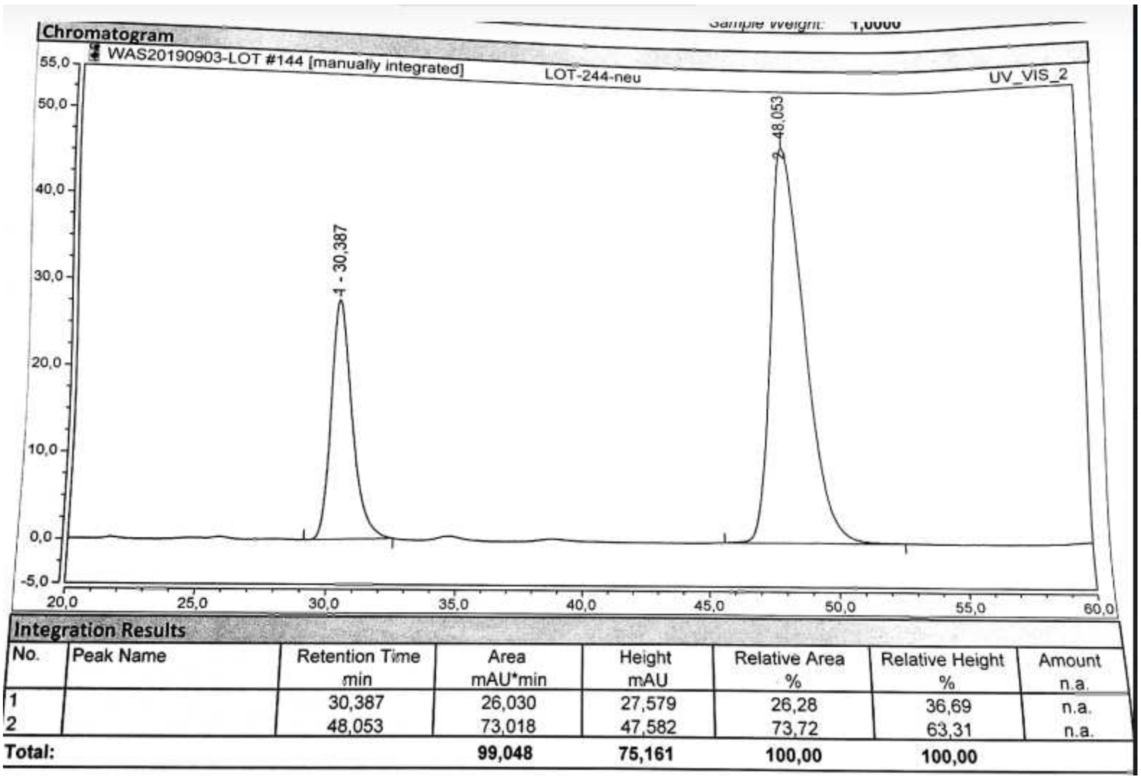

Integration Results

No.	Peak Name	Retention Time min	Area mAU*min	Height mAU	Relative Area %	Relative Height %	Amount n.a.
1		30,387	26,030	27,579	26,28	36,69	n.a.
2		48,053	73,018	47,582	73,72	63,31	n.a.
Total:			99,048	75,161	100,00	100,00	

HPLC chromatogram of (*rac*)-**3e**:

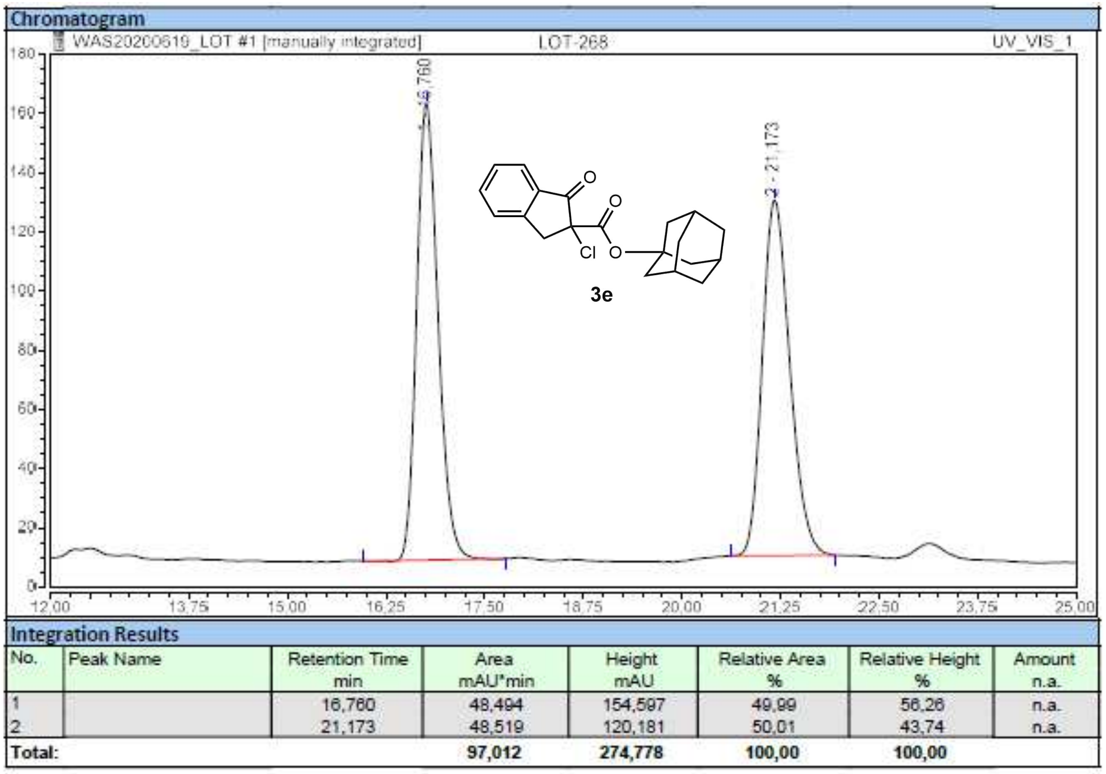

Integration Results

No.	Peak Name	Retention Time min	Area mAU*min	Height mAU	Relative Area %	Relative Height %	Amount n.a.
1		16,760	48,494	154,597	49,99	56,26	n.a.
2		21,173	48,519	120,181	50,01	43,74	n.a.
**Total:**			**97,012**	**274,778**	**100,00**	**100,00**	

HPLC chromatogram of enantioenriched **3e**:

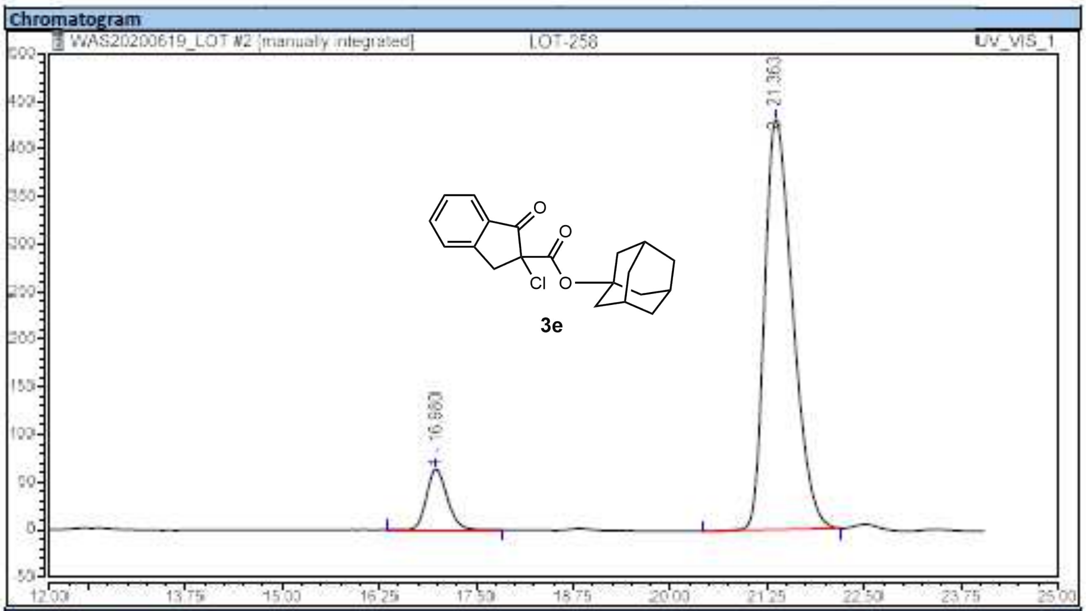

Integration Results

No.	Peak Name	Retention Time min	Area mAU*min	Height mAU	Relative Area %	Relative Height %	Amount n.a.
1		16,980	19,487	63,528	9,74	12,85	n.a.
2		21,363	180,590	431,010	90,26	87,15	n.a.
**Total:**			**200,077**	**494,539**	**100,00**	**100,00**	

HPLC chromatogram of (*rac*)-**3f**:

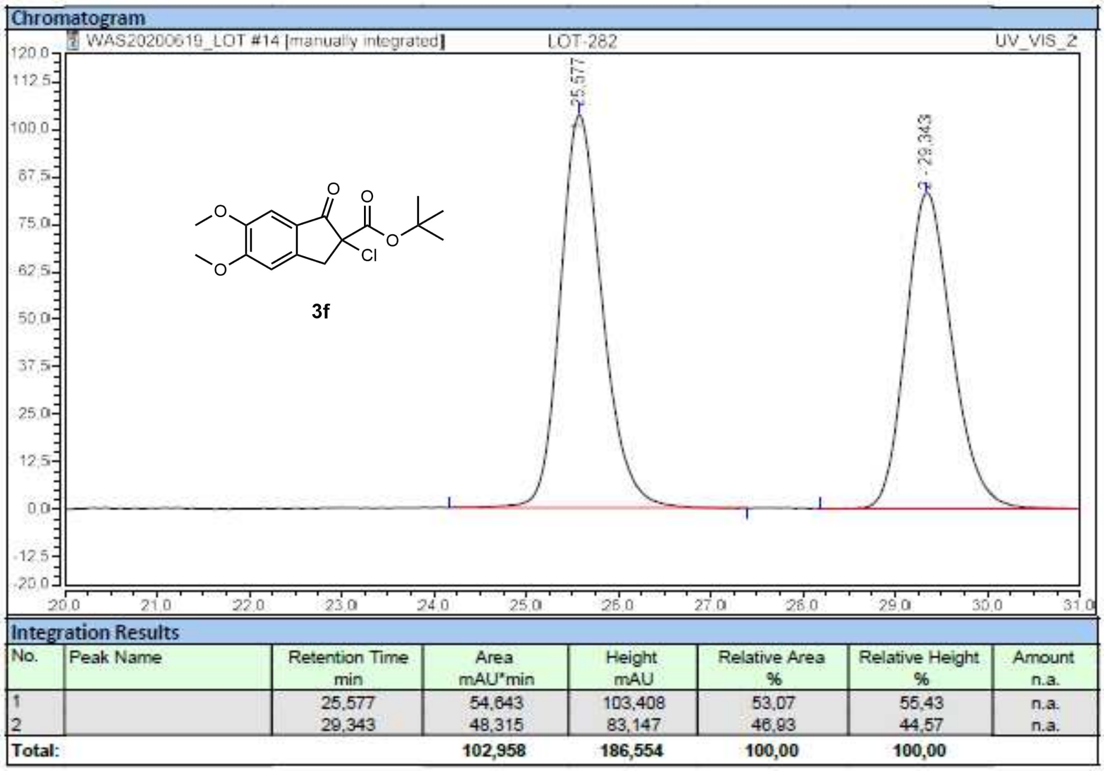

Integration Results

No.	Peak Name	Retention Time min	Area mAU*min	Height mAU	Relative Area %	Relative Height %	Amount n.a.
1		25,577	54,643	103,408	53,07	55,43	n.a.
2		29,343	48,315	83,147	46,93	44,57	n.a.
**Total:**			**102,958**	**186,554**	**100,00**	**100,00**	

HPLC chromatogram of enantioenriched **3f**:

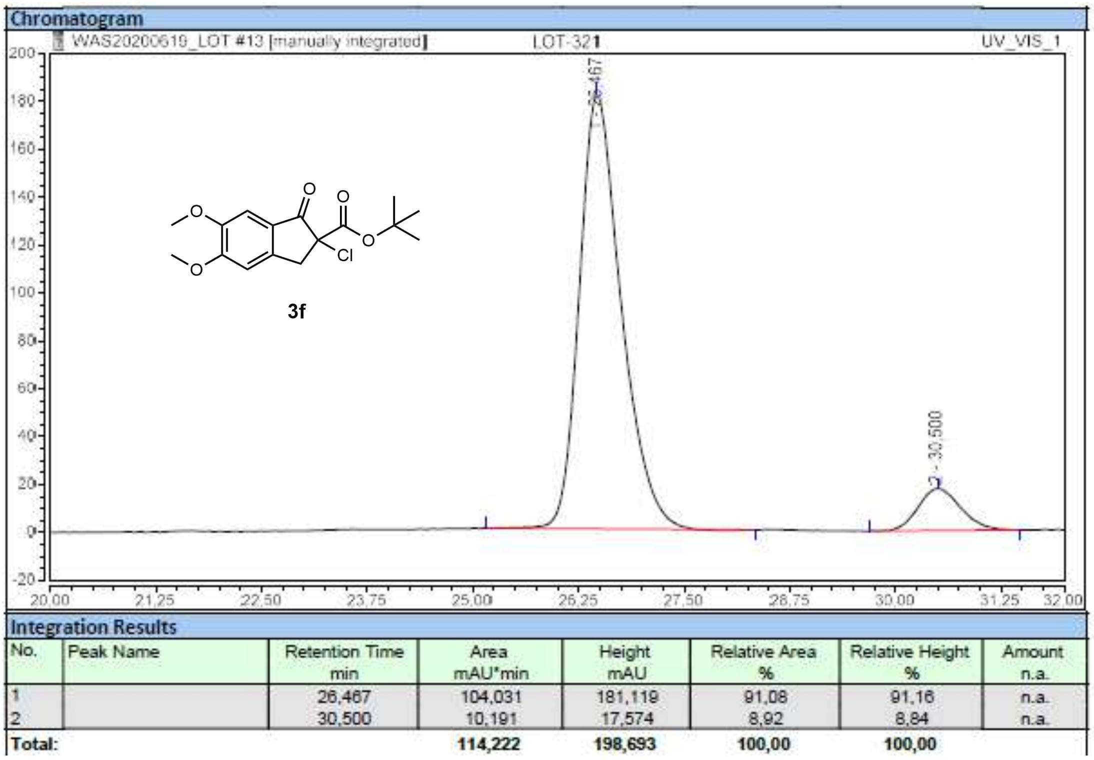

Integration Results

No.	Peak Name	Retention Time min	Area mAU*min	Height mAU	Relative Area %	Relative Height %	Amount n.a.
1		26,467	104,031	181,119	91,08	91,16	n.a.
2		30,500	10,191	17,574	8,92	8,84	n.a.
**Total:**			**114,222**	**198,693**	**100,00**	**100,00**	

HPLC chromatogram of (*rac*)-**3g**:

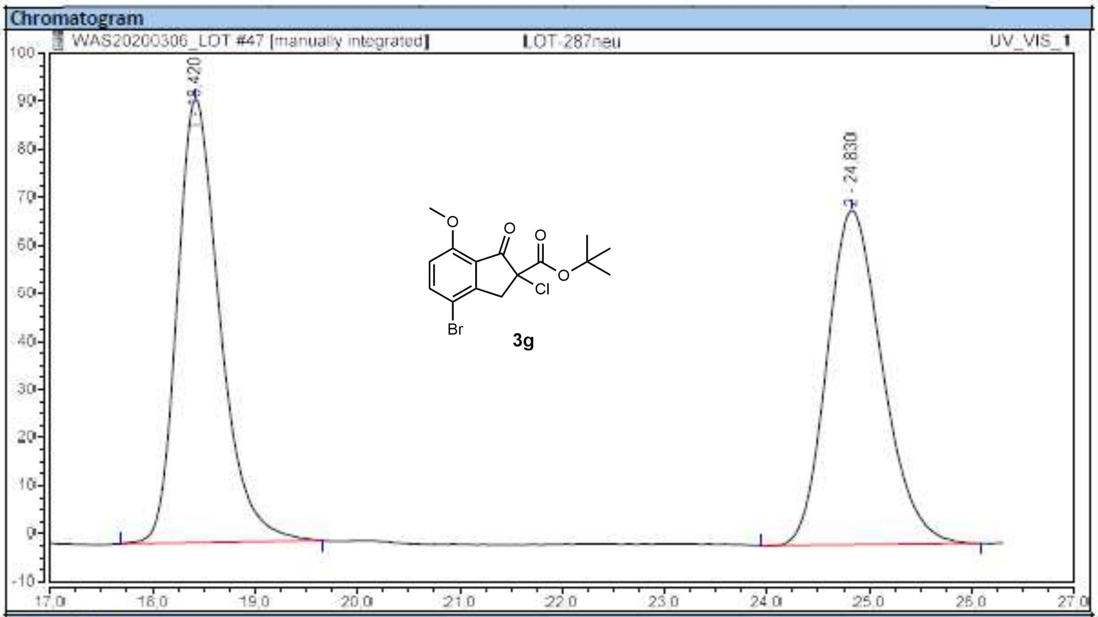

Integration Results

No.	Peak Name	Retention Time min	Area mAU*min	Height mAU	Relative Area %	Relative Height %	Amount n.a.
1		18,420	44,562	92,306	50,60	57,02	n.a.
2		24,830	43,498	69,585	49,40	42,98	n.a.
**Total:**			**88,060**	**161,891**	**100,00**	**100,00**	

HPLC chromatogram of enantioenriched **3g**:

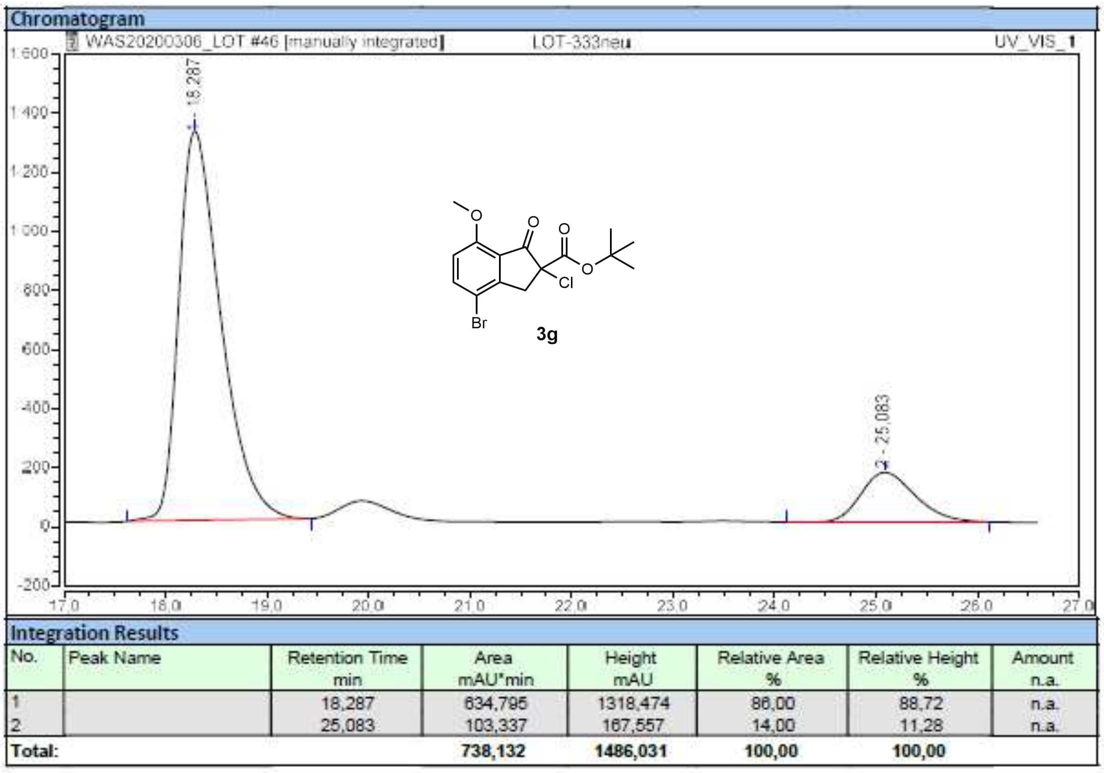

Integration Results

No.	Peak Name	Retention Time min	Area mAU*min	Height mAU	Relative Area %	Relative Height %	Amount n.a.
1		18,287	634,795	1318,474	86,00	88,72	n.a.
2		25,083	103,337	167,557	14,00	11,28	n.a.
**Total:**			**738,132**	**1486,031**	**100,00**	**100,00**	

HPLC chromatogram of (*rac*)-**3h**:

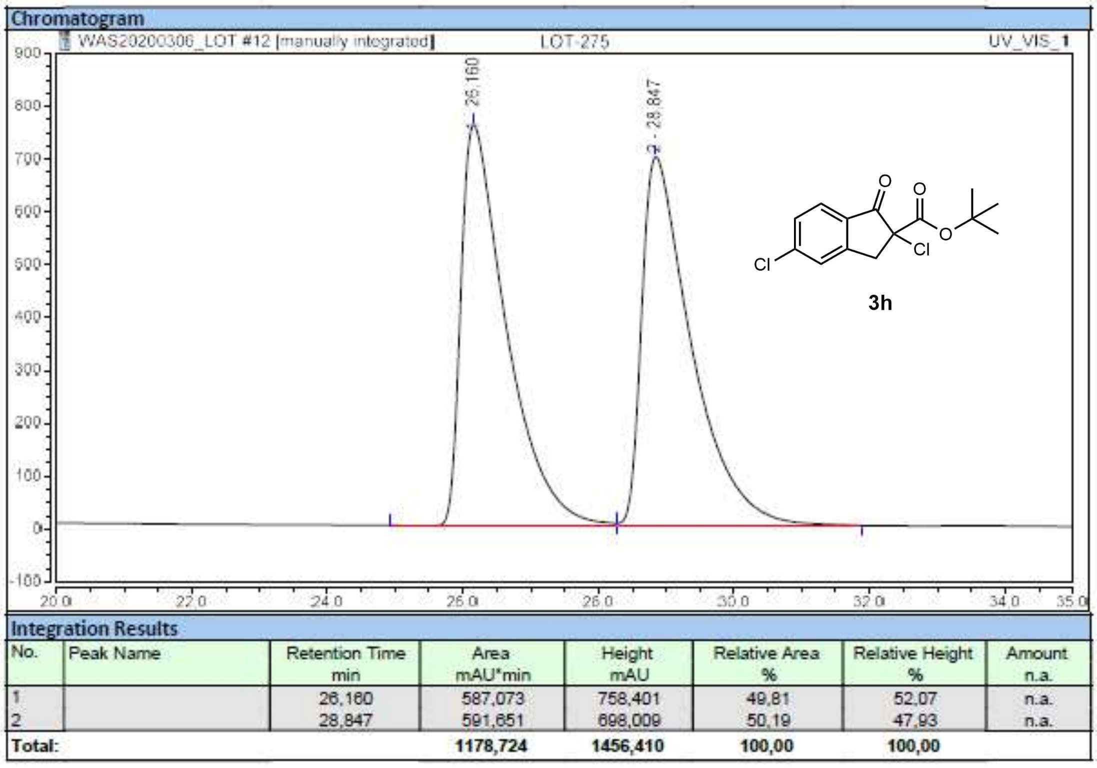

Integration Results

No.	Peak Name	Retention Time min	Area mAU*min	Height mAU	Relative Area %	Relative Height %	Amount n.a.
1		26,160	587,073	758,401	49,81	52,07	n.a.
2		28,847	591,651	698,009	50,19	47,93	n.a.
**Total:**			**1178,724**	**1456,410**	**100,00**	**100,00**	

HPLC chromatogram of enantioenriched **3h**:

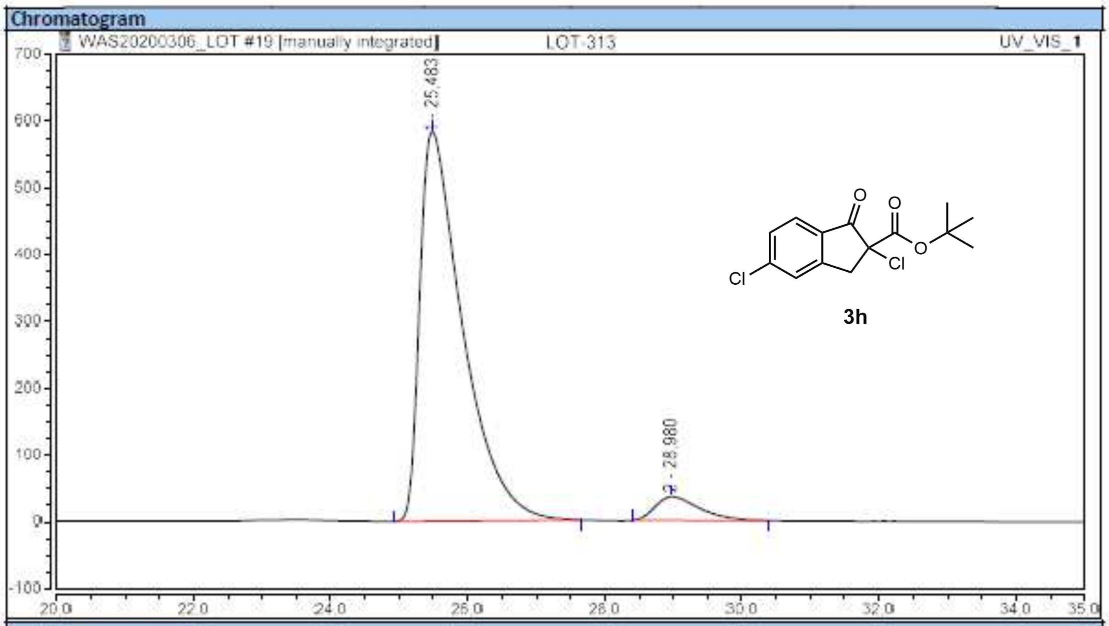

Integration Results

No.	Peak Name	Retention Time min	Area mAU*min	Height mAU	Relative Area %	Relative Height %	Amount n.a.
1		25,483	422,287	583,633	94,08	94,27	n.a.
2		28,980	26,586	35,454	5,92	5,73	n.a.
**Total:**			**448,872**	**619,087**	**100,00**	**100,00**	

HPLC chromatogram of (*rac*)-**3i**:

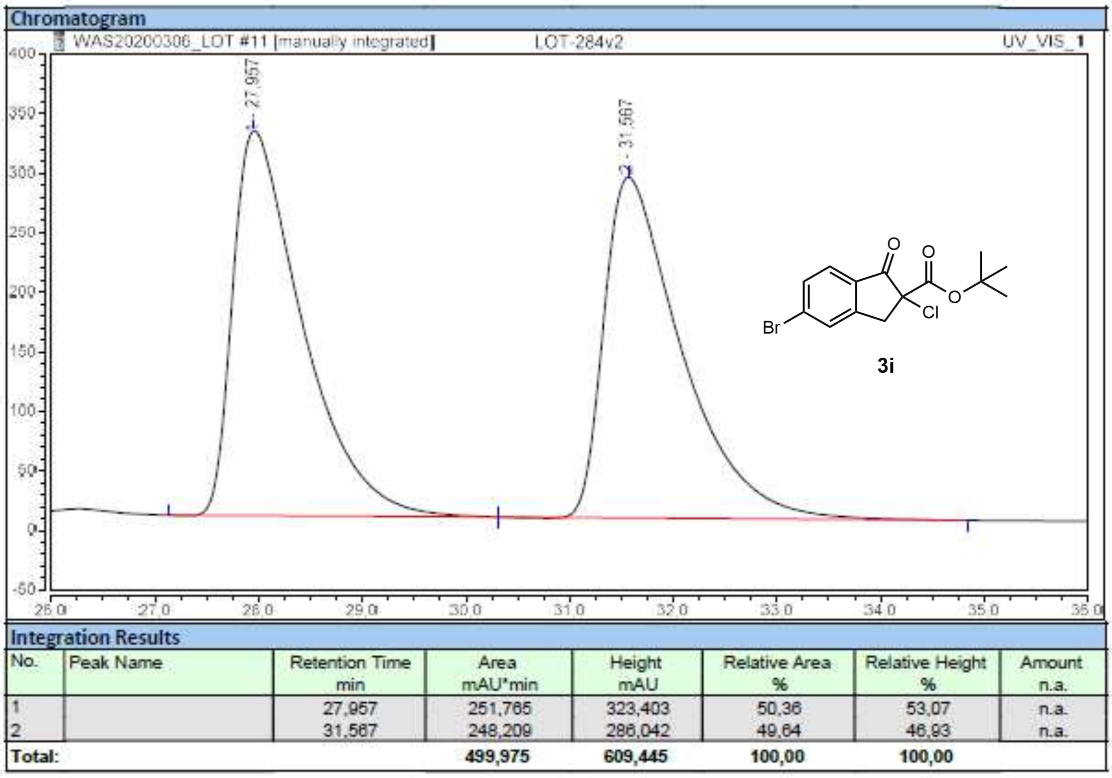

Integration Results

No.	Peak Name	Retention Time min	Area mAU*min	Height mAU	Relative Area %	Relative Height %	Amount n.a.
1		27,957	251,765	323,403	50,36	53,07	n.a.
2		31,567	248,209	286,042	49,64	46,93	n.a.
**Total:**			**499,975**	**609,445**	**100,00**	**100,00**	

HPLC chromatogram of enantioenriched **3i**:

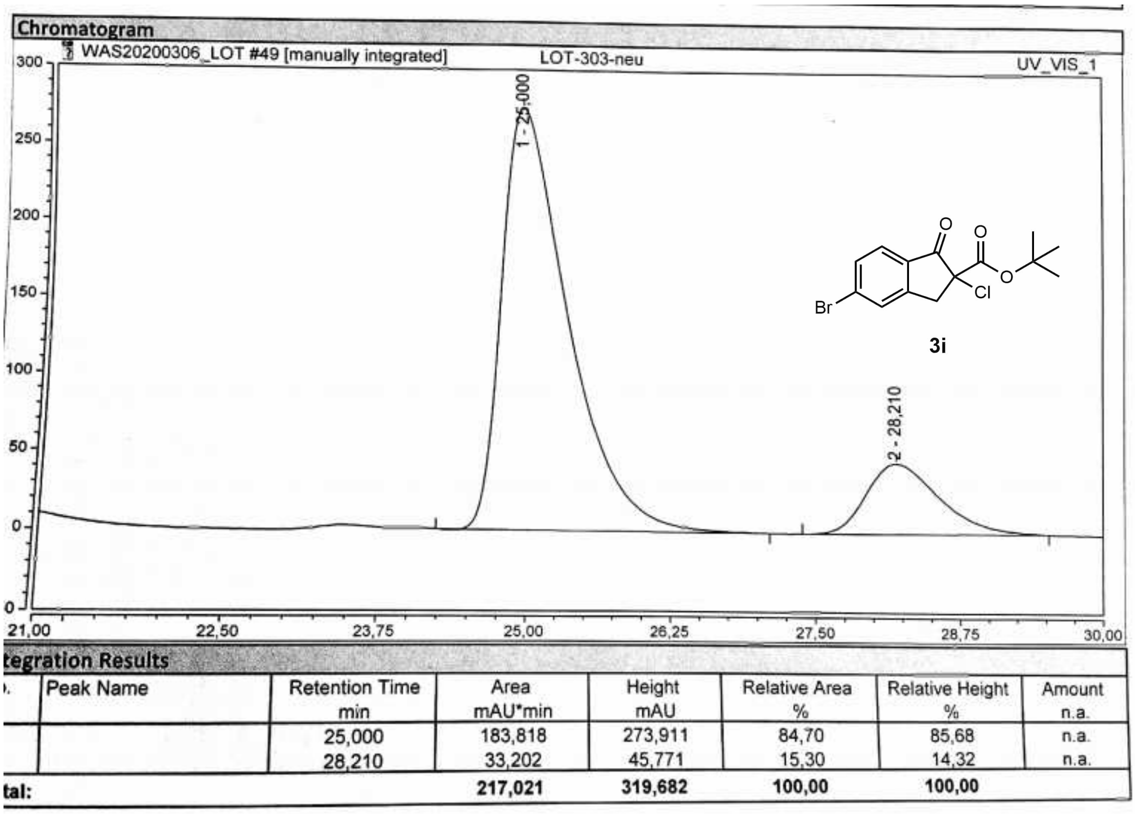

tegration Results

.	Peak Name	Retention Time min	Area mAU*min	Height mAU	Relative Area %	Relative Height %	Amount n.a.
		25,000	183,818	273,911	84,70	85,68	n.a.
		28,210	33,202	45,771	15,30	14,32	n.a.
**tal:**			**217,021**	**319,682**	**100,00**	**100,00**	

HPLC chromatogram of (*rac*)-**3j**:

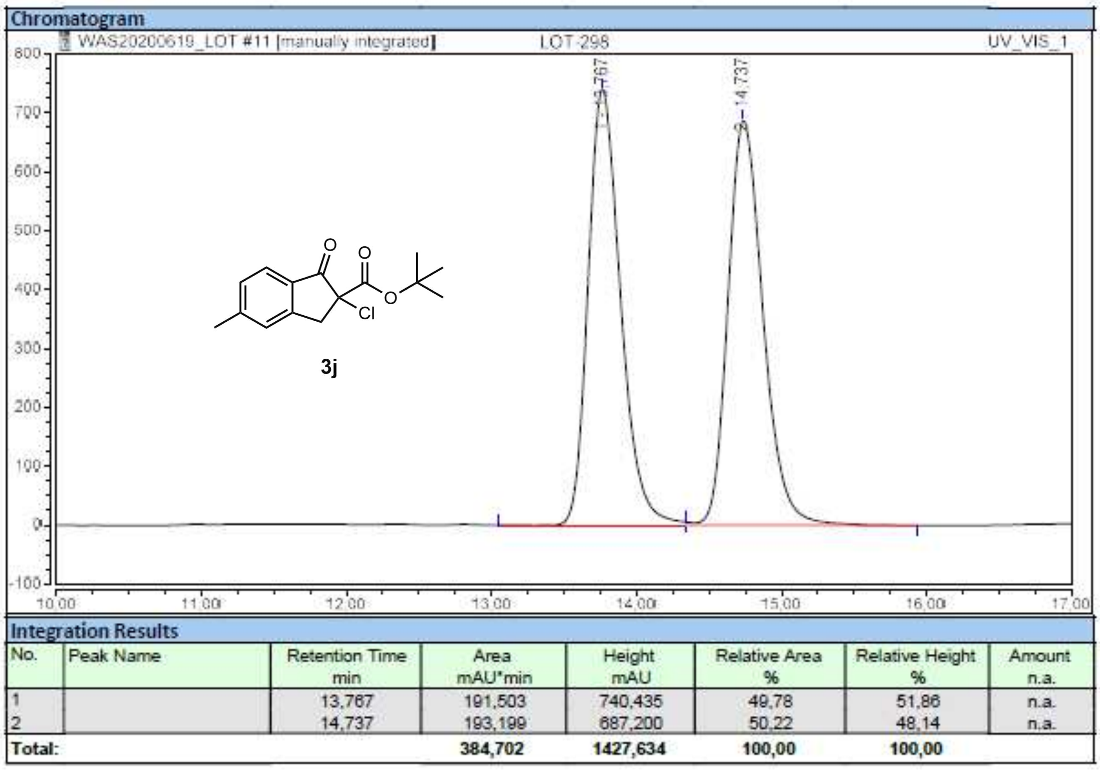

Integration Results

No.	Peak Name	Retention Time min	Area mAU*min	Height mAU	Relative Area %	Relative Height %	Amount n.a.
1		13,767	191,503	740,435	49,78	51,86	n.a.
2		14,737	193,199	687,200	50,22	48,14	n.a.
**Total:**			**384,702**	**1427,634**	**100,00**	**100,00**	

HPLC chromatogram of enantioenriched **3j**:

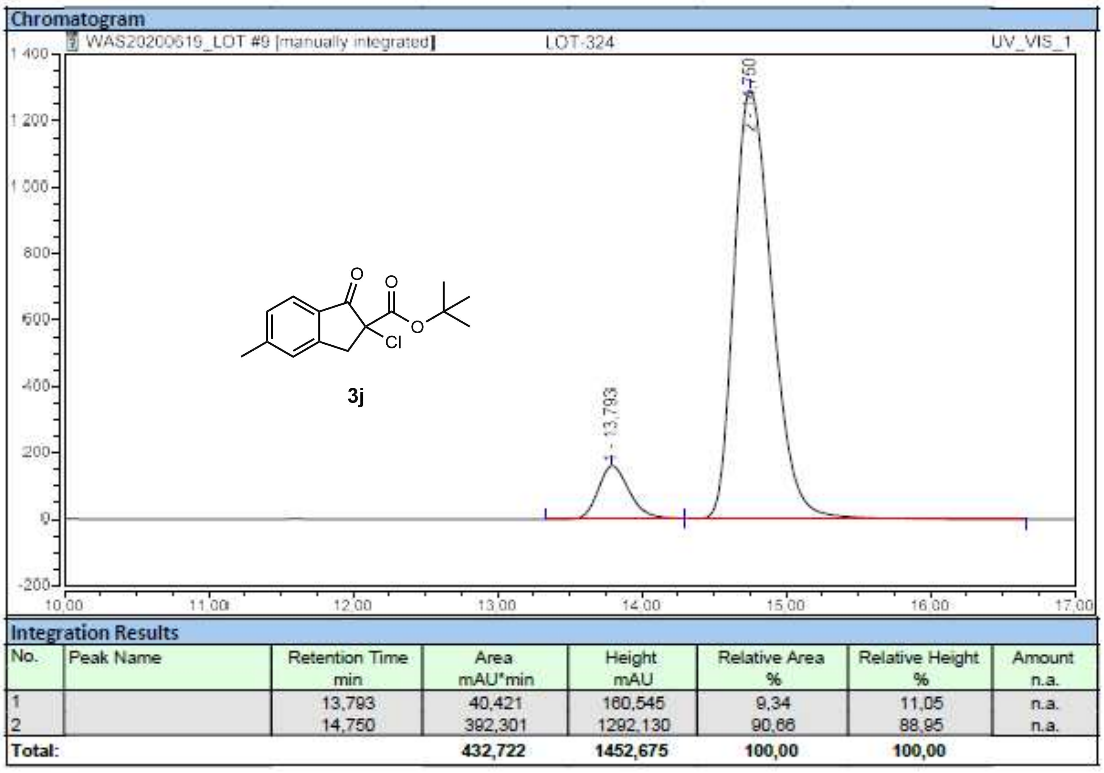

Integration Results

No.	Peak Name	Retention Time min	Area mAU*min	Height mAU	Relative Area %	Relative Height %	Amount n.a.
1		13,793	40,421	160,545	9,34	11,05	n.a.
2		14,750	392,301	1292,130	90,66	88,95	n.a.
**Total:**			**432,722**	**1452,675**	**100,00**	**100,00**	

HPLC chromatogram of (*rac*)-**3k**:

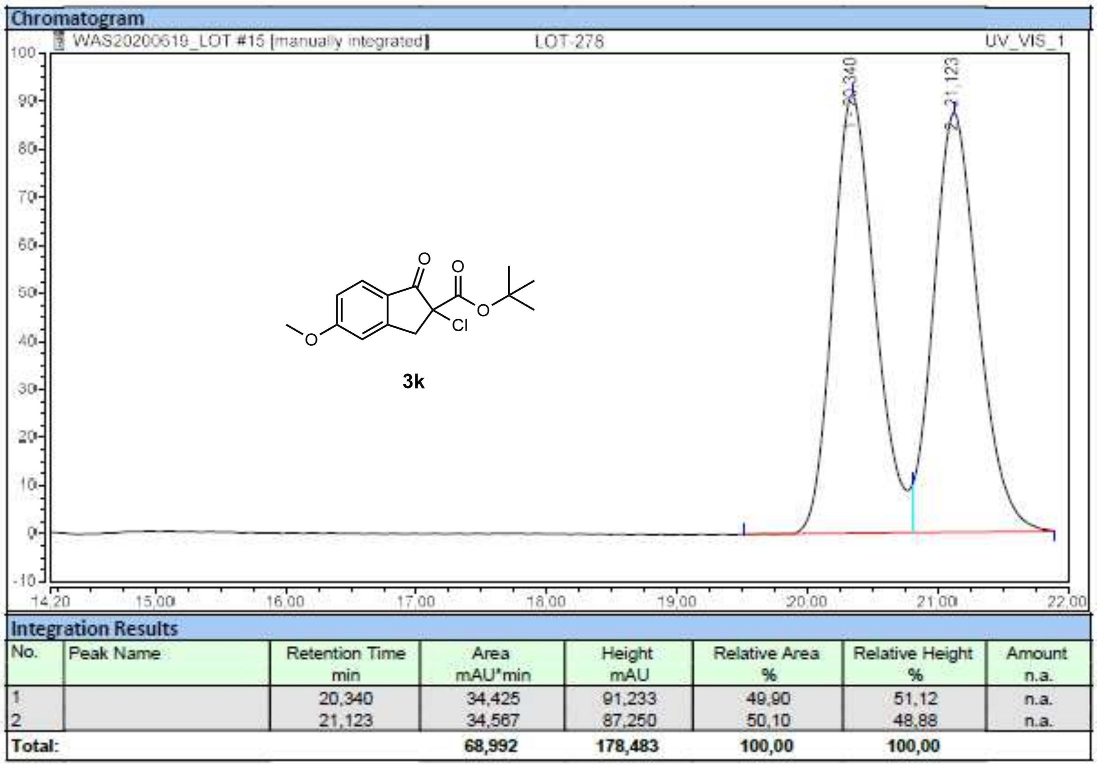

Integration Results

No.	Peak Name	Retention Time min	Area mAU*min	Height mAU	Relative Area %	Relative Height %	Amount n.a.
1		20,340	34,425	91,233	49,90	51,12	n.a.
2		21,123	34,567	87,250	50,10	48,88	n.a.
**Total:**			**68,992**	**178,483**	**100,00**	**100,00**	

HPLC chromatogram of enantioenriched **3k**:

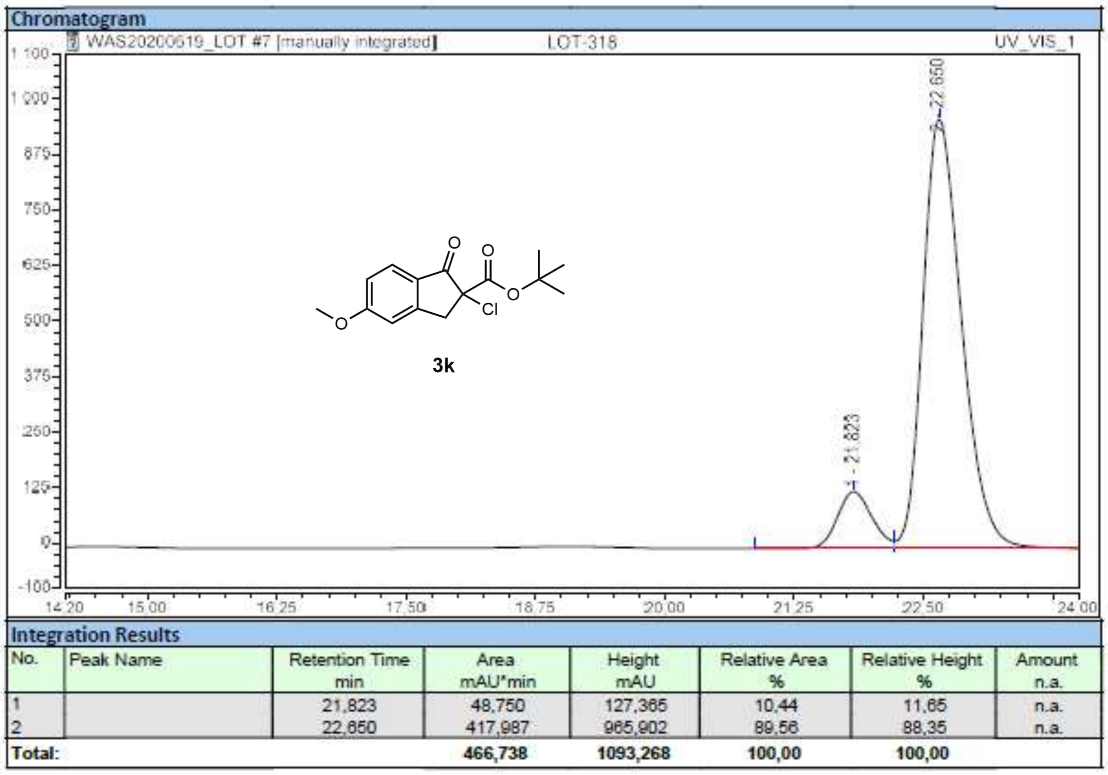

Integration Results

No.	Peak Name	Retention Time min	Area mAU*min	Height mAU	Relative Area %	Relative Height %	Amount n.a.
1		21,823	48,750	127,365	10,44	11,65	n.a.
2		22,650	417,987	965,902	89,56	88,35	n.a.
**Total:**			**466,738**	**1093,268**	**100,00**	**100,00**	

HPLC chromatogram of (*rac*)-**3l**:

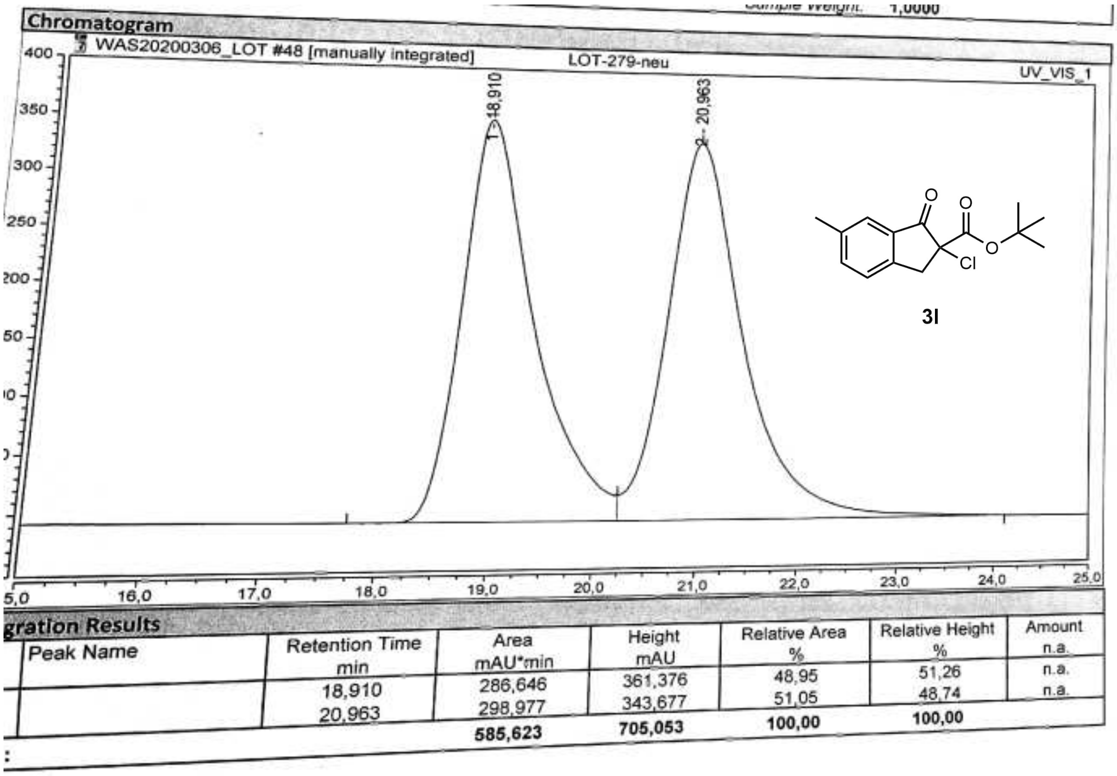

gration Results

Peak Name	Retention Time min	Area mAU*min	Height mAU	Relative Area %	Relative Height %	Amount n.a.
	18,910	286,646	361,376	48,95	51,26	n.a.
	20,963	298,977	343,677	51,05	48,74	n.a.
		585,623	705,053	100,00	100,00	

HPLC chromatogram of enantioenriched **3l**:

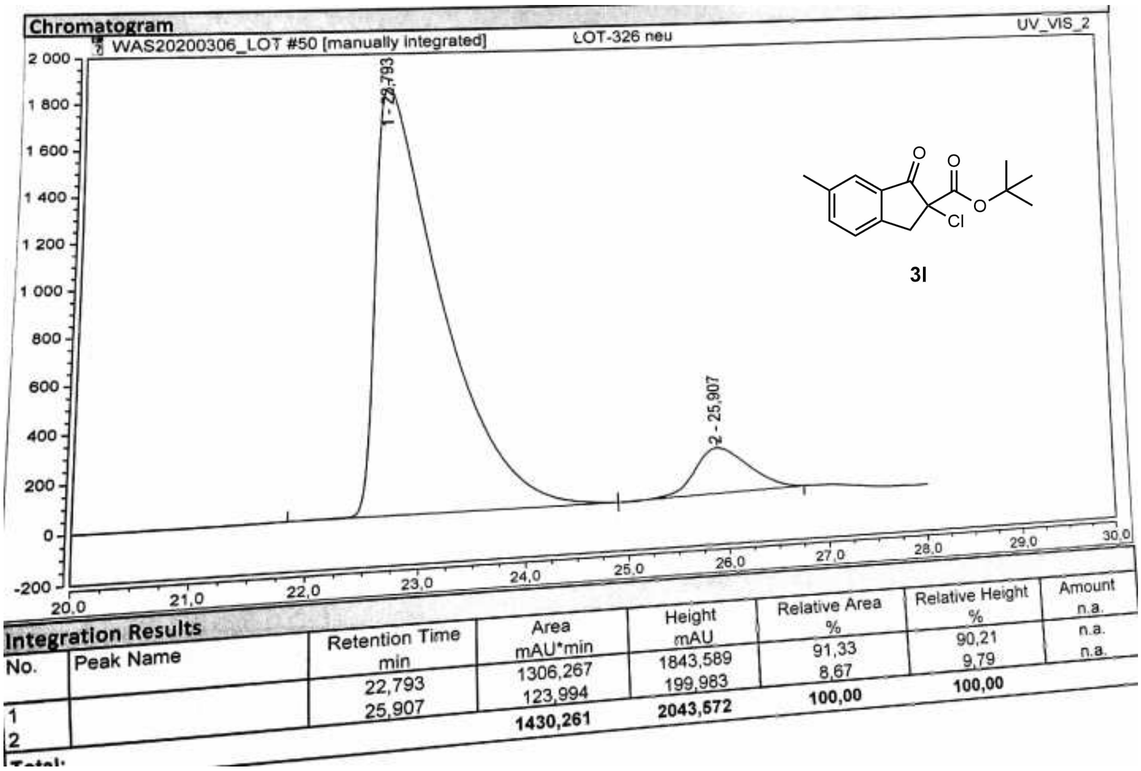

Integration Results

No.	Peak Name	Retention Time min	Area mAU*min	Height mAU	Relative Area %	Relative Height %	Amount n.a.
1		22,793	1306,267	1843,589	91,33	90,21	n.a.
2		25,907	123,994	199,983	8,67	9,79	n.a.
Total:			1430,261	2043,572	100,00	100,00	

HPLC chromatogram of (*rac*)-**3m**:

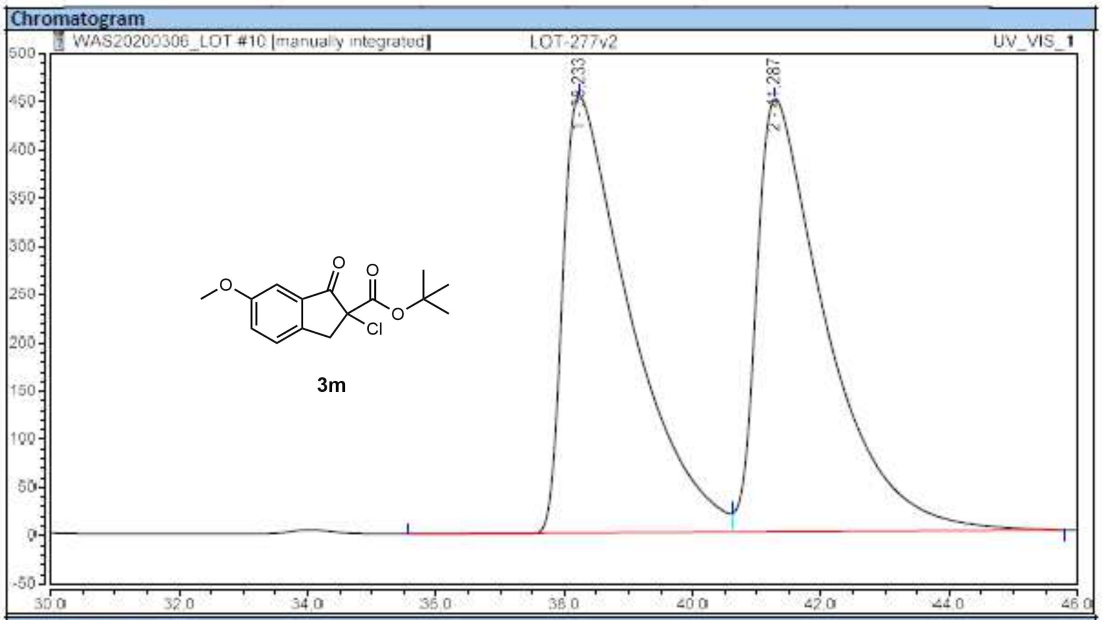

Integration Results

No.	Peak Name	Retention Time min	Area mAU*min	Height mAU	Relative Area %	Relative Height %	Amount n.a.
1		38,233	547,988	453,407	49,35	50,24	n.a.
2		41,287	562,325	449,046	50,65	49,76	n.a.
**Total:**			**1110,313**	**902,453**	**100,00**	**100,00**	

HPLC chromatogram of enantioenriched **3m**:

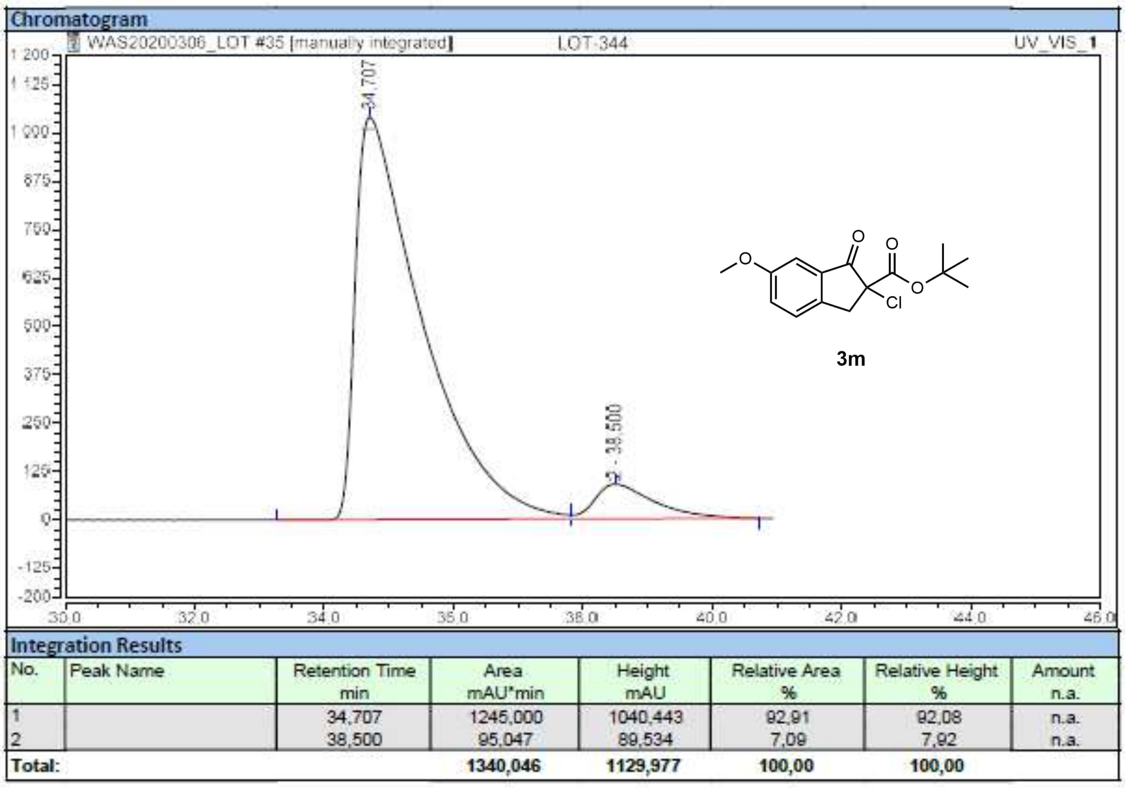

Integration Results

No.	Peak Name	Retention Time min	Area mAU*min	Height mAU	Relative Area %	Relative Height %	Amount n.a.
1		34,707	1245,000	1040,443	92,91	92,08	n.a.
2		38,500	95,047	89,534	7,09	7,92	n.a.
**Total:**			**1340,046**	**1129,977**	**100,00**	**100,00**	

HPLC chromatogram of (*rac*)-**3n**:

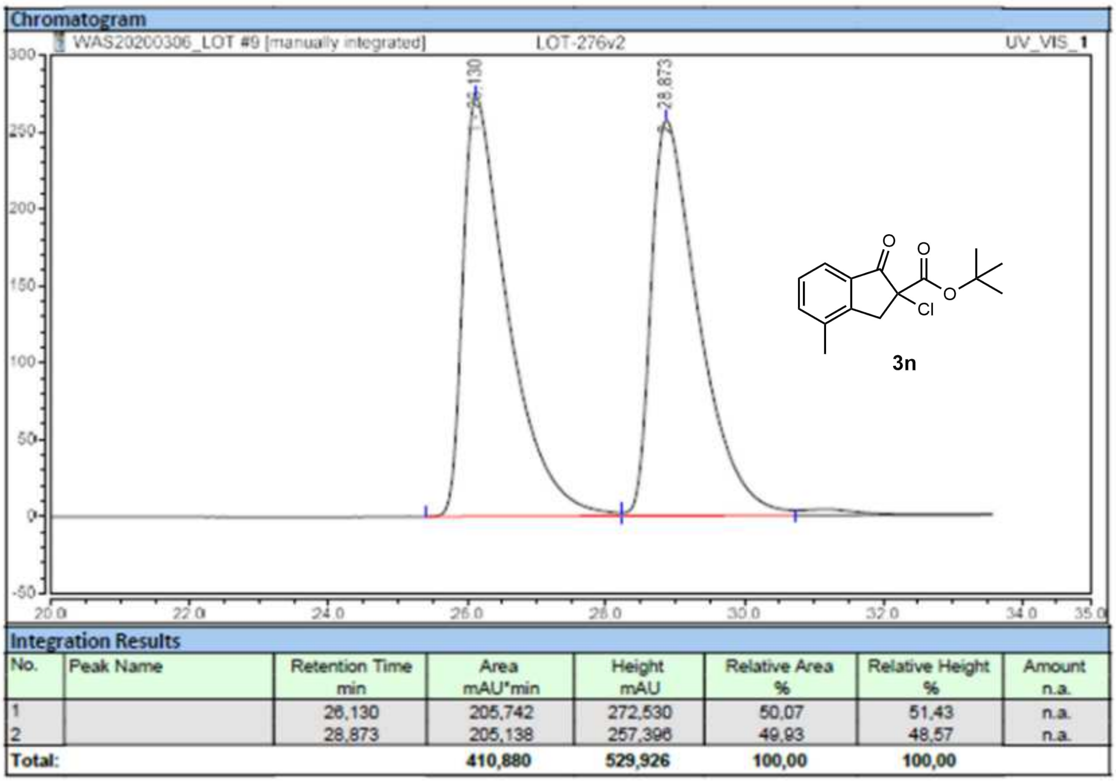

Integration Results

No.	Peak Name	Retention Time min	Area mAU*min	Height mAU	Relative Area %	Relative Height %	Amount n.a.
1		26,130	205,742	272,530	50,07	51,43	n.a.
2		28,873	205,138	257,396	49,93	48,57	n.a.
**Total:**			**410,880**	**529,926**	**100,00**	**100,00**	

HPLC chromatogram of enantioenriched **3n**:

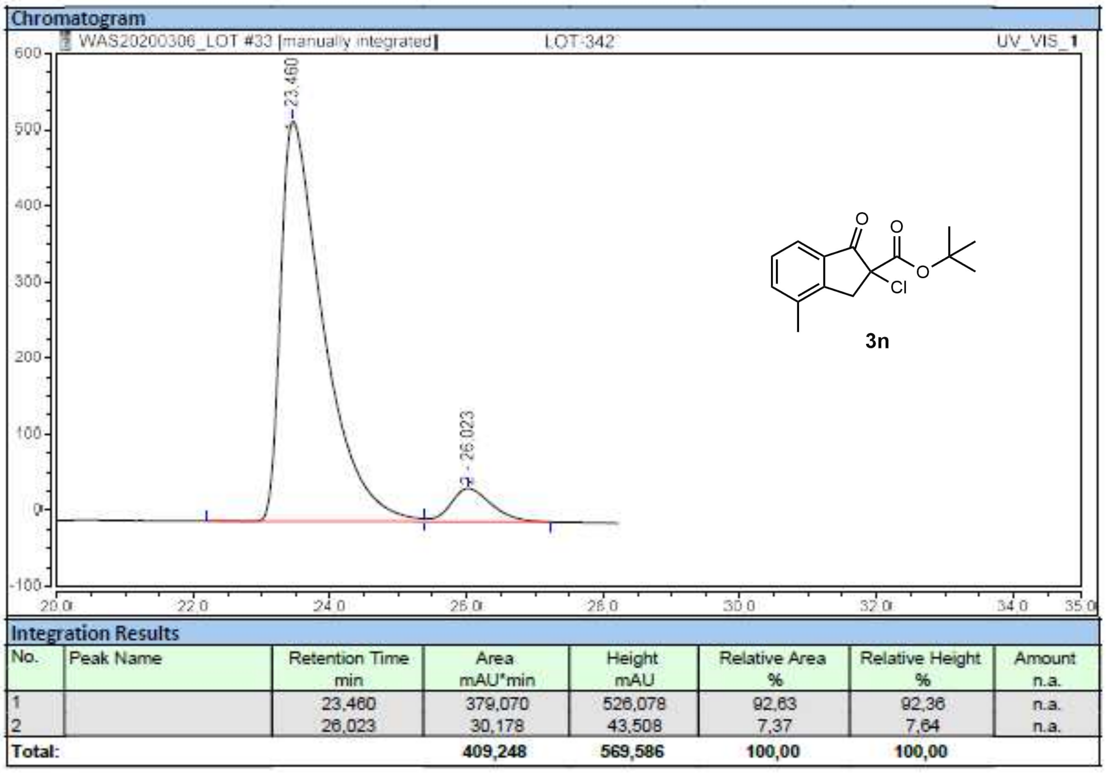

Integration Results

No.	Peak Name	Retention Time min	Area mAU*min	Height mAU	Relative Area %	Relative Height %	Amount n.a.
1		23,460	379,070	526,078	92,63	92,36	n.a.
2		26,023	30,178	43,508	7,37	7,64	n.a.
**Total:**			**409,248**	**569,586**	**100,00**	**100,00**	

HPLC chromatogram of (*rac*)-**3o**:

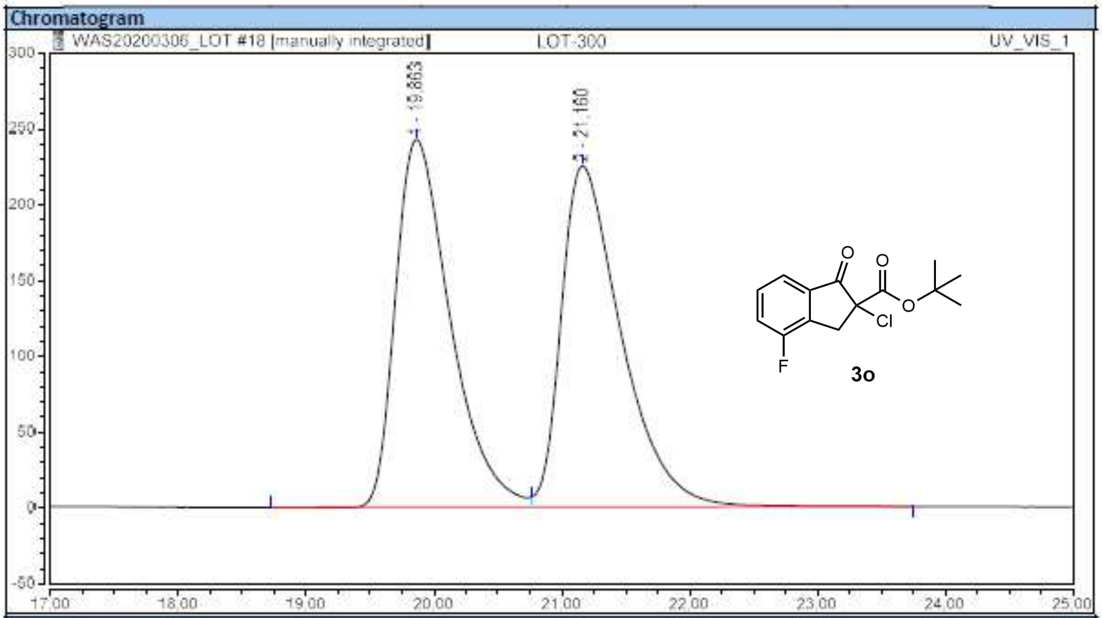

Integration Results

No.	Peak Name	Retention Time min	Area mAU*min	Height mAU	Relative Area %	Relative Height %	Amount n.a.
1		19,863	120,198	242,564	49,54	51,87	n.a.
2		21,160	122,428	225,105	50,46	48,13	n.a.
**Total:**			**242,626**	**467,669**	**100,00**	**100,00**	

HPLC chromatogram of enantioenriched **3o**:

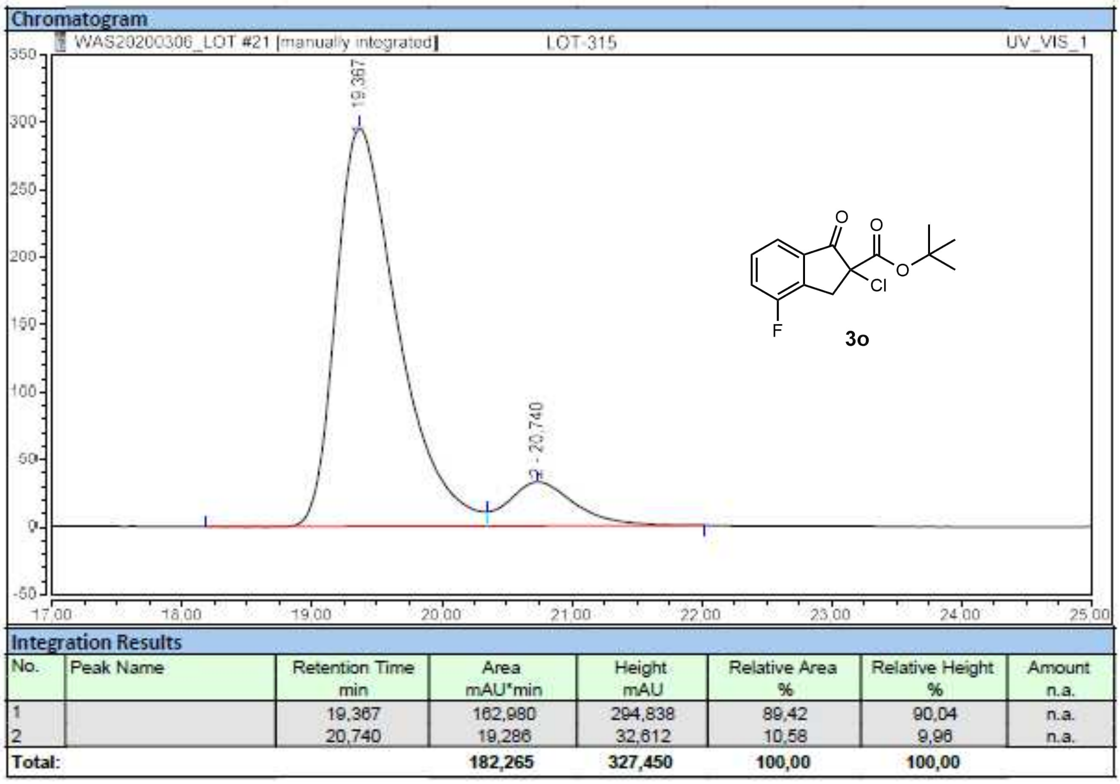

Integration Results

No.	Peak Name	Retention Time min	Area mAU*min	Height mAU	Relative Area %	Relative Height %	Amount n.a.
1		19,367	162,980	294,838	89,42	90,04	n.a.
2		20,740	19,286	32,612	10,58	9,96	n.a.
**Total:**			**182,265**	**327,450**	**100,00**	**100,00**	

HPLC chromatogram of (*rac*)-**3p**:

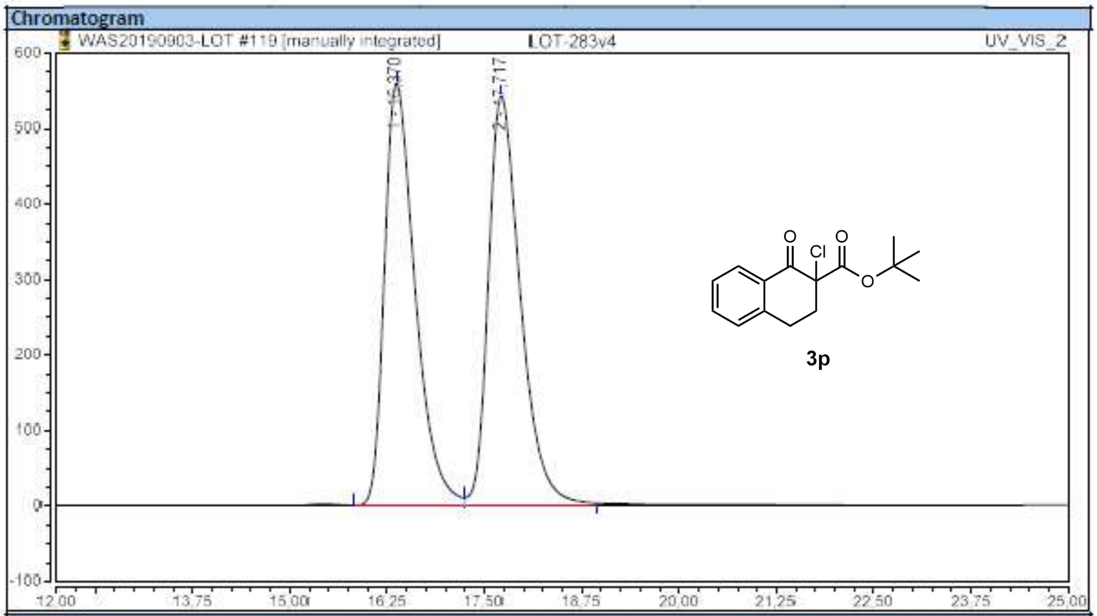

Integration Results

No.	Peak Name	Retention Time min	Area mAU*min	Height mAU	Relative Area %	Relative Height %	Amount n.a.
1		16,370	255,052	560,025	49,53	50,77	n.a.
2		17,717	259,884	543,086	50,47	49,23	n.a.
Total:			514,936	1103,111	100,00	100,00	

HPLC chromatogram of enantioenriched **3p**:

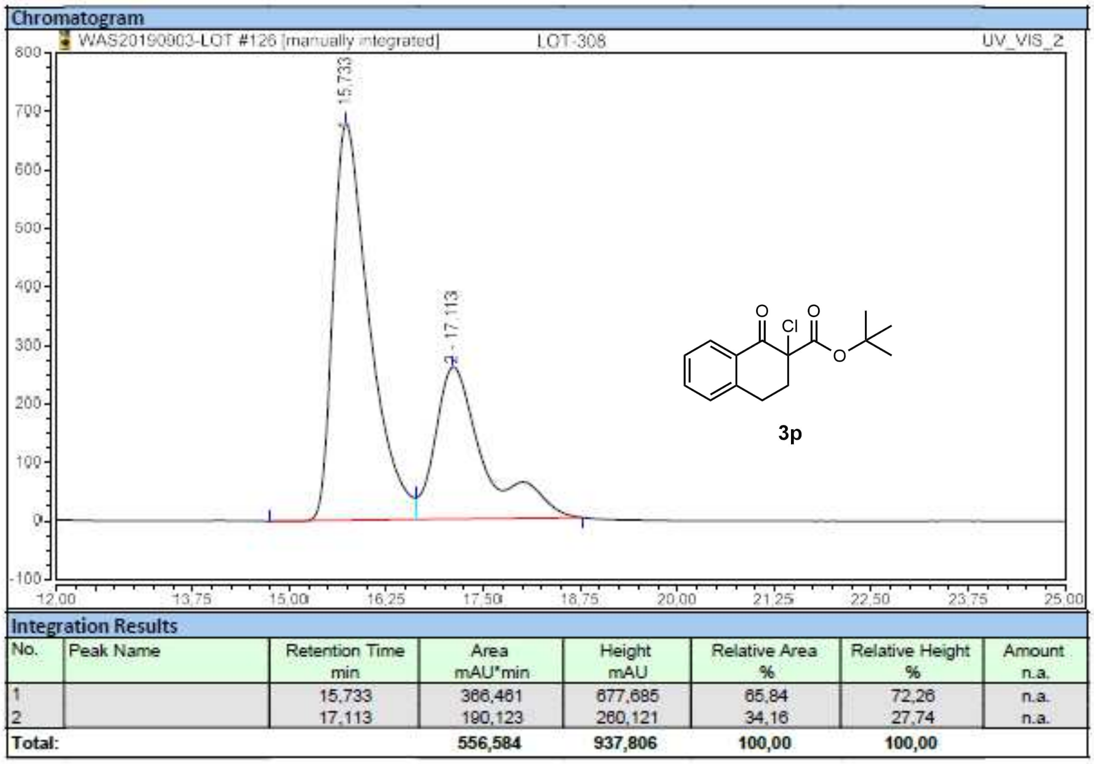

Integration Results

No.	Peak Name	Retention Time min	Area mAU*min	Height mAU	Relative Area %	Relative Height %	Amount n.a.
1		15,733	366,461	677,685	65,84	72,26	n.a.
2		17,113	190,123	260,121	34,16	27,74	n.a.
Total:			556,584	937,806	100,00	100,00	

HPLC chromatogram of (*rac*)-**3q**:

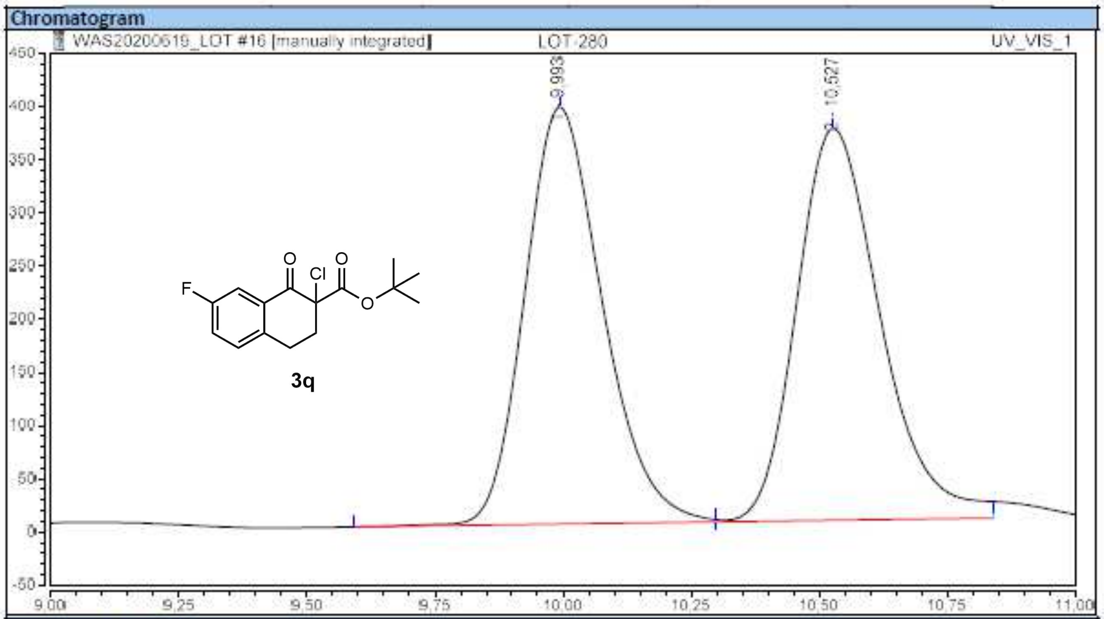

Integration Results

No.	Peak Name	Retention Time min	Area mAU*min	Height mAU	Relative Area %	Relative Height %	Amount n.a.
1		9,993	68,860	391,924	49,69	51,56	n.a.
2		10,527	69,710	368,218	50,31	48,44	n.a.
**Total:**			**138,570**	**760,141**	**100,00**	**100,00**	

HPLC chromatogram of enantioenriched **3q**:

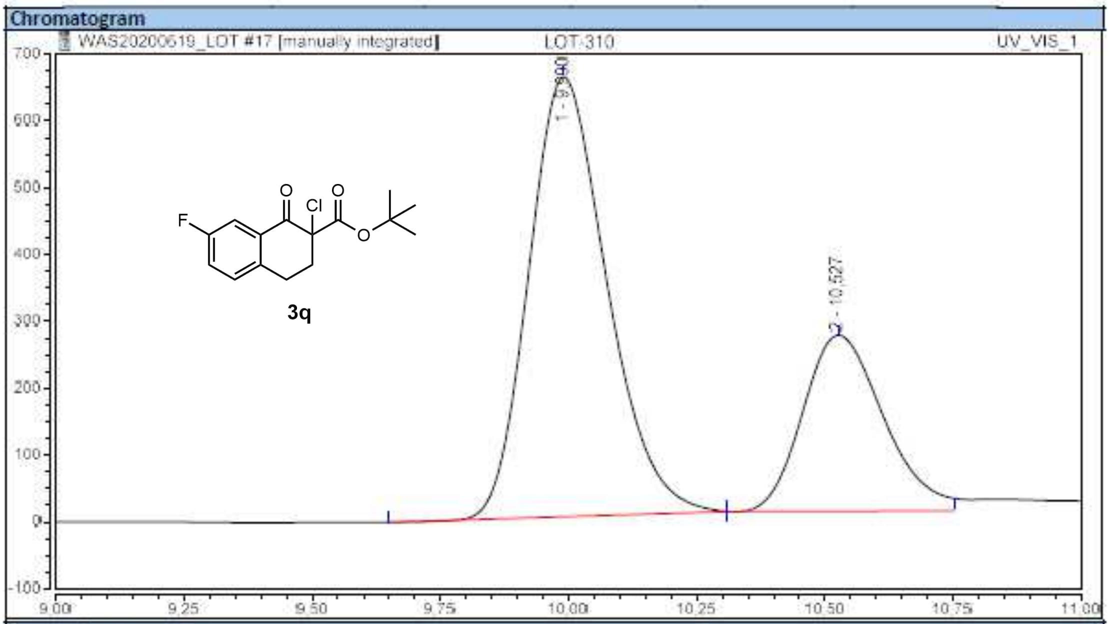

Integration Results

No.	Peak Name	Retention Time min	Area mAU*min	Height mAU	Relative Area %	Relative Height %	Amount n.a.
1		9,990	115,757	658,083	70,73	71,43	n.a.
2		10,527	47,898	263,276	29,27	28,57	n.a.
**Total:**			**163,655**	**921,360**	**100,00**	**100,00**	
